# Deregulation of focal adhesion pathway mediated by miR-659-3p is implicated in bone marrow infiltration of stage M neuroblastoma patients

**DOI:** 10.18632/oncotarget.3745

**Published:** 2015-04-20

**Authors:** Sara Stigliani, Paola Scaruffi, Corrado Lagazio, Luca Persico, Barbara Carlini, Luigi Varesio, Fabio Morandi, Martina Morini, Anna Rita Gigliotti, Maria Rosaria Esposito, Elisabetta Viscardi, Valerio Cecinati, Massimo Conte, Maria Valeria Corrias

**Affiliations:** ^1^ U.O.S. Physiopathology of Human Reproduction, IRCCS A.O.U. San Martino-IST, Genova, Italy; ^2^ Department of Economy, University of Genoa, Genova, Italy; ^3^ Laboratory of Oncology, IRCCS Istituto Giannina Gaslini, Genova, Italy; ^4^ Laboratory of Molecular Biology, IRCCS Istituto Giannina Gaslini, Genova, Italy; ^5^ Epidemiology, Biostatistics and Committees Unit, IRCCS Istituto Giannina Gaslini, Genova, Italy; ^6^ Neuroblastoma Laboratory, Pediatric Research Institute, Fondazione Città della Speranza, Padova, Italy; ^7^ Pediatric Hematology and Oncology Division, Padova University Hospital, Padova, Italy; ^8^ UOS Divisione Oncoematologia Pediatrica, Ospedale Civile di Pescara, Pescara, Italy; ^9^ Oncology Unit, IRCCS Istituto Giannina Gaslini, Genova, Italy

**Keywords:** neuroblastoma, focal adhesion, metastases, bone marrow, miRNA

## Abstract

To get insights on the metastatic process of human neuroblastoma (NB), the miRNA expression profile of bone marrow (BM)-infiltrating cells has been determined and compared to that of primary tumors.

Twenty-two BM-infiltrating cells, 22 primary tumors, and 4 paired samples from patients with metastatic NB aged > 12 months were analyzed for the expression of 670 miRNAs by stem-loop RT-qPCR. The miRNAs whose expression was significantly different were subjected to selection criteria, and 20 selected miRNAs were tested in 10 additional BM-infiltrating cells and primary tumors. Among the miRNAs confirmed to be differentially expressed, miR-659-3p was further analyzed. Transfection of miR-659-3p mimic and inhibitor demonstrated the specific suppression and over-expression, respectively, of the miR-659-3p target gene *CNOT1*, a regulator of transcription of genes containing AU-rich element (ARE) sequence. Among the ARE-containing genes, miR-659-3p mimic and inhibitor specifically modified the expression of *AKT3*, *BCL2*, *CYR61* and *THSB2,* belonging to the focal adhesion pathway. Most importantly, in BM-infiltrating cells *CNOT1* expression was significantly higher, and that of *AKT3*, *BCL2*, *THSB2* and *CYR61* was significantly lower than in primary tumors. Thus, our study suggests a role of the focal adhesion pathway, regulated by miR-659-3p through CNOT1, in the human NB metastatic process.

## INTRODUCTION

The adequate treatment of metastatic cells is becoming one of the most important issues in cancer clinical practice. Differently from all other types of cancer, neuroblastoma (NB) metastases, frequently found in highly vascular tissues as bone marrow (BM) and bone, are present since diagnosis [1]. Patients with metastatic NB (stage M) aged more than 12 months at diagnosis are considered high-risk patients, whose overall survival at 5 years is 25–30%, despite multimodal aggressive therapy [2]. Therefore, the search for new therapeutic approaches is warrant. However, little is known regarding the metastatic process in NB patients.

Several *in vitro* studies on NB cell lines suggested a pivotal role of the CXCL12-CXCR4 axis in BM infiltration [3–6], but the demonstrations that CXCR4 is not functional in BM-infiltrating NB cells [7], and that BM resident cells in NB patients have significantly lower expression of CXCL12 than in healthy children [8], do not support a role for this axis in stage M NB patients.

BM-infiltrating NB cells show the same genetic characteristics of primary tumor cells [9], but we previously demonstrated that they differentially express several genes [10]. In particular, metastatic cells over-express HLA-G and calprotectin that are responsible for an immune suppressive status and for the sustained inflammation of the BM microenvironment, respectively [8]. Moreover, BM-infiltrating NB cells under-express several genes, such as *CX3CL1* (also called fractalkine), that is involved in *in vitro* transmigration through BM endothelial cells [11].

To further characterize the differential features of BM-infiltrating and primary tumor NB cells, we focused on their miRNA expression profiles. MiRNAs are 18-22 base long RNAs whose role in mediating important physiological and pathological processes, including cancer progression and metastasis, has been widely documented. *In vitro* studies in NB cell lines identified several miRNAs as regulators of different pathways [12–22], but information regarding miRNA expression in patients' tissues are limited. Four miRNA signatures of human NB primary tumors were demonstrated to predict prognosis [23–26]. However, only a 25 miRNA signature is prognostic within the subset of stage M patients [25]. No miRNA signature of human BM-infiltrating NB cells has been determined so far. A signature of differentially expressed miRNAs was determined by Guo and coworkers by comparing subcutaneous tumors and their metastases grown in nude mice following injection of a human NB cell line [27].

We thus analyzed the miRNA profiles of human BM-infiltrating cells and primary tumors to identify the miRNAs differentially expressed and potentially involved in the metastatic process. After screening the significant miRNAs for level and distribution of expression values in the two groups of samples, we focused on miR-659-3p. Studies in NB cell lines treated with miR-659-3p mimic and inhibitor indicated that miR-659-3p specifically modifies the expression of the transcription factor *CNOT1*, and the expression of the AU-rich element (ARE)-containing target genes *AKT3*, *BCL2, CYR61* and *THSB2,* that belong to the focal adhesion pathway. Indeed, BM-infiltrating cells express lower level of miR-659-3p, higher level of *CNOT1* and lower levels of *AKT3*, *BCL2, THSB2* and *CYR61* than NB primary tumors. Our finding may pave the way to the development of new therapeutic strategies focused on targeting the metastatic process.

## RESULTS

### MiRNA profiling of NB BM-infiltrating cells and primary tumors

**Figure 1:**
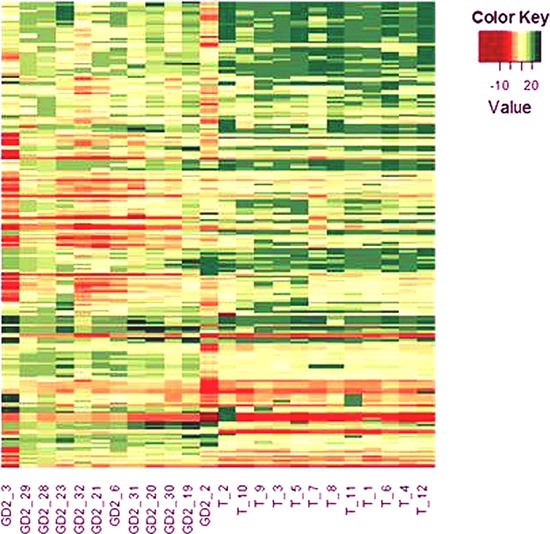
Heat-map of miRNAs differentially expressed by BM-infiltrating cells and primary tumors The miRNA expression profiles of 12 BM-infiltrating cells and 12 primary tumors were analyzed by R of Bioconductor and the miRNAs differentially expressed by the two groups of samples with an adjusted *p* value < 0.05 are shown. The miRNA codes are reported in Supplementary Table 1.

First, twelve BM-infiltrating cells and twelve primary tumors were randomly selected from our bio-bank. Patients' characteristics are reported in Table 1. Each sample was tested for the expression of 670 different miRNAs by stem-loop RT-qPCR amplification of human miRNA cards. After data normalization using the small U6 RNA as endogenous reference, 160 miRNAs were found to be differentially expressed between metastases and primary tumors with an adjusted *p* value < 0.05 (Figure 1 and Supplementary Table 1). The expression of 42 (26%) miRNAs was lower and that of 118 was higher in BM-infiltrating cells than in primary tumors. No significant differences were observed when the samples were stratified according to *MYCN* status.

**Table 1:** Demographic, biological and clinical characteristics of the study patients

Patients' characteristics	*First set of samples*		*Second set of samples*		*Paired samples*
	BM-infiltrating cells (*N* = 12)	Primary tumors (*N* = 12)	BM-infiltrating cells (*N* = 10)	Primary tumors (*N* = 10)	BM-infiltrating and primary tumors (*N* = 4)
*Age (median, years)*	2.6	3.9	2.8	2.6	2.5
Range	1.9 – 6.0	1.9 – 6.9	1.7 – 6.9	1.5 – 6.3	1.7 – 3.3
*Sex*					
Female	5		5	5	1
Male	7	6	5	5	3
*MYCN status*					
Not-amplified	6	6	2	4	
Amplified	6	6	8	6	3
*Primary site*					
Adrenal	5	8	5	6	2
Abdomen	5	1		2	1
Thorax	2	3	1	2	1
*Metastatic sites*					
Bone marrow + Bone	12	8	3	7	4
Bone marrow		3	6	2	
Liver	2	1	1	1	1
Lymph nodes		1			1
*Relapse/Progression*					
No	3	2	6	2	1
Yes	9	10	4	8	3
*Present clinical status*					
Alive	2	3	6	2	2
Dead of disease	10	9	4	8	2

To reduce the number of miRNAs to be validated and further investigated, additional selection criteria were applied. First, we excluded significant miRNAs which were not expressed in some samples of both groups, unless the number of samples with no expression in one group was at least twice the number of samples with no expression in the other group. Fifty-three out of the 160 differentially expressed miRNAs (14 less and 39 more expressed in BM-infiltrating cells than in primary tumors) passed this first selection (Supplementary Table 2). Then, direct inspection of the expression levels' distribution was performed to identify the significant miRNAs with the highest differences between BM-infiltrating cells and primary tumors. Precisely, only miRNAs for which the first quartile of expression levels in one of the two groups was higher than the third quartile in the other group were considered. Twenty miRNAs, 4 less and 16 more expressed in BM-infiltrating cells than in primary tumors, passed this second selection criterion (Table 2).

**Table 2:** Expression values of the selected 20 miRNAs

miRNA^a^	First set (*N* = 12)			Second set (*N* = 10)		
	BM-infiltrating cells ΔCq	Primary tumors ΔCq	*p* value	BM-infiltrating cells ΔCq	Primary tumors ΔCq	*p* value
hsa-miR-324-3p	7.416	0.589	*1.54E-03*	4.135	2.510	*2.88E-02*
**hsa-miR-516a-3p**	18.105	12.181	*6.54E-03*	15.780	13.410	*3.55E-02*
hsa-miR-628-5p	9.035	1.510	*1.54E-03*	6.745	6.195	*7.96E-01*
**hsa-miR-659-3p**	18.751	10.829	*1.54E-03*	15.285	10.255	*1.08E-05*
hsa-miR-10b-5p	4.244	10.208	*5.71E-04*	0.785	2.960	*1.05E-01*
hsa-miR-128	6.384	10.403	*1.18E-02*	6.805	7.045	*3.15E-01*
hsa-miR-137	4.720	12.183	*1.72E-03*	4.790	8.135	*1.85E-02*
hsa-miR-140-5p	4.083	11.724	*6.90E-03*	4.100	6.660	*5.24E-02*
hsa-miR-16-5p	1.874	7.497	*1.83E-03*	0.790	2.720	*8.20E-02*
hsa-miR-191-5p	2.064	5.283	*5.45E-03*	1.160	1.865	*4.81E-01*
hsa-miR-301a-3p	4.556	12.233	*5.45E-03*	4.140	6.915	*2.88E-03*
**hsa-miR-361-3p**	14.231	22.536	*5.46E-04*	15.640	21.660	*2.90E-01*
hsa-miR-365a-3p	4.421	11.826	*3.07E-03*	7.235	8.300	*1.05E-01*
hsa-miR-548d-3p	18.164	22.702	*1.72E-03*	13.450	14.340	*2.18E-01*
hsa-miR-572	14.818	22.249	*9.48E-03*	12.835	12.225	*6.31E-01*
hsa-miR-576-5p	16.230	22.240	*6.54E-03*	15.360	16.080	*2.80E-01*
hsa-miR-616-5p	16.251	22.693	*1.62E-04*	13.955	15.535	*1.51E-01*
hsa-miR-628-3p	12.810	22.910	*4.71E-06*	12.520	14.670	*1.65E-01*
hsa-miR-873-5p	14.074	22.058	*6.54E-03*	13.270	17.470	*2.32E-02*
hsa-miR-98-5p	9.393	21.682	*1.83E-03*	10.625	10.050	*9.71E-01*

a miRNAs found differentially expressed also in paired samples are indicated in bold.

The expression of these 20 miRNAs was then tested by RT-qPCR in additional 10 BM-infiltrating cells and 10 primary tumors, using the specific miRNA assays in 96 well plates in triplicate. Six miRNAs, 3 less expressed (miR-324-3p, miR-516a-3p, miR-659-3p) and 3 more expressed (miR-137, miR-301a-3p, miR-873-5p) in BM-infiltrating cells than in primary tumors, were significantly differentially expressed also in this new set of samples (Table 2).

Next, we analyzed the expression of all 670 miRNAs in 4 paired BM-infiltrating cells and primary tumors from 4 additional patients (Table 1). Although the power (sensitivity) of paired sample *t* test was low, the analysis revealed that the BM-infiltrating cells and the corresponding primary tumors differentially expressed 51 miRNAs, with 18 (35%) less expressed in the metastases.

Of these 51 miRNAs, eight were in common with the 53 miRNAs that passed the first selection criterion (Supplementary Table 2, in bold) and three with the 20 miRNAs that passed also the second selection (Table 2, in bold). Of these three miRNAs, miR-516a-3p and miR-659-3p were significantly less expressed in BM-infiltrating cells than in primary tumors in all sets of samples (Table 2).

### MiRNA profiling of NB cell lines and adrenal tissue

To understand whether miR-516a-3p or miR-659-3p deserved further studies, the miRNA profiles of two NB cell lines, the *MYCN* amplified HTLA-230 and the *MYCN* non amplified SH-SY5Y, were evaluated together with the miRNA profile of the normal adrenal gland. Comparison of the three profiles showed no differences for 482 miRNAs (Supplementary Table 3). Ninety miRNAs were differentially expressed by HTLA-230 and SH-SY5Y, likely because of the different *MYCN* status (Supplementary Table 4). Ninety-eight miRNAs were differentially expressed by normal adrenal gland and NB cell lines (Supplementary Table 5), supporting their putative implication in the neoplastic process.

Since miR-516a-3p was expressed at the same level in the normal adrenal gland and NB cell lines, whereas miR-659-3p was not expressed by the adrenal gland and highly expressed in NB cell lines, we selected this latter miRNA for further studies.

### Effects of miR-659-3p over-expression and suppression on gene expression

**Figure 2:**
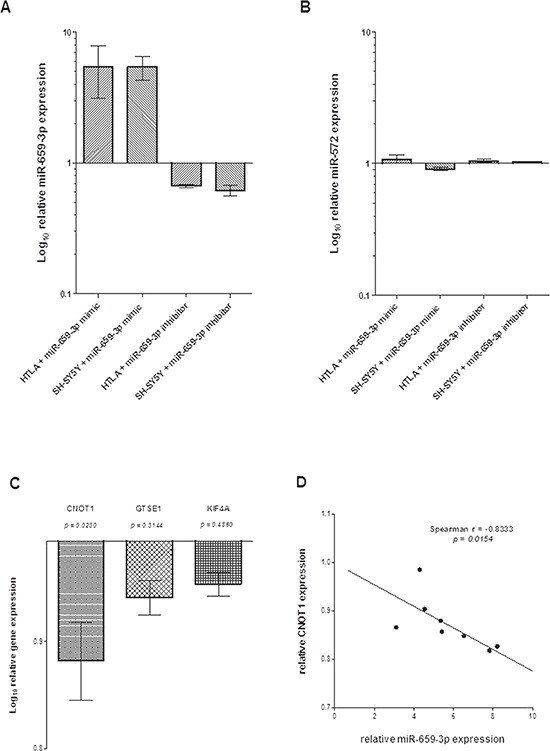
(A) Expression of miR-659-3p, (B) miR-572, (C) *CNOT1, GSTE1* and *KIF4A* and (D) inverse correlation between miR-659-3p and *CNOT1* expression in HTLA-230 and SH-SY5Y cells treated with miR-659-3p mimic and inhibitor Results of RT-qPCR analysis from two independent experiments are reported as Log_10_ of relative expression respect to that occurring in cells treated with irrelevant miRNA mimic and inhibitor. Standard errors are indicated. The RT-qPCR data were normalized using U6 small RNA (panel A and B) and *HPRT1* gene (panel C) as reference.

To evaluate the role of miR-659-3p and its target genes in the metastatic process, the HTLA-230 and SH-SY5Y NB cell lines were transfected with specific miR-659-3p or irrelevant miRNA mimic and inhibitor. As shown in Figure 2A, treatment with miR-659-3p mimic and inhibitor respectively increased and decreased miR-659-3p expression as compared to cells treated with the irrelevant mimic and inhibitor, whereas the expression of the unrelated miR-572 was unaffected (Figure 2B).

To identify miR-659-3p target genes, the whole genome expression profiles of HTLA-230 and SH-SY5Y cells transfected with miR-659-3p mimic and inhibitor were compared to those of cells transfected with irrelevant miRNA mimic and inhibitor, respectively. Statistical analysis of the eight profiles demonstrated that the expression of 2073 probes was specifically and inversely modified by miR-659-3p mimic and inhibitor (Supplementary Data). These 2073 probes were merged with the probes differentially expressed by BM-infiltrating cells and primary tumors ([10], GEO accession number GSE 25623a). Thirty-four genes more expressed in BM-infiltrating cells than primary tumors were also more expressed in the NB cell lines treated with the miR-659-3p inhibitor (Table 3). These genes were checked for the presence of 3′-UTR sequence recognized by miR-659-3p by TargetScan (www.targetscan.org), and 3 genes (*CNOT1*, *GTSE1* and *KIF4A*) were identified.

**Table 3:** Genes specifically modulated by treatment with miR-659-3p mimic, known to be differentially expressed by BM-infiltrating cells and primary tumors

Down-modulated genes^a^	Up-modulated genes^b^	
*AI457687*	*ADAMTS9-AS2*	*OXCT2*
*ARHGAP15*	*ADRA1B*	*PAQR8*
*ASNA1*	*AHCYL2*	*PHYHD1*
*BE696323*	*AKT3*	*PRCD*
*BF089603*	*AQP1*	*RAB6B*
*BX360933*	*ATP2B4*	*RALA*
*BX415272*	*BCL2*	*RNASEL*
*CDC20*	*CACNG3*	*SEPT4*
*CDK4*	*CACUL1*	*SIRT3*
*CNOT1*	*CCL14*	*SLC16A11*
*EIF2C2*	*CGNL1*	*STON1*
*EIF4B*	*CNKSR3*	*TFF1*
*GTSE1*	*COL27A1*	*THBS2*
*KIF4A*	*CXCL14*	*USP27X*
*MAP2K3*	*CYR61*	*USP47*
*MAPK14*	*DST*	*VWA1*
*ORM2*	*FLT4*	*ZNF652*
*PIGA*	*GABRE*	
*PPP2R2A*	*GFOD1*	
*PRAM1*	*GIMAP5*	
*RHOA*	*HEY1*	
*SBNO1*	*HSPA4*	
*SMC1A*	*IKZF4*	
*SNRPA*	*IRF2BP2*	
*SP1*	*KCNJ8*	
*STXBP2*	*LPHN3*	
*THC2270231*	*LRRC32*	
*THC2287287*	*LYZL1*	
*THC2415133*	*MCAM*	
*THC2455389*	*MOB3B*	
*UBE2S*	*MYOG*	
*VPS25*	*NKD2*	
*WDR51A*	*ODF3L2*	
*ZDHHC3*	*OLFM1*	

a Genes down-modulated following miR-659-3p over-expression, up-regulated following miR-659-3p suppression, up-modulated in BM-infiltrating cells.

b Genes up-modulated following miR-659-3p over-expression, down-modulated following miR-659-3p suppression, down-modulated in BM-infiltrating cells.

As shown in Figure 2C, RT-qPCR analysis confirmed that *CNOT1* expression was specifically and significantly modified by miR-659-3p mimic and inhibitor treatment, whereas *GTSE1* and *KIF4A* expression was not affected by the treatment. Moreover, in NB cell lines treated with miR-659-3p mimic and inhibitor, *CNOT1* expression levels inversely correlated to miR-659-3p expression levels (Figure 2D). Taken together, these findings demonstrated that *CNOT1* expression in NB cells was specifically modified by miR-659-3p.

Since *CNOT1* encodes a transcription factor that degrades mRNAs containing ARE sequences [28], over-expression of *CNOT1,* subsequent to decreased expression of miR-659-3p, should reduce the expression of ARE-containing genes. We thus checked the 51 genes differentially expressed by BM-infiltrating cells and primary tumors, and over-expressed in NB cell lines treated with miR-659-3p mimic (Table 3), for the presence of ARE sequence by consulting the http://brp.kfshrc.edu.sa/ARED website.

**Figure 3:**
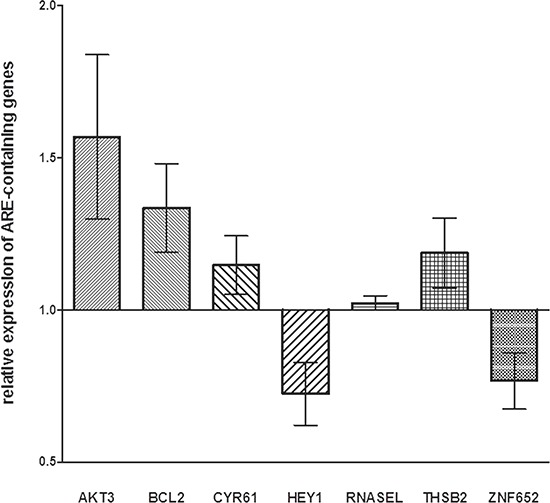
Expression levels of genes containing ARE-sequence in HTLA-230 and SH-SY5Y cells treated with miR-659-3p mimic and inhibitor Results of RT-qPCR analysis from two independent experiments are reported as Log_10_ of relative expression respect to that occurring in cells treated with irrelevant miRNA mimic and inhibitor. Standard errors are indicated. All p values were not significant. The RT-qPCR data were normalized using *HPRT1* as reference gene.

Twelve genes (*AHCYL2*, *AKT3*, *BCL2*, *CYR61*, *DST*, *GFOD1*, *HEY1*, *IKZF4*, *LPHN3*, *RNASEL*, *THBS2*, *and ZNF652*) were identified. In the miR-659-3p-transfected NB cell lines, the expression of *AKT3*, *BCL2, CYR61* and *THSB2* was confirmed to be specifically modified by miR-659-3p mimic and inhibitor, whereas that of *RNASEL* was not affected by the treatment and that of *HEY1* and *ZNF652* occurred in the wrong direction (Figure 3).

### Validation of differential expression of *CNOT1, AKT3, BCL2, CYR61* and *THBS2* genes in BM-infiltrating cells and primary tumors

**Figure 4:**
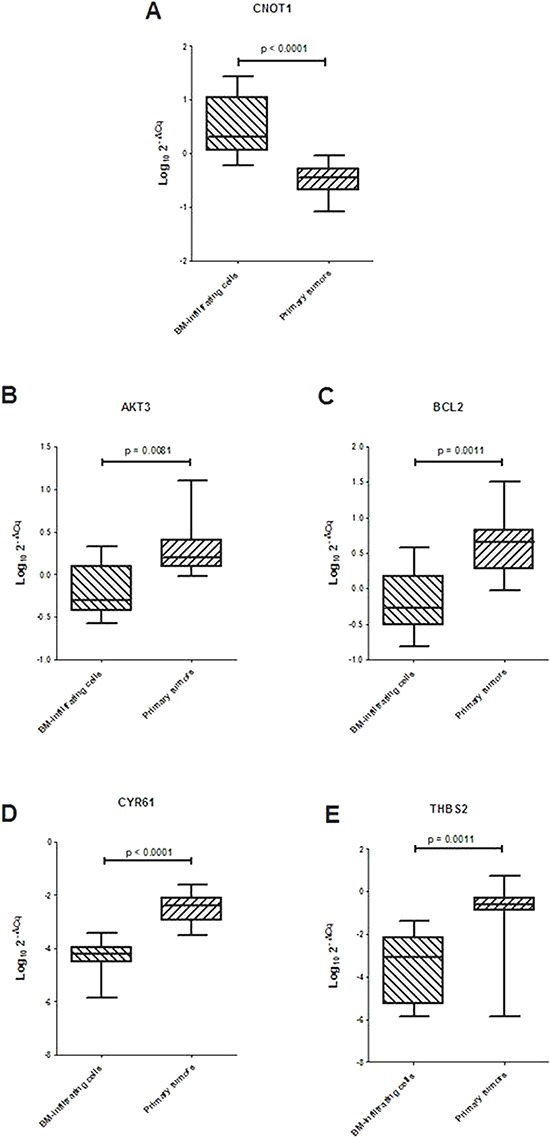
(A) Expression levels of CNOT1 and (B–E) ARE-sequence containing genes involved in focal adhesion pathway in 10 BM-infiltrating and 10 primary tumors Data are expressed as Log_10_ 2^−ΔCq^ values, were ΔCq is the difference between the Cq for the target gene and the Cq for the reference *HPRT1* gene. Horizontal bars in the box-plots indicate medians. *P* values are indicated.

Gene ontology search for *AKT3, BCL2, CYR61* and *THBS2* genes in the http://david.abcc.ncifcrf.gov/ website indicated that they belong to the focal adhesion pathway. Thus, ten BM-infiltrating cells and ten primary tumors were analyzed by RT-qPCR to confirm that miR-659-3p reduced expression in BM-infiltrating cells associated to over-expression of *CNOT1* and reduced expression of *AKT3, BCL2, CYR61* and *THBS2* genes, as compared to primary tumors. Indeed, in BM-infiltrating cells, *CNOT1* expression was significantly higher, and *AKT3*, *BCL2, CYR61* and *THSB2* expressions were all significantly lower than in primary tumors (Figure 4). This finding supports the hypothesis that the metastatic process in human NB involves alteration of the focal adhesion pathway by regulation of *CNOT1* levels determined in turn by miR-659-3p expression level.

## DISCUSSION

In all sets of samples from stage M NB patients, miR-659-3p expression resulted significantly lower in BM-infiltrating cells than in primary tumors. Several miRNAs other than miR-659-3p differentially expressed by NB tumors and metastases may deserve further attention as potential drivers of the metastatic process. In particular, the miRNAs previously shown to influence migration and invasion *in vitro* and in murine models [12,16,18,22].

Nonetheless, in this first report on miRNA profile of human metastatic BM-infiltrating NB cells and on its comparison to that of human NB primary tumors, we decided to focus on the miRNA that was unequivocally differentially expressed by the two groups. Comparison of 670 miRNA expression levels in a first set of BM-infiltrating and primary tumors indicated that 160 miRNAs were significantly differently expressed in the two groups of samples. Interestingly, 14 miRNAs over-expressed in BM-infiltrating cells were in common with those differentially expressed in a metastatic murine xenograft model, obtained by injection of a human NB cell line in nude mice [27]. However, 6 of these miRNAs were more expressed in the subcutaneous tumors and 8 over-expressed in the murine metastases. Among these latter miRNAs, miR-23a and miR-15 promoted *in vitro* migration of NB cell lines by targeting *CDH1* [20] and *RECK* [22], respectively. Conversely, miR-335 suppressed invasion through the TGF-β pathway [16], and miR-9 and miR-542-5p over-expression in human NB primary tumors associated to a better survival [12,18,23], making unlikely a positive role in the human metastatic process.

Variability in the results obtained in different settings can be due to methodological reasons, such as the type of samples (human cells *versus* xenografts), miRNA analysis (array *versus* RT-qPCR) and normalization procedures (multiple *versus* single miRNA), just to name a few. An additional cause can be the wide range of miRNA expression levels observed in both BM-infiltrating cells and primary tumors. Heterogeneity of miRNA expression in human primary tumors has been previously reported [23–25], and phenotypic and genetic NB heterogeneity is a well-known feature of this pediatric cancer [29]. Since the heterogeneity occurs also within the high-risk stage M subset, generalization of findings in human NB is difficult [30]. Therefore, it is plausible that differences in miRNA expression levels between *MYCN-*amplified and -no amplified tumors previously observed by others [13,15,19] and not confirmed here were hidden by the wide variability.

Here, the analysis of ten additional BM-infiltrating and primary tumors and four paired BM-infiltrating cells and corresponding primary tumors demonstrated that miR-659-3p was always less expressed in human NB metastases as compared to primary tumors. In addition, miR-659-3p was not expressed in normal adrenal gland, supporting its role in the neoplastic process. *In vitro* experiments with miR-659-3p mimic and inhibitor allowed to identify *CNOT1* as its target gene in human NB. CNOT1 (CCR4-NOT transcription complex, subunit 1), fundamental for maintenance of embryonic stem cell identity [28], is a negative regulator of transcription because it degrades mRNAs containing ARE sequences [31]. The higher expression of *CNOT1* driven by the lower expression of miR-659-3p leaded to reduced expression of four ARE-containing mRNAs, *AKT3, BCL2, CYR61* and *THBS2,* belonging to the focal adhesion pathway. Indeed, in BM-infiltrating cells, the lower expression of miR-659-3p associated to higher levels of *CNOT1* and lower expression of *AKT3, BCL2, CYR61* and *THBS2* mRNAs, as compared to primary tumors.

The differential expression of these genes was already reported by us [10], but their relationships were unknown, as well as with miR-659-3p. *AKT3* codes for a member of the AKT serine/threonine protein kinase family. AKT kinases are regulators of cell signaling in response to platelet-derived growth factor (PDGF), insulin, and insulin-like growth factor 1 (IGF1). AKT3 exerts negative effects on tumor endothelial cell growth and migration by inhibiting the activation of translation regulatory kinase S6K [32]. *BCL2* codes for an integral outer mitochondrial membrane protein that blocks apoptotic death [33], *CYR61* codes for a protein that interact with several integrins and heparin-sulfate proteoglycans [34], and *THSB2* codes for thrombospondin 2, a disulfide-linked homo-trimeric glycoprotein that mediates cell-to-cell and cell-to-matrix interactions and strongly inhibits invasion and metastases [35]. Thus, the products of these genes suppress angiogenesis and proliferation and inhibit tumor cell invasion and metastases.

## CONCLUSIONS

Taken together the results of our study support the hypothesis that the metastatic process in human NB involves alteration of the focal adhesion pathway, through the increased expression of *CNOT1*, driven by decreased expression of miR-659-3p. Thus, avoiding the disruption of the structural architecture by increasing miR-659-3p expression may dampen the metastatic process. Adjuvant therapy with AKT activators or metallo-protease inhibitors, together with targeted delivery of miR-659-3p mimic, could be considered in relapsed or refractory stage M NB patients.

## METHODS

### BM-infiltrating NB cells and NB primary tumor cells

Twenty-two BM-infiltrating GD2+ cell samples were immune-selected at diagnosis, as previously described [8]. Simultaneously, 22 frozen primary tumor specimens, containing more than 80% NB cells, were selected from our bio-bank to possibly match the demographic, biological and clinical characteristics of the BM-infiltrating samples. All samples belonged to stage M [2] NB patients aged more than 12 months at diagnosis. Each group of samples was then randomly divided. Twelve BM-infiltrating NB cells and twelve primary tumors were used to determine the complete miRNA profiling and identify the miRNAs whose expression was significantly different between primary tumors and metastases.

After reducing the number of miRNAs to be validated by applying two selection criteria (see statistical analysis for details), 20 significant differentially expressed miRNAs were evaluated in the remaining ten BM-infiltrating cells and ten primary tumors. In addition, paired BM-infiltrating NB cells and corresponding primary tumors from 4 stage M patients were subjected to complete miRNA profiling to confirm the differential expression of the validated miRNAs. The demographic, biological and clinical characteristics of the patients included in the study, retrieved from the Italian Neuroblastoma Registry [36], are reported in Table 1.

### NB cell lines and transfection experiments

HTLA-230 (kindly donated by Dr E. Bogenman, Los Angeles, USA) and SH-SY5Y (purchased from Banca Biologica and Cell Factory, Genoa, Italy) NB cell lines were cultured in RPMI 1640 medium supplemented with 10% heat inactivated FCS, 50 mg/ml streptomycin, 50 mg/ml penicillin and 2 mM glutamine (Sigma-Aldrich, Milan, Italy). The HTLA-230 cells present *MYCN* amplification and the SH-SY5Y cells have normal *MYCN* status. Both cell lines were checked for morphology, proliferation rate, mycoplasma contamination and *MYCN* amplification after thawing and within four passages in culture.

To evaluate the effect of miR-659-3p over-expression and suppression on gene expression, 1×10^6^ HTLA-230 and SH-SY5Y cells were transfected with specific miR-659-3p mirVana™ mimic and inhibitor, respectively (Ambion, Life Technologies, Carlsbad, CA, USA, catalog# MC and MH 11582). Samples transfected with mirVana™ miRNA Mimic Negative Control #1 and mirVana™ miRNA Inhibitor Negative Control #1 were used as reference conditions. Transfection was performed at 20 nM miRNA concentration in OptiMEM^©^ medium (Sigma-Aldrich), using Lipofectamin RNAiMAX^©^ (Life Technologies), according to manufacturer's protocol. Cells were then cultured for 48 hours, checked for viability and processed for miRNA and total mRNA extraction, as described below. Transfection experiments were performed twice.

### RNA extraction

Total RNA and miRNA fractions were extracted from BM-infiltrating cells, primary tumors and transfected NB cell lines using the miRNeasy Mini kit (Qiagen, Hilden, Germany), according to manufacturer's protocols. Total RNA and miRNA fractions from whole adrenal gland (cortex + medulla) were purchased from Ambion^©^ (Life Technologies). Quality of the RNA fractions was evaluated by RNA 6000 Nano kit in the BioAnalyzer 2100 system (Agilent Technologies, Santa Clara, CA, USA).

### MiRNA profiling

The miRNA fraction of each sample was subjected to stem-loop RT-qPCR amplification, as described [37]. Precisely, 30 ng of the miRNA fraction were reverse transcribed using the Megaplex RT Primers Human Pool A and B. At the end of the reaction, each RT product was amplified with the Megaplex PreAmp Primers A and B for 25 cycles. Then, the amplification products were loaded onto MicroRNA TaqMan Card A and B, respectively. Card amplifications were performed on ViiA7 equipment for 40 cycles. All reagents and equipment were from Life Technologies. Results were expressed as delta Cq by subtracting the Cq value obtained for U6 small RNA from the Cq value of each miRNA. For unexpressed miRNAs the Cq value was set at 40.

To validate the differential expression of the selected 20 miRNAs, reverse-transcribed and pre-amplified miRNA fractions from 10 additional BM-infiltrating and 10 primary tumors were amplified in a 96 well plate in triplicate using the specific TaqMan^©^ human microRNA assays (hsa-miR-324-3p, catalog #002161; hsa-miR-516-3p, catalog #001149; hsa-miR-628-5p, catalog #002433; hsa-miR-659-3p, catalog #001514; hsa-miR-10b, catalog #002218; hsa-miR-128, catalog #002216; mmu-miR-137, catalog #01129; mmu-miR-140, catalog #001187; hsa-miR-16, catalog #000391, hsa-miR-191, catalog #002299; hsa-miR-301, catalog #000528; hsa-miR-361-3p, catalog #002116; hsa-miR-365, catalog #001020; hsa-miR-548d-3p, catalog #001605; hsa-miR-572, catalog #001614; hsa-miR-576-5p, catalog #002350, hsa-miR-616, catalog #001589; hsa-miR-628-3p, catalog #002434; hsa-miR-873, catalog #002356; hsa-miR-98, catalog #000577; U6 snRNA, catalog #001973, Life Technologies). Amplification with U6 assay was used as endogenous reference and results were expressed as delta Cq, as described above.

To confirm increase and decrease of miR-659-3p expression following transfection with miR- mimic and inhibitor, respectively, the miRNA fraction isolated from each transfected cells was reverse transcribed using the Megaplex RT Primers Human Pool A and B and then amplified with the TaqMan^©^ human miR-659-3p assay in triplicates in a 96 well plate. As control of specificity, each sample was tested in triplicates for miR-572 expression using the specific TaqMan^©^ assay. Amplification data were normalized to U6 snRNA expression (Life Technologies, see above for catalog number). Results are shown as Log_10_ relative expression with respect to that of cells transfected with the irrelevant mimic and inhibitor set to 1.

### Microarray analysis

Total RNA extracted from HTLA-230 and SH-SY5Y cells transfected with miR-659-3p and irrelevant mimic and inhibitor from the two experiments were pooled. Two-hundred ng of each of the eight samples were hybridized to Human GE 4x44K v2 Microarray Kit (Agilent Technologies) containing probes for 41,000 human transcripts. One-color microarray-based gene expression analysis using the Low Input Quick Amp Labeling v6.5 protocol (www.agilent.com) was performed. Slides were scanned by Agilent G2505C scanner and images were processed by Feature Extraction software (Agilent Technologies). Microarray data are MIAME compliant and the raw data have been deposited in National Center for Biotechnology Information Gene Expression Omnibus (GEO, www.ncbi.nlm.nih.gov/geo/, accession number GSE65153). Microarray data were analyzed as described in the statistical analysis section, and the probes whose expression was specifically modified by the miR-659-3p mimic and inhibitor were merged to the probes differentially expressed by BM-infiltrating cells and primary tumors ([10], GEO accession number GSE 25623a).

### Gene expression analysis

To evaluate gene expression levels in miR-659-3p transfected cells, 150 ng of total RNA was reverse transcribed as described [8,38], and then amplified in triplicate with the specific TaqMan^©^ human gene expression assay (*CNOT1*: Hs00406740_m1, *GTSE1*: Hs00212681_m1, *KIF4A*: Hs00602211_g1, *AKT3*: Hs00987350_m1, *BCL2*: Hs00608023_m1, *CYR61*: Hs00998500_g1, *HEY1*: Hs01114113_m1, *RNASEL*: Hs00221692_m1, *THBS2*: Hs01568063_m1, ZNF652: Hs00977533_m1), and for *HPRT1* (Hs99999909_m1) as endogenous reference gene (Life Technologies). Results are shown as Log_10_ relative expression with respect to that of cells treated with the irrelevant mimic and inhibitor, set to 1.

To evaluate gene expression levels in BM-infiltrating and primary tumors, total RNA was extracted and reverse transcribed from ten samples per group, as described [8]. Amplification was performed in triplicate using the specific TaqMan^©^ human gene expression assay (see above), and *HPRT1* as endogenous reference gene. Relative expression was calculated as Log_10_ of 2^−ΔCq^, where ΔCq is the difference between the Cq of the target gene and the Cq of the reference *HPRT1* gene [5].

### Statistical analysis

Analysis of miRNA expression Cq values from high-throughput qPCR assays was conducted using the HTqPCR package [39] of Bioconductor [40], which runs on R statistical computing environment (http://www.R-project.org/). To reduce the number of significant miRNAs differentially expressed between BM-infiltrating cells and primary tumors that deserved further studies, two selection criteria were sequentially applied. First, we excluded significant miRNAs unexpressed in some samples of both groups, unless the number of samples in one group was at least twice the number of samples with no expression in the other group. Then, direct inspection of the distribution of expression values among the 12 BM-infiltrating cells and the 12 primary tumors was performed. Only miRNAs whose first quartile of expression levels in one of the two groups was higher than the third quartile in the other group were accepted.

Difference in miRNA expression levels in BM-infiltrating cells and primary tumors evaluated by RT-qPCR in 96 well plates were tested by performing Mann-Whitney rank test, adjusting *p* values to face multiple comparison problems, according to the Benjamini-Hochberg procedure [41].

Differential whole gene expression analysis of cell lines transfected with miR-659-3p and irrelevant mimic and inhibitor was performed using the limma package of Bioconductor [42,43]. Data were preliminarly corrected for background [44] and normalized in order to have similar distributions across the set of arrays. Ranking of genes by their evidence for differential expression was made using the paired moderated t-statistics based on empirical Bayes moderation of the standard errors [42].

Differences in gene expression levels evaluated by RT-qPCR in BM-infiltrating cells and primary tumors were tested by the Mann-Whitney rank test using the Prism software (GraphPad Software Inc., La Jolla, CA, USA).

## SUPPLEMENTARY TABLES












