# Supplementary material for: Deregulation of focal adhesion pathway mediated by miR-659-3p is implicated in bone marrow infiltration of stage M neuroblastoma patients
Source: Oncotarget. 2015 Apr 20;6(15):13295–308. doi: 10.18632/oncotarget.3745 (PMC4537015; doi:10.18632/oncotarget.3745)
Supplement: Supplementary file 1 [file oncotarget-06-13295-s001.pdf]

SUPPLEMENTARY TABLES

Supplementary Table S1: MiRNAs differentially expressed by BM-infiltrating cells and primary tumors

Supplementary Table S2: MiRNAs differentially expressed by BM-infiltrating cells and primary tumors that passed the first selection criterion

mi-RNA[a]	Mann-Whitney test	*P* value	Adjusted *p* value	ΔCq	Fold change
hsa-let-7f-1	12	5.92E-04	6.90E-03	6.7082	0.0096
hsa-miR-10b-5p	70	7.30E-06	5.71E-04	5.7624	0.0184
hsa-miR-128	17	1.65E-03	1.18E-02	4.0350	0.0610
hsa-miR-134	21	3.55E-03	1.93E-02	4.3535	0.0489
hsa-miR-137	1	4.69E-05	1.72E-03	7.5034	0.0055
hsa-miR-140-5p	12	5.92E-04	6.90E-03	6.6971	0.0096
hsa-miR-142-3p	17	1.65E-03	1.18E-02	5.4245	0.0233
hsa-miR-146b-5p	18	2.01E-03	1.36E-02	4.2431	0.0528
hsa-miR-151-3p	120	6.10E-03	2.53E-02	–3.8232	14.1544
hsa-miR-16-5p	2	6.01E-05	1.83E-03	5.9219	0.0165
hsa-miR-191-5p	9	3.08E-04	5.45E-03	3.8467	0.0695
**hsa-miR-211**	135	3.08E-04	5.45E-03	–4.2028	18.4149
hsa-miR-218	18	2.01E-03	1.36E-02	3.9477	0.0648
hsa-miR-218-2	131	7.31E-04	8.01E-03	–6.2973	78.6435
hsa-miR-26a-2	17	1.65E-03	1.18E-02	5.6400	0.0201
hsa-miR-301a-3p	9	3.08E-04	5.45E-03	6.8303	0.0088
hsa-miR-302c	431	3.30E-03	1.88E-02	–7.4755	177.9685
hsa-miR-30c	23	5.11E-03	2.26E-02	3.5418	0.0859
**hsa-miR-323-3p**	22	4.26E-03	2.14E-02	4.1244	0.0573
hsa-miR-324-3p	144	3.66E-05	1.54E-03	–6.7685	109.0272
hsa-miR-335	81	2.06E-05	1.41E-03	4.7424	0.0374
hsa-miR-33b	137	1.96E-04	4.30E-03	–6.0754	67.4329
hsa-miR-342-3p	20	2.95E-03	1.72E-02	3.7523	0.0742
**hsa-miR-361-3p**	68	5.98E-06	5.46E-04	6.4014	0.0118
hsa-miR-361-5p	20	2.95E-03	1.72E-02	2.6877	0.1552
hsa-miR-365a-3p	5	1.23E-04	3.07E-03	6.2956	0.0127
hsa-miR-376c	9	3.08E-04	5.45E-03	6.4238	0.0116
hsa-miR-382	28	1.20E-02	4.39E-02	4.8423	0.0349
hsa-miR-423-5p	23	5.11E-03	2.26E-02	1.9336	0.2618
hsa-miR-449b	11	4.78E-04	6.54E-03	5.3632	0.0243
hsa-miR-450a	7	1.96E-04	4.30E-03	8.4516	0.0029

(*Continued*)

mi-RNA[a]	Mann-Whitney test	*P* value	Adjusted *p* value	ΔCq	Fold change
**hsa-miR-501-3p**	28	1.20E-02	4.39E-02	5.7740	0.0183
hsa-miR-503	0	3.66E-05	1.54E-03	9.0805	0.0018
hsa-miR-515-5p	120	6.10E-03	2.53E-02	–4.0099	16.1102
**hsa-miR-516a-3p**	133	4.78E-04	6.54E-03	–5.9304	60.9863
hsa-miR-517b	124	2.95E-03	1.72E-02	–5.5468	46.7464
hsa-miR-548d-3p	1	4.69E-05	1.72E-03	5.3682	0.0242
hsa-miR-572	128	1.00E-03	9.48E-03	5.4839	0.0223
hsa-miR-576-5p	11	4.78E-04	6.54E-03	5.5557	0.0213
hsa-miR-582-5p	23	5.11E-03	2.26E-02	5.9244	0.0165
hsa-miR-616-5p	54	1.47E-06	1.62E-04	6.4232	0.0117
hsa-miR-628-3p	20	3.44E-08	4.71E-06	8.9097	0.0021
hsa-miR-628-5p	144	3.66E-05	1.54E-03	–7.9952	255.149
hsa-miR-651	23	5.11E-03	2.26E-02	6.0340	0.0153
hsa-miR-656	96	7.82E-05	2.14E-03	6.9874	0.0079
**hsa-miR-659-3p**	144	3.66E-05	1.54E-03	–8.6341	397.2920
hsa-miR-660	15	1.11E-03	9.62E-03	4.7679	0.0367
hsa-miR-7-1	15	1.11E-03	9.62E-03	5.4396	0.0230
**hsa-miR-768-3p**	444	1.34E-03	1.02E-02	–1.9853	3.9595
hsa-miR-873-5p	11	4.78E-04	6.54E-03	6.9894	0.0079
**hsa-miR-892b**	128	1.35E-03	1.02E-02	–3.8607	14.5278
hsa-miR-98-5p	2	6.01E-05	1.83E-03	11.2384	0.0004
RNU6B	1935	9.82E-09	1.79E-06	–2.5656	5.9201

[a]mi-RNAs found differentially expressed also in paired BM-infiltrating cells and primary tumors are indicated in bold.

Supplementary Table S3: MiRNAs expressed at the same level in adrenal gland (cortex + medulla) and NB cell lines a.

Supplementary Table S4: MiRNA differentially expressed between HTLA-230 and SH-SY5Y cell lines a.

Supplementary Table S5: MiRNA differentially expressed between normal adrenal gland (cortex + medulla) and NB cell lines a.
