# Supplementary material for: Deregulation of focal adhesion pathway mediated by miR-659-3p is implicated in bone marrow infiltration of stage M neuroblastoma patients
Source: Oncotarget. 2015 Apr 20;6(15):13295–308. doi: 10.18632/oncotarget.3745 (PMC4537015; doi:10.18632/oncotarget.3745)
Supplement: Supplementary file 2 [file oncotarget-06-13295-s002.pdf]

Row	Col	ControlType	ProbeName	SystematicName	logFC	AveExpr	t	P.Value	adj.P.Val	B
170	82	0	A_23_P340382	NM_001129993	-0,97685026	6,004364129	-7,24157778	0,000458968	0,843980537	-3,992362595
94	69	0	A_24_P111044	ENST00000501068	-1,015618192	6,455326111	-7,070339957	0,000519361	0,843980537	-3,99620824
111	69	0	A_24_P264806	AF090892	-1,014481434	5,697723768	-7,044228806	0,000529359	0,843980537	-3,996815645
105	70	0	A_32_P177741	A_32_P177741	-0,970934258	6,260856464	-6,957047059	0,000564395	0,843980537	-3,998885724
359	9	0	A_23_P503010	NM_153646	1,078913431	5,336911463	6,847058539	0,000612502	0,843980537	-4,001592846
55	73	0	A_23_P154150	NM_005279	-1,304758615	5,118023981	-6,827810438	0,000621401	0,843980537	-4,002077921
118	77	0	A_24_P615181	THC2583059	-1,021861007	6,71125151	-6,796038295	0,00063642	0,843980537	-4,002886183
182	77	0	A_24_P941896	NM_017551	-1,393107718	5,513229923	-6,389014662	0,000871367	0,843980537	-4,014137144
135	79	0	A_24_P922653	NP1166121	-0,988414801	4,947310751	-6,226647222	0,000992131	0,843980537	-4,019136004
116	66	0	A_23_P217712	NM_001669	-0,886715357	6,833891992	-6,009888927	0,001184769	0,843980537	-4,026324047
141	71	0	A_23_P23646	NM_007122	-0,799556601	6,510173203	-5,702858906	0,001536445	0,843980537	-4,037633372
319	31	0	A_24_P662646	NM_001195535	1,3442936	5,914123791	5,682085285	0,001564294	0,843980537	-4,038450785
96	72	0	A_32_P31021	A_32_P31021	-0,876624621	6,330082138	-5,624196056	0,001645018	0,843980537	-4,04076587
118	66	0	A_23_P50974	NM_020760	-0,810067032	6,63992609	-5,620887271	0,001649775	0,843980537	-4,040899873
174	82	0	A_23_P97339	NM_004696	-0,962772983	6,398035363	-5,619274737	0,001652099	0,843980537	-4,040965245
155	76	0	A_23_P211028	NM_004540	-0,785713023	6,044725283	-5,605851588	0,001671591	0,843980537	-4,041511116
120	68	0	A_24_P923514	A_24_P923514	-1,133013888	5,682335206	-5,591411837	0,001692856	0,843980537	-4,042101727
184	84	0	A_23_P62070	NM_001003841	-0,87601335	5,810737354	-5,565884439	0,001731216	0,843980537	-4,043154534
134	65	0	A_23_P697	NM_004980	-0,992220546	5,84932619	-5,543159161	0,001766207	0,843980537	-4,044101219
97	57	0	A_24_P129417	NM_001199	-0,734763887	6,853523607	-5,538477896	0,001773516	0,843980537	-4,044297344
112	74	0	A_24_P407373	ENST00000352367	-1,0445746	6,139056709	-5,493338106	0,001845805	0,843980537	-4,046208298
506	34	0	A_24_P8333	ENST00000515276	0,913938929	5,418766245	5,467091489	0,001889393	0,843980537	-4,04733612
168	82	0	A_24_P503588	AK023380	-0,845104967	6,185647054	-5,457591207	0,001905461	0,843980537	-4,047747416
142	77	0	A_23_P200960	NM_001017922	-1,071256358	5,522290249	-5,436029136	0,001942516	0,843980537	-4,048687015
411	4	0	A_23_P13294	NM_001004459	0,816200951	6,158155674	5,410620291	0,001987248	0,843980537	-4,049805233
202	44	0	A_32_P62026	THC2733296	-0,709172289	6,005370876	-5,279371389	0,002238025	0,843980537	-4,055776597
121	82	0	A_23_P111571	NM_153631	1,117391002	12,10143316	5,232526448	0,002336189	0,843980537	-4,057989896
144	60	0	A_23_P352990	NR_027246	-0,866234574	6,044664923	-5,207969723	0,002389616	0,843980537	-4,059167979
321	5	0	A_23_P402919	NM_152630	0,704072362	5,535866912	5,120562161	0,002591482	0,843980537	-4,063463613
127	81	0	A_23_P42963	NM_139175	-1,059249311	5,335770756	-5,101633616	0,002637731	0,843980537	-4,064415382
231	28	0	A_24_P25285	AK096239	0,965632041	5,741779207	5,097083023	0,00264899	0,843980537	-4,064645361
193	60	0	A_32_P147419	A_32_P147419	-0,774673195	6,166460589	-5,090386177	0,00266566	0,843980537	-4,064984633
457	38	0	A_32_P207726	THC2692269	1,093421475	5,01439276	5,069003224	0,002719696	0,843980537	-4,066074541
107	64	0	A_23_P88576	NM_001080534	-0,71662116	6,269883166	-5,062338549	0,002736794	0,843980537	-4,066416317
170	9	0	A_32_P331916	NR_026743	0,966950327	5,471598904	5,048655058	0,002772285	0,843980537	-4,06712114
146	73	0	A_23_P11841	NM_001684	-0,771059916	6,853174483	-5,046901028	0,002776873	0,843980537	-4,067211792
183	77	0	A_23_P100177	NM_002428	-0,807064462	6,192890003	-5,024569489	0,002836044	0,843980537	-4,068372
184	18	0	A_23_P23037	NM_005267	1,027364319	5,736568205	5,020777413	0,002846235	0,843980537	-4,068570135
136	80	0	A_24_P933448	ENST00000521189	-1,019007034	5,779159711	-4,967157935	0,002994907	0,843980537	-4,071407128
138	79	0	A_23_P346086	NM_001043352	-0,804670797	6,595051832	-4,914492478	0,003149643	0,843980537	-4,074259214
336	14	0	A_24_P931711	A_24_P931711	0,792233314	7,236601798	4,913199318	0,003153556	0,843980537	-4,074330078
194	77	0	A_32_P108870	NM_002677	-0,905070915	5,337293067	-4,904193394	0,00318096	0,843980537	-4,07482471
92	74	0	A_24_P360993	NM_016511	-0,781793167	6,028803515	-4,90153285	0,003189108	0,843980537	-4,07497121
512	33	0	A_32_P159334	NM_002907	0,936802857	5,252054084	4,900677999	0,003191731	0,843980537	-4,075018318
112	20	0	A_23_P50817	NM_020659	0,9575675	6,429658396	4,885478871	0,003238783	0,843980537	-4,075858856
46	74	0	A_24_P255338	NM_004922	-0,843243571	7,38449286	-4,880346399	0,00325485	0,843980537	-4,076143964
186	79	0	A_24_P711290	A_24_P711290	-1,116959997	6,353308443	-4,858010172	0,003325842	0,843980537	-4,077392278
172	57	0	A_23_P257815	NM_005582	-0,713454375	5,237259284	-4,855262441	0,003334697	0,843980537	-4,077546693
503	40	0	A_24_P289178	NM_206967	0,753519617	7,605929845	4,817786756	0,003458194	0,843980537	-4,079671554
95	69	0	A_24_P747419	CV570707	-0,724186591	6,188498624	-4,784730965	0,003571475	0,843980537	-4,081575299
90	84	0	A_23_P114626	NM_000488	-0,96939345	5,729325278	-4,769561039	0,003624876	0,843980537	-4,082458355
216	84	0	A_32_P2333	NM_006713	0,673911207	11,05085018	4,765658944	0,003638759	0,843980537	-4,082686464
201	66	0	A_24_P940375	A_24_P940375	-0,866662349	6,133507087	-4,762178483	0,003651193	0,843980537	-4,082890259
178	79	0	A_24_P156769	NM_005373	-0,705495879	5,800719685	-4,749954254	0,003695251	0,843980537	-4,083608542
342	68	0	A_24_P128524	NM_012405	-0,749175682	5,873345278	-4,746192594	0,00370893	0,843980537	-4,083830361
155	72	0	A_24_P76414	ENST00000310542	-0,77214126	5,745864522	-4,741586737	0,003725758	0,843980537	-4,084102467
192	84	0	A_23_P412508	NM_173806	-0,896248596	5,954906349	-4,728952902	0,003772366	0,843980537	-4,084851724
217	21	0	A_23_P214866	NM_001003699	0,806013889	6,172641164	4,722176737	0,003797638	0,843980537	-4,08525533
164	70	0	A_24_P928371	AF132199	-0,795615881	5,577197686	-4,721627262	0,003799696	0,843980537	-4,085288111
375	8	0	A_24_P602320	THC2584866	1,08612294	6,253521261	4,711173437	0,003839088	0,843980537	-4,085913319
495	34	0	A_24_P473582	ENST00000519893	0,713364696	6,073003164	4,695743508	0,003898085	0,843980537	-4,086841478
167	5	0	A_24_P914759	NM_201382	0,640367961	6,17645596	4,694053147	0,003904611	0,843980537	-4,086943548
183	69	0	A_32_P228759	A_32_P228759	-0,697472233	6,700096728	-4,683401922	0,003946019	0,843980537	-4,087588483
117	69	0	A_32_P111919	BX538090	-0,847375906	6,248280653	-4,678635794	0,00396471	0,843980537	-4,08787807
21	58	0	A_24_P187766	NM_018334	-0,625809552	7,825291049	-4,672178208	0,003990195	0,843980537	-4,088271415
92	60	0	A_23_P74870	NM_025063	-0,715822509	5,470390326	-4,667948517	0,004006989	0,843980537	-4,088529672
110	73	0	A_23_P170409	L11573	-0,891593608	5,537027451	-4,661112242	0,004034302	0,843980537	-4,088948116
112	83	0	A_24_P465772	NR_026825	0,688490805	12,03931708	4,65862536	0,004044291	0,843980537	-4,089100655
141	2	0	A_32_P303449	NM_199339	0,650556324	6,139343261	4,640425504	0,004118256	0,843980537	-4,090222175
293	53	0	A_23_P205632	NM_015163	-0,639631578	5,368142123	-4,610992662	0,004241158	0,843980537	-4,092055394
283	42	0	A_24_P919825	A_24_P919825	0,818138148	5,861028138	4,609429763	0,0042478	0,843980537	-4,092153419
75	77	0	A_23_P155796	NM_000406	-1,27757187	5,63141438	-4,607695529	0,004255184	0,843980537	-4,09226227
145	14	0	A_23_P385263	NM_015557	0,749162298	5,759075341	4,602234922	0,00427853	0,843980537	-4,092605565
370	64	0	A_23_P335981	NM_173860	0,649201242	6,186776063	4,600371455	0,004286531	0,843980537	-4,09272291
95	65	0	A_23_P13007	AF334946	-0,966838796	5,776878532	-4,577551033	0,004385899	0,843980537	-4,094167949
152	12	0	A_24_P54220	NM_000639	1,124879493	5,332335641	4,573026172	0,004405912	0,843980537	-4,094456242
97	70	0	A_24_P935454	AY358106	-0,79829848	6,088923167	-4,567153254	0,004432043	0,843980537	-4,094831301
190	23	0	A_23_P168288	NM_052962	0,643549362	5,195743252	4,560054633	0,004463862	0,843980537	-4,095285964
114	75	0	A_24_P626932	AF007191	-0,92603999	6,469338117	-4,557200434	0,00447673	0,843980537	-4,095469184
86	52	0	A_32_P204376	NM_001012421	-0,877722387	5,356214655	-4,546158721	0,004526907	0,843980537	-4,096180215
127	61	0	A_23_P301414	NM_004449	-0,672366821	5,694075128	-4,539443701	0,004557735	0,843980537	-4,096614365
361	15	0	A_24_P41530	A_24_P41530	0,713418662	8,186161024	4,503918532	0,004724856	0,843980537	-4,098933244
409	35	0	A_23_P90710	NM_001927	0,795934208	6,427121637	4,496470425	0,004760772	0,843980537	-4,099424157
503	46	0	A_24_P97825	NM_015621	0,625545359	5,920265263	4,493392685	0,004775704	0,843980537	-4,099627499
136	79	0	A_24_P300253	NM_014386	-1,059141535	5,663009578	-4,486336064	0,004810141	0,843980537	-4,10009479
124	66	0	A_23_P91727	NM_012407	-0,697163313	7,634662758	-4,48083413	0,004837187	0,843980537	-4,100460167
141	82	0	A_32_P44628	A_32_P44628	-0,834609222	5,252591108	-4,479063494	0,004845927	0,843980537	-4,100577948
302	18	0	A_23_P23564	NM_003557	0,6102537	6,358062095	4,476067037	0,00486076	0,843980537	-4,100777483
168	81	0	A_24_P145163	NM_001204760	-0,922920206	5,544599631	-4,473202651	0,004874987	0,843980537	-4,100968477

127	58	0	A_23_P10518	NM_016521	-0,789118552	6,168585707	-4,460199965	0,004940169	0,843980537	-4,10183861
211	33	0	A_32_P33114	NM_175737	0,65490748	5,458475561	4,459858495	0,004941895	0,843980537	-4,10186153
185	25	0	A_23_P115998	NM_003120	0,624834228	6,351981236	4,45162121	0,004983717	0,843980537	-4,102415513
455	34	0	A_23_P253081	NM_002183	0,70822456	5,602422274	4,438400079	0,005051686	0,843980537	-4,10330902
359	6	0	A_32_P94176	THC2713710	1,072257781	6,542773319	4,433233405	0,005078534	0,843980537	-4,103659655
181	64	0	A_23_P397468	NM_012351	-0,924107993	5,952374412	-4,426827283	0,005112048	0,843980537	-4,104095549
198	28	0	A_32_P188620	THC2679488	0,978453803	5,028062528	4,423578356	0,00512914	0,843980537	-4,104317103
148	81	0	A_32_P120183	ENST00000435531	-0,720929839	6,213412112	-4,405952975	0,005223007	0,843980537	-4,105524754
189	5	0	A_32_P77571	AK024584	1,021305634	5,711519459	4,40341466	0,005236685	0,843980537	-4,105699473
445	8	0	A_23_P64661	NM_032496	-0,638760905	5,901112637	-4,393568761	0,005290128	0,843980537	-4,106379108
150	78	0	A_32_P12140	THC2731029	-0,940951162	6,218629445	-4,389560327	0,005312062	0,843980537	-4,106656674
26	64	0	A_24_P181583	NM_145798	-0,751537367	6,500830178	-4,388759086	0,005316459	0,843980537	-4,106712217
326	11	0	A_32_P87972	A_32_P87972	1,091233649	5,833552135	4,383187053	0,005347151	0,843980537	-4,107099039
144	1	0	A_23_P76460	NM_002469	1,171703674	5,374676949	4,382180836	0,005352714	0,843980537	-4,107168998
132	30	0	A_24_P941359	NM_014722	0,764599933	5,35731977	4,381407291	0,005356996	0,843980537	-4,107222801
528	44	0	A_24_P764595	A_24_P764595	0,61114152	8,399087154	4,374872552	0,005393323	0,843980537	-4,10767808
135	66	0	A_24_P199251	NM_005447	-1,068595878	5,679839579	-4,370014802	0,005420508	0,843980537	-4,108017403
411	81	0	A_24_P341985	NM_031938	-0,938606285	6,043341046	-4,352838545	0,005517886	0,843980537	-4,109223247
92	84	0	A_24_P118512	A_24_P118512	-0,754998834	5,900244222	-4,346183101	0,00555615	0,843980537	-4,109693035
76	66	0	A_32_P117590	ENST00000570581	-0,77624805	6,391138902	-4,333264282	0,005631288	0,843980537	-4,110609024
459	85	0	A_23_P408376	NM_025015	0,780222614	9,204488967	4,320973505	0,005703844	0,843980537	-4,111485521
496	74	0	A_32_P424932	NM_014496	-0,579807587	7,309244058	-4,314968702	0,005739677	0,843980537	-4,111915539
275	25	0	A_23_P368681	NM_015660	0,621437955	5,766878663	4,312308196	0,005755635	0,843980537	-4,112106442
252	1	0	A_24_P58221	THC2630554	0,859612278	5,832654625	4,295516925	0,005857518	0,843980537	-4,113316671
3	76	0	A_23_P125771	NM_005334	-0,870874795	6,383874647	-4,293944991	0,005867161	0,843980537	-4,113430446
145	65	0	A_24_P491397	NM_001010978	-0,846645035	6,469463648	-4,289065574	0,005897206	0,843980537	-4,113784133
129	82	0	A_32_P86067	NM_003412	-1,339463992	6,134719098	-4,287047365	0,005909684	0,843980537	-4,113930655
483	15	0	A_24_P940831	AK024424	0,799195634	4,965758248	4,280595564	0,005949775	0,843980537	-4,114399968
144	65	0	A_23_P432506	ENST00000475015	-0,677024833	7,080089069	-4,279819959	0,005954615	0,843980537	-4,11445648
513	47	0	A_24_P144054	A_24_P144054	0,624193918	5,694450777	4,276385217	0,005976103	0,843980537	-4,114706984
502	10	0	A_24_P401381	A_24_P401381	-0,588732538	6,260950286	-4,272363287	0,006001378	0,843980537	-4,115000814
111	68	0	A_23_P309207	NM_032679	-0,642100924	5,883685396	-4,271414191	0,006007359	0,843980537	-4,115070231
76	61	0	A_24_P936988	AY358219	-0,593807769	5,973571552	-4,270531405	0,006012929	0,843980537	-4,115134826
146	85	0	A_23_P133408	NM_000758	-1,298905031	6,674409095	-4,268270356	0,006027222	0,843980537	-4,115300389
158	74	0	A_23_P11644	NM_006945	-0,967512645	6,462492053	-4,266186151	0,006040431	0,843980537	-4,115453155
263	65	0	A_32_P43349	NR_034095	-0,881630454	5,653322216	-4,264590265	0,006050568	0,843980537	-4,115570227
106	61	0	A_24_P917759	ENST00000565498	-0,725420659	6,140617551	-4,26021531	0,006078455	0,843980537	-4,115891609
153	65	0	A_23_P39881	NR_023391	-0,811019109	5,633568181	-4,255196067	0,006110628	0,843980537	-4,116261116
156	73	0	A_32_P28264	THC2663978	-0,609501018	6,28990711	-4,252803471	0,006126033	0,843980537	-4,116437554
194	6	0	A_32_P885445	A_32_P885445	0,818415754	6,547727411	4,249716818	0,006145971	0,843980537	-4,116665461
7	71	0	A_32_P95960	NM_001004419	-0,658356413	5,260321007	-4,242353567	0,006193831	0,843980537	-4,117210442
86	55	0	A_24_P623734	NM_001144994	-0,576738751	7,100392966	-4,241682181	0,006198215	0,843980537	-4,117260226
476	45	0	A_23_P355295	NM_001460	0,847196903	5,486833252	4,23659031	0,006231585	0,843980537	-4,11763829
371	48	0	A_32_P187704	ENST00000521574	0,880568872	6,05116284	4,228354931	0,006285988	0,843980537	-4,118251631
487	63	0	A_24_P117147	NM_013289	0,782562727	6,457777729	4,228022115	0,006288198	0,843980537	-4,118276466
512	44	0	A_23_P109614	NM_182935	0,594919269	5,790346841	4,225176658	0,006307128	0,843980537	-4,118488958
179	65	0	A_24_P219686	NM_006788	-0,61220124	6,393164281	-4,217359083	0,006359467	0,843980537	-4,11907419
173	82	0	A_24_P932934	S45570	-0,910670214	6,334227031	-4,211621791	0,006398192	0,843980537	-4,11950503
254	13	0	A_32_P898690	ENST00000333666	0,790620691	5,486939334	4,202731421	0,006458727	0,843980537	-4,1201749
173	69	0	A_24_P102125	AF285120	-0,711847665	6,604440314	-4,196306216	0,006502881	0,843980537	-4,120660734
27	72	0	A_23_P350698	NM_153228	-0,686723999	5,828497037	-4,190604976	0,006542346	0,843980537	-4,121093032
221	1	0	A_23_P116902	NM_021071	0,751638738	5,953080482	4,184887013	0,006582198	0,843980537	-4,121527739
104	47	0	A_24_P643587	NM_024095	-0,769832701	6,546228609	-4,180379315	0,00661381	0,843980537	-4,121871244
414	39	0	A_23_P100963	NM_182538	0,811725865	6,280385237	4,17567511	0,006646982	0,843980537	-4,122230486
346	79	0	A_24_P488202	ENST00000359491	-0,875040437	5,786199753	-4,17028158	0,006685248	0,843980537	-4,122643327
148	31	0	A_32_P199049	ENST00000575722	1,035136581	5,29268277	4,16347871	0,006733868	0,843980537	-4,123165511
176	14	0	A_32_P402331	NM_145652	0,893074763	6,067480388	4,16316273	0,006736136	0,843980537	-4,123189806
133	72	0	A_24_P111242	NM_148893	-1,018936701	6,397142128	-4,161050689	0,006751318	0,843980537	-4,123352282
192	70	0	A_24_P373877	NM_003180	-0,711436258	7,588745438	-4,154948189	0,006795402	0,843980537	-4,123822628
104	83	0	A_24_P457164	A_24_P457164	-0,904102999	5,32062196	-4,153497072	0,006805932	0,843980537	-4,123934667
168	78	0	A_23_P149545	NM_003528	-1,092152489	5,751016884	-4,149129171	0,00683774	0,843980537	-4,124272359
162	77	0	A_23_P138480	NM_024886	-0,834833824	7,526177423	-4,146129289	0,006859685	0,843980537	-4,124504682
195	66	0	A_23_P251075	NM_005491	-0,73008984	6,16982799	-4,142272688	0,006888013	0,843980537	-4,124803825
161	1	0	A_32_P63993	ENST00000602702	0,641253515	7,224814226	4,141565477	0,006893222	0,843980537	-4,124858739
306	15	0	A_32_P83098	NM_000689	0,901922774	6,837762673	4,118701482	0,007064051	0,843980537	-4,126643781
116	85	0	A_24_P532727	TCONS_00006171	-1,042611934	5,955730016	-4,11683755	0,007078187	0,843980537	-4,126790135
457	81	0	A_32_P936189	ENST00000566100	-1,015426851	5,798533887	-4,09533398	0,007243604	0,843980537	-4,128487719
415	85	0	A_24_P144377	NM_014015	0,96784455	9,702368912	4,083447396	0,007336913	0,843980537	-4,129433365
142	81	0	A_23_P304446	NM_006213	-0,708527169	6,294676879	-4,078553549	0,007375724	0,843980537	-4,129824212
291	5	0	A_24_P926979	NM_182925	0,840413013	6,319305614	4,074652829	0,007406824	0,843980537	-4,130136378
437	5	0	A_32_P219554	DB352127	0,808575099	5,93193394	4,071281518	0,007433823	0,843980537	-4,13040663
149	82	0	A_24_P73264	NM_030640	-0,841598956	6,388933714	-4,066384291	0,007473241	0,843980537	-4,130799955
262	15	0	A_23_P309720	NM_000815	0,610013946	6,743257743	4,066077769	0,007475716	0,843980537	-4,130824604
65	55	0	A_23_P400887	NM_012279	-0,537376747	6,604547067	-4,061073629	0,007516253	0,843980537	-4,131227495
191	62	0	A_23_P145844	NM_000245	-0,704161434	5,77979259	-4,05946179	0,007529363	0,843980537	-4,131357466
217	11	0	A_24_P838797	NR_103794	1,036940863	5,587949248	4,051806923	0,007591977	0,843980537	-4,131976042
96	54	0	A_24_P126406	NM_173628	-0,581107177	5,992347809	-4,044986935	0,007648257	0,843980537	-4,132529003
485	79	0	A_24_P919899	ENST00000371937	-1,091051599	6,849151863	-4,044540551	0,007651957	0,843980537	-4,132565256
58	77	0	A_24_P38363	NM_002467	-0,659683666	5,495053235	-4,042239247	0,007671065	0,843980537	-4,132752278
55	50	0	A_24_P208992	NM_001012614	-0,603876859	8,030043086	-4,042072645	0,00767245	0,843980537	-4,132765825
91	65	0	A_32_P109604	NR_046228	-0,70015923	6,616050104	-4,039430912	0,007694455	0,843980537	-4,132980775
164	84	0	A_23_P169460	NM_014907	-0,595096867	6,284597009	-4,036047727	0,007722741	0,843980537	-4,13325644
192	68	0	A_32_P486443	ENST00000219478	-0,648777205	7,028802576	-4,033676434	0,007742636	0,843980537	-4,133449912
63	53	0	A_23_P54612	NM_178452	-0,79908601	5,865220929	-4,030980697	0,007765323	0,843980537	-4,133670114
236	2	0	A_23_P301021	NM_004319	0,751445859	6,814477253	4,030519675	0,007769211	0,843980537	-4,133707801
77	84	0	A_23_P342612	NM_001194	0,928165786	13,29680649	4,0297585	0,007775634	0,843980537	-4,133770041
18	77	0	A_24_P930237	A_24_P930237	-0,782664091	5,681117052	-4,029393064	0,00777872	0,843980537	-4,133799929
476	40	0	A_23_P12549	NM_019043	0,689969438	5,155370891	4,027506027	0,007794677	0,843980537	-4,13395435
406	7	0	A_24_P93397	A_24_P93397	0,848692573	5,538645911	4,024924034	0,007816571	0,843980537	-4,134165859
527	34	0	A_23_P412214	NM_015085	-0,556058271	6,572101216	-4,01626853	0,007890472	0,843980537	-4,134876742
339	10	0	A_24_P40306	NM_000185	0,901828479	5,620503867	4,012629463	0,007921778	0,843980537	-4,135176475
202	80	0	A_24_P927902	A_24_P927902	-0,86228751	6,243528772	-4,006011612	0,007979068	0,843980537	-4,135722853
104	51	0	A_23_P116868	NM_017435	-0,779929577	6,089261666	-4,006003729	0,007979137	0,843980537	-4,135723505
306	73	0	A_24_P639671	NR_045196	0,539088774	7,415226479	4,005791491	0,007980982	0,843980537	-4,135741055

137	72	0	A_24_P318160	NM_014903	-1,241166165	5,5513217	-4,005226545	0,007985896	0,843980537	-4,13578778
204	77	0	A_24_P109191	A_24_P109191	-0,718129624	6,399338185	-4,004930506	0,007988472	0,843980537	-4,13581227
175	84	0	A_23_P68740	NM_000383	-0,641539163	6,78395429	-4,002257224	0,008011778	0,843980537	-4,136033566
86	58	0	A_23_P90577	NM_022437	-0,731552169	5,280210439	-4,000656376	0,008025771	0,843980537	-4,136166217
355	38	0	A_24_P205406	A_24_P205406	0,60383525	5,056843731	4,000598237	0,00802628	0,843980537	-4,136171036
159	79	0	A_24_P282060	AK093006	-0,585304066	7,458228654	-3,996854605	0,008059113	0,843980537	-4,136481638
394	26	0	A_24_P114142	NM_005076	0,574622361	7,427609199	3,993192876	0,008091374	0,843980537	-4,136785966
162	16	0	A_23_P161507	NM_004923	0,908239471	8,514546257	3,990450678	0,00811563	0,843980537	-4,137014211
150	11	0	A_23_P306610	NM_001040105	0,587223503	5,943442657	3,986724803	0,008148717	0,843980537	-4,137324797
328	3	0	A_23_P32583	A_23_P32583	0,85485805	6,172520137	3,981280746	0,008197337	0,843980537	-4,137779575
132	16	0	A_32_P214020	ENST00000519741	0,621086185	5,43263589	3,979180362	0,008216182	0,843980537	-4,137955342
128	71	0	A_23_P39315	NM_021187	-0,953066488	5,720032422	-3,977245228	0,008233588	0,843980537	-4,138117432
128	62	0	A_24_P937691	NM_015065	-0,606949777	6,385448376	-3,976803782	0,008237565	0,843980537	-4,138154428
108	65	0	A_32_P114739	DB518505	-0,764394168	5,710439291	-3,974558783	0,008257821	0,843980537	-4,138342694
92	64	0	A_24_P928119	A_24_P928119	-0,624851984	6,866629747	-3,972269612	0,008278534	0,843980537	-4,138534866
139	8	0	A_23_P300100	ENST00000375105	0,527692411	6,455452592	3,967012765	0,00832632	0,843980537	-4,138976943
10	73	0	A_23_P12755	NM_032211	-0,591208151	6,677215423	-3,966255404	0,008333231	0,843980537	-4,139040723
342	42	0	A_23_P45409	NM_080632	0,592977602	8,296416061	3,966035578	0,008335238	0,843980537	-4,139059239
100	78	0	A_23_P325593	NM_152448	-0,854030548	5,643897552	-3,96590286	0,00833645	0,843980537	-4,139070419
117	76	0	A_23_P101683	NM_001828	-1,060910065	6,13327408	-3,965164623	0,008343194	0,843980537	-4,13913262
25	59	0	A_24_P304311	NM_004254	-0,694372271	5,705411199	-3,961819821	0,008373831	0,843980537	-4,139414707
143	60	0	A_23_P255714	NM_025103	-0,749463571	7,128961187	-3,951505714	0,008469105	0,843980537	-4,140287322
287	16	0	A_23_P391946	AK055900	0,567882384	5,801260002	3,945453616	0,008525578	0,843980537	-4,140801303
528	22	0	A_24_P920148	A_24_P920148	0,758562125	5,117361204	3,945031044	0,008529537	0,843980537	-4,140837244
163	1	0	A_24_P66691	ENST00000534945	0,702177052	7,207156926	3,942006365	0,008557935	0,843980537	-4,141094711
103	72	0	A_32_P188850	ENST00000602554	-0,6758666	6,494352297	-3,941216127	0,008565371	0,843980537	-4,141162037
197	77	0	A_32_P233527	ENST00000468356	-0,581186734	6,927054988	-3,938943885	0,008586796	0,843980537	-4,141355764
531	75	0	A_23_P91764	NM_052945	-0,576444024	7,272120153	-3,937552557	0,008599944	0,843980537	-4,141474488
259	72	0	A_23_P9565	NM_031296	-0,885420798	6,084751523	-3,933145989	0,008641736	0,843980537	-4,14185101
153	60	0	A_24_P636179	A_24_P636179	-0,789162046	7,066078026	-3,932153945	0,008651176	0,843980537	-4,141935883
327	66	0	A_32_P193091	THC2727226	0,531711908	6,158662558	3,93111232	0,0086611	0,843980537	-4,142025039
184	20	0	A_23_P133457	NM_024337	0,562702164	6,426036044	3,928074034	0,008690122	0,843980537	-4,142285343
226	32	0	A_24_P854556	NR_034007	0,529764432	5,210107364	3,924746423	0,008722033	0,843980537	-4,142570857
281	20	0	A_24_P202632	NM_017509	0,515835654	6,572215724	3,923204627	0,008736863	0,843980537	-4,142703296
162	71	0	A_23_P50039	NM_005913	-0,991679641	6,613284282	-3,922074045	0,008747755	0,843980537	-4,142800472
115	31	0	A_24_P843020	THC2767512	0,517504788	5,645199375	3,912485072	0,008840759	0,843980537	-4,143626721
100	19	0	A_24_P892494	NR_015450	0,561281395	5,986983744	3,90303293	0,008933527	0,843980537	-4,144444793
105	50	0	A_32_P211752	XR_242321	-0,609663391	6,170284124	-3,898944779	0,008973989	0,843980537	-4,144799733
21	48	0	A_23_P18518	NM_152402	-0,615122894	6,817974494	-3,891984306	0,009043354	0,843980537	-4,145405608
292	23	0	A_24_P529786	AK091744	0,795571136	7,758478213	3,888210691	0,009081213	0,843980537	-4,145734904
45	50	0	A_32_P198791	THC2557762	-0,6627231	6,040956217	-3,884549781	0,009118111	0,843980537	-4,146054918
99	81	0	A_23_P215718	NM_014690	-0,698027301	6,451272181	-3,884498181	0,009118633	0,843980537	-4,146059432
111	61	0	A_24_P921167	A_24_P921167	-0,680703505	5,663292023	-3,882160085	0,009142288	0,843980537	-4,146264104
181	69	0	A_23_P145786	NM_032951	-1,209661304	6,200748883	-3,879136732	0,009172979	0,843980537	-4,146529093
167	80	0	A_32_P17182	A_32_P17182	-0,957252557	6,105881754	-3,877642813	0,009188187	0,843980537	-4,146660168
187	15	0	A_23_P56347	NM_021016	0,735994036	5,802250448	3,869943854	0,009267013	0,843980537	-4,147337116
459	44	0	A_23_P81017	NM_020337	0,548382224	6,630611595	3,864645646	0,0093217	0,843980537	-4,147804384
397	16	0	A_23_P364544	NM_175874	0,665111585	7,580860187	3,859349618	0,009376728	0,843980537	-4,148272612
280	4	0	A_23_P25246	NM_000706	0,600011221	5,594697207	3,859138416	0,00937893	0,843980537	-4,148291308
158	55	0	A_32_P180199	A_32_P180199	-0,616361048	6,848671844	-3,853424127	0,00943873	0,843980537	-4,148797862
226	70	0	A_32_P14386	CU675288	-0,522537646	6,640012292	-3,85308882	0,009442252	0,843980537	-4,148827627
85	71	0	A_24_P100266	NM_003932	-0,508654592	9,507834089	-3,848699147	0,0094885	0,843980537	-4,149217735
208	66	0	A_24_P572974	THC2708549	-0,957196386	6,742685794	-3,844943859	0,009528267	0,843980537	-4,149552098
236	18	0	A_32_P116816	BG205430	0,8592137	5,708622918	3,842303891	0,009556335	0,843980537	-4,149787506
366	17	0	A_24_P915294	A_24_P915294	0,577473802	5,398931207	3,841331611	0,009566696	0,843980537	-4,149874277
146	84	0	A_24_P242646	NM_004079	-1,087321183	4,960044747	-3,84014289	0,00957938	0,843980537	-4,149980419
93	57	0	A_23_P158041	NM_001170	-0,834513334	6,142360213	-3,840056741	0,0095803	0,843980537	-4,149988113
500	71	0	A_23_P415015	NM_022374	-0,776910799	6,085162855	-3,839645917	0,009584688	0,843980537	-4,150024811
139	65	0	A_23_P252936	NM_015266	-0,523767707	5,932132523	-3,837513242	0,009607507	0,843980537	-4,15021543
156	68	0	A_23_P375354	NM_005422	-0,818193784	6,261731354	-3,837331103	0,009609458	0,843980537	-4,150231719
192	83	0	A_23_P63209	NM_181755	-1,031837156	6,333762213	-3,836008382	0,009623645	0,843980537	-4,150350049
513	44	0	A_23_P255345	NM_004666	0,938038998	5,365318239	3,834363632	0,009641317	0,843980537	-4,15049729
150	66	0	A_32_P150269	A_32_P150269	-0,771461391	5,87842037	-3,830534877	0,009682599	0,843980537	-4,150840484
38	2	0	A_23_P417918	NM_006211	0,805971155	6,095515732	3,822828009	0,009766297	0,843980537	-4,151533153
507	41	0	A_24_P621434	THC2512545	0,584873977	6,781288722	3,817596516	0,009823576	0,843980537	-4,152004762
109	72	0	A_23_P207180	NM_022581	-0,614215577	5,99406608	-3,817030115	0,0098298	0,843980537	-4,152055891
57	53	0	A_24_P426	NM_022776	-0,653342707	7,058462227	-3,816830837	0,009831991	0,843980537	-4,152073883
191	84	0	A_23_P411941	NM_172000	-0,682494461	5,405493188	-3,811122726	0,009894979	0,843980537	-4,152589955
283	2	0	A_23_P312132	NM_000887	0,841099695	6,248658928	3,810023726	0,009907158	0,843980537	-4,152689474
65	53	0	A_23_P77552	NM_198490	-0,584499941	6,288701674	-3,803292429	0,009982124	0,843980537	-4,153300131
230	30	0	A_23_P110903	NM_080743	0,607991436	8,288602137	3,802594899	0,009989929	0,843980537	-4,15336352
104	50	0	A_23_P113793	NM_024508	-0,540771268	5,888296156	-3,802592392	0,009989957	0,843980537	-4,153363747
72	85	0	A_24_P635355	A_24_P635355	-0,513842155	6,511958606	-3,800843344	0,010009557	0,843980537	-4,153522785
343	6	0	A_23_P152428	NM_052858	0,58745063	5,956267248	3,792327676	0,010105605	0,843980537	-4,154298952
176	23	0	A_23_P321354	NM_144649	0,773776937	5,798899913	3,789600956	0,010136578	0,843980537	-4,154548132
462	38	0	A_24_P925134	A_24_P925134	0,987382014	5,617353509	3,78668294	0,010169842	0,843980537	-4,154815145
263	74	0	A_23_P204208	NM_002262	-0,701623165	5,956310954	-3,785521432	0,010183117	0,843980537	-4,15492153
193	66	0	A_32_P157775	THC2564488	-0,544026095	6,424068051	-3,782871933	0,01021347	0,843980537	-4,155164418
344	55	0	A_24_P928931	AK022288	0,503927132	5,735427585	3,782703954	0,010215398	0,843980537	-4,155179828
238	56	0	A_23_P62741	NM_022159	-0,530165049	5,762977928	-3,779343909	0,010254046	0,843980537	-4,15548831
385	41	0	A_24_P242319	A_24_P242319	0,840327379	5,517655482	3,778994358	0,010258077	0,843980537	-4,155520429
130	76	0	A_24_P313109	NM_016617	-0,598147327	6,600856985	-3,773714648	0,010319164	0,843980537	-4,15600621
235	34	0	A_23_P28258	NM_017431	0,5523592	5,933481757	3,77293102	0,010328266	0,843980537	-4,156078412
248	1	0	A_23_P1523	NM_014578	0,726510744	6,662139206	3,768400349	0,010381065	0,843980537	-4,156496381
227	25	0	A_23_P250444	NM_000166	0,564124635	5,504551243	3,76651571	0,010403117	0,843980537	-4,156670505
119	77	0	A_24_P922624	A_24_P922624	-0,887033513	6,107935174	-3,763612259	0,010437193	0,843980537	-4,156939057
7	70	0	A_24_P59111	BC035912	-0,589923919	6,445008105	-3,761897957	0,010457372	0,843980537	-4,157097791
359	5	0	A_23_P7744	NM_031952	0,686523568	6,027816382	3,761722522	0,010459439	0,843980537	-4,157114042
364	47	0	A_24_P196318	NM_001001290	0,702911747	6,020178802	3,755824608	0,010529212	0,843980537	-4,157661167
412	85	0	A_23_P34968	NM_001204856	0,537328334	12,29600761	3,752334443	0,010570747	0,843980537	-4,157985644
398	69	0	A_23_P327698	NM_001007527	-0,625291369	6,511628137	-3,75130088	0,010583083	0,843980537	-4,158081834
183	66	0	A_24_P96403	NM_001001890	-0,608296161	5,84582636	-3,749779641	0,010601268	0,843980537	-4,158223495
63	60	0	A_23_P383422	NM_139239	-0,788984531	6,00600713	-3,749239199	0,010607736	0,843980537	-4,158273847
435	38	0	A_24_P614579	BC000206	0,60249755	9,639703489	3,748661282	0,010614659	0,843980537	-4,158327704

69	71	0	A_32_P50846	THC2751803	-0,808535406	5,590977103	-3,74700222	0,010634559	0,843980537	-4,158482395
131	1	0	A_32_P70818	NM_006194	0,619468111	6,943970631	3,746227464	0,010643867	0,843980537	-4,158554674
152	79	0	A_32_P222030	A_32_P222030	-0,565368118	6,723428579	-3,745457043	0,010653132	0,843980537	-4,158626575
444	36	0	A_24_P676999	A_24_P676999	0,596509486	5,745745043	3,741947688	0,010695447	0,843980537	-4,158954417
99	52	0	A_23_P316012	NM_020663	-0,667237437	6,281967062	-3,741660814	0,010698915	0,843980537	-4,158981241
118	57	0	A_23_P134566	NM_001005328	-0,748427219	5,148688073	-3,74137682	0,010702349	0,843980537	-4,159007798
140	69	0	A_32_P164225	BF308099	-0,588449749	6,916055156	-3,739856458	0,010720753	0,843980537	-4,159150034
179	25	0	A_24_P920355	ENST00000341893	0,923486549	5,551937763	3,73868535	0,010734953	0,843980537	-4,159259665
2	24	0	A_32_P76091	NM_002156	1,192085808	8,724504663	3,737204287	0,010752943	0,843980537	-4,159398397
130	82	0	A_24_P638294	NM_001029	0,676025965	11,89898287	3,73661252	0,01076014	0,843980537	-4,159453855
500	82	0	A_32_P151454	A_32_P151454	-0,642119465	5,762229466	-3,73445942	0,010786372	0,843980537	-4,159655764
158	67	0	A_24_P378331	NM_170589	-0,722007435	6,143872245	-3,733731149	0,010795261	0,843980537	-4,159724104
148	70	0	A_24_P302506	ENST00000369864	-0,512359733	6,73181761	-3,732875086	0,010805721	0,843980537	-4,159804465
38	36	0	A_32_P119233	A_32_P119233	-0,615512492	6,633155833	-3,73262413	0,010808789	0,843980537	-4,15982803
48	61	0	A_23_P8139	NM_003427	-0,876447702	7,09222162	-3,7311787	0,010826481	0,843980537	-4,159963806
362	6	0	A_24_P925505	S67044	0,511847353	5,659682178	3,728770277	0,010856031	0,843980537	-4,160190244
251	84	0	A_32_P136776	ENST00000502125	-1,134574857	6,146341384	-3,72790153	0,010866712	0,843980537	-4,160271985
317	31	0	A_32_P30834	NR_038377	0,727041252	5,948131158	3,725662291	0,010894299	0,843980537	-4,16048283
16	77	0	A_24_P931583	A_24_P931583	-0,531321286	6,859944404	-3,720875063	0,010953538	0,843980537	-4,160934331
204	21	0	A_24_P698698	A_24_P698698	0,895979722	6,60861379	3,717182143	0,010999483	0,843980537	-4,161283312
462	44	0	A_23_P319895	NM_015048	-0,578536664	6,367183944	-3,715013402	0,011026566	0,843980537	-4,161488538
191	22	0	A_24_P23740	A_24_P23740	0,639303343	6,015099872	3,714098051	0,011038019	0,843980537	-4,161575219
267	1	0	A_32_P155364	NM_000971	1,176736651	10,4307589	3,7107141	0,011080476	0,843980537	-4,161895992
41	77	0	A_23_P323685	NM_003543	0,515697269	8,829983633	3,710519004	0,011082929	0,843980537	-4,161914501
514	81	0	A_24_P510081	NR_038273	-0,569561685	5,467003453	-3,708715096	0,011105642	0,843980537	-4,16208572
502	35	0	A_24_P937656	AL833848	0,560364959	6,189515206	3,706858374	0,011129075	0,843980537	-4,162262102
126	78	0	A_32_P165933	NM_203424	-0,754854151	6,197200651	-3,700789202	0,011206058	0,843980537	-4,162839718
294	3	0	A_24_P264549	A_24_P264549	0,572137887	6,712826911	3,699232639	0,011225898	0,843980537	-4,162988123
262	11	0	A_23_P161439	NM_006829	0,687505913	6,819937504	3,697569807	0,011247135	0,843980537	-4,163146779
105	23	0	A_24_P524588	A_24_P524588	0,663971312	6,296462099	3,697346528	0,01124999	0,843980537	-4,163168092
148	65	0	A_24_P141019	NM_018412	-0,511615899	6,778163771	-3,692677639	0,01130988	0,843980537	-4,163614269
245	14	0	A_24_P246591	A_24_P246591	1,055781022	6,596384648	3,684277982	0,011418526	0,843980537	-4,164419421
127	21	0	A_23_P204087	NM_016817	0,525145591	5,759079183	3,678929128	0,01148832	0,843980537	-4,164933783
291	22	0	A_23_P71213	NM_130768	0,549452958	6,184329207	3,678714851	0,011491126	0,843980537	-4,164954415
141	72	0	A_24_P111106	NM_000800	-0,82687743	6,499837818	-3,677360905	0,011508874	0,843980537	-4,165084831
430	42	0	A_32_P206136	A_32_P206136	0,499556205	5,79027591	3,674095778	0,011551799	0,843980537	-4,165399676
129	6	0	A_23_P328145	NM_173526	0,717427126	6,600182474	3,668608786	0,011624339	0,843980537	-4,165929848
499	52	0	A_24_P326491	NM_173576	0,783029801	6,104769562	3,668271351	0,011628816	0,843980537	-4,165962497
148	77	0	A_32_P211494	A_32_P211494	-0,530374538	6,504704952	-3,666869508	0,011647439	0,843980537	-4,166098187
94	59	0	A_23_P352266	NM_000633	0,624108622	8,380893857	3,666771535	0,011648742	0,843980537	-4,166107673
78	69	0	A_24_P565080	NR_038360	-1,167871697	6,046514075	-3,664020224	0,011685393	0,843980537	-4,166374253
145	71	0	A_24_P914599	Z25431	-0,833180805	6,132253519	-3,66338268	0,011693904	0,843980537	-4,166436075
145	81	0	A_24_P54174	NM_001066	-0,773171198	6,353620767	-3,663160031	0,011696878	0,843980537	-4,166457669
157	67	0	A_23_P27353	NM_007163	-0,527503424	6,056908378	-3,660429463	0,01173342	0,843980537	-4,166722684
146	76	0	A_24_P884376	TCONS_l2_00025849	-0,828888123	6,434316494	-3,657426601	0,011773754	0,843980537	-4,167014516
129	58	0	A_32_P134825	THC2528990	-0,486968087	6,704130858	-3,655561022	0,011798891	0,843980537	-4,167196027
88	67	0	A_23_P427278	ENST00000521751	-0,712743886	6,571380338	-3,655140715	0,011804562	0,843980537	-4,167236942
384	12	0	A_23_P253689	AF118069	0,658162683	6,023714582	3,65511577	0,011804899	0,843980537	-4,167239371
374	20	0	A_23_P2258	NM_201435	0,707378465	6,103587294	3,653943698	0,011820731	0,843980537	-4,167353512
100	14	0	A_23_P45592	NR_001533	0,501930977	6,063857988	3,651110439	0,0118591	0,843980537	-4,167629683
255	31	0	A_24_P912041	A_24_P912041	-0,659607178	6,226063032	-3,649867742	0,011875973	0,843980537	-4,16775093
18	81	0	A_32_P150928	NM_012262	0,640957089	12,94223741	3,648243744	0,011898063	0,843980537	-4,167909485
88	66	0	A_32_P151152	THC2593596	-0,788717594	7,075649154	-3,647580237	0,011907102	0,843980537	-4,1679743
171	75	0	A_23_P56913	NM_001029881	-0,591219776	6,148846572	-3,647208667	0,011912167	0,843980537	-4,168010605
168	80	0	A_24_P624522	A_24_P624522	-0,840396599	6,205546787	-3,64597433	0,01192901	0,843980537	-4,168131255
223	25	0	A_32_P501260	ENST00000438457	0,711315757	6,0219386	3,645555059	0,011934738	0,843980537	-4,168172253
57	1	0	A_23_P166674	NM_000866	0,709641479	6,834791212	3,644964595	0,011942809	0,843980537	-4,168230003
226	34	0	A_24_P357688	NM_015384	0,512363619	8,544257835	3,644804579	0,011944997	0,843980537	-4,168245656
117	82	0	A_23_P397248	NM_006536	-0,658067016	5,313148678	-3,644627563	0,011947418	0,843980537	-4,168262974
157	75	0	A_23_P88554	A_23_P88554	-0,622299511	6,509943014	-3,639980678	0,012011177	0,843980537	-4,168718091
182	67	0	A_32_P202886	THC2735952	-0,587380018	6,340015778	-3,63866738	0,012029265	0,843980537	-4,168846895
108	28	0	A_23_P12435	NM_032103	0,537388278	6,206423723	3,638075902	0,012037422	0,843980537	-4,16890493
164	83	0	A_23_P303286	NM_001122965	-0,725640224	5,723291154	-3,634923072	0,012081004	0,843980537	-4,169214556
31	63	0	A_23_P7866	NM_153838	-0,685692565	5,646321984	-3,634667548	0,012084544	0,843980537	-4,169239669
465	6	0	A_23_P19816	NM_030936	-0,597568667	5,791943268	-3,632480236	0,012114894	0,843980537	-4,169454769
116	76	0	A_23_P329254	NR_026903	-0,857018914	5,328661312	-3,627494584	0,012184389	0,843980537	-4,169945875
215	19	0	A_24_P32085	NM_024761	0,643517281	8,308779538	3,621582254	0,012267381	0,843980537	-4,170529743
115	77	0	A_23_P157963	NM_017738	-0,884439504	6,651224622	-3,620417515	0,012283805	0,843980537	-4,170644956
359	1	0	A_23_P217168	NM_024689	0,847651958	7,058423489	3,620347898	0,012284787	0,843980537	-4,170651844
184	82	0	A_23_P306867	NM_173199	-0,474337167	5,880894922	-3,615237433	0,012357147	0,843980537	-4,171158113
97	77	0	A_23_P135787	NM_001256487	-0,78880818	7,86910028	-3,612321836	0,012398643	0,843980537	-4,171447488
225	84	0	A_23_P203698	ENST00000526712	-0,831216676	5,706956185	-3,610473974	0,012425023	0,843980537	-4,171631092
405	38	0	A_23_P350782	ENST00000390265	0,556202359	6,117738151	3,608699241	0,012450418	0,843980537	-4,17180758
373	13	0	A_23_P415820	AB023136	0,6697916	5,792128522	3,605789282	0,012492183	0,843980537	-4,172097275
151	83	0	A_23_P212854	NM_002100	-0,699185655	5,197145417	-3,601447822	0,012554784	0,843980537	-4,172530211
216	28	0	A_24_P367369	A_24_P367369	0,641315912	8,007836903	3,601268874	0,012557372	0,843980537	-4,172548075
104	66	0	A_24_P913855	A_24_P913855	-0,722389568	5,909505466	-3,601115371	0,012559593	0,843980537	-4,1725634
344	27	0	A_24_P936312	BX648310	0,481820626	6,306128053	3,600918718	0,012562438	0,843980537	-4,172583034
64	11	0	A_23_P76350	NM_002075	-0,807137221	8,105787161	-3,600696557	0,012565653	0,843980537	-4,172605217
56	72	0	A_24_P733553	ENST00000531155	-0,886845721	6,009933508	-3,599927551	0,012576789	0,843980537	-4,172682022
124	85	0	A_24_P911906	NM_001040429	-0,73889325	6,166798619	-3,596935208	0,012620226	0,843980537	-4,172981144
251	6	0	A_23_P363174	NM_003511	0,542331788	7,974289817	3,596602549	0,012625065	0,843980537	-4,173014423
127	54	0	A_23_P385826	ENST00000529348	-0,759402412	5,77942918	-3,595154756	0,012646151	0,843980537	-4,173159321
431	34	0	A_23_P384532	NM_145020	0,691751961	6,942318892	3,594574792	0,012654608	0,843980537	-4,173217392
149	11	0	A_32_P520	A_32_P520	0,882464457	5,123970339	3,59358018	0,012669127	0,843980537	-4,173317017
484	27	0	A_23_P438	NM_017773	0,554463937	5,687081982	3,593414933	0,012671541	0,843980537	-4,173333574
359	82	0	A_23_P57337	A_23_P57337	-0,60766193	6,246170422	-3,591173073	0,012704343	0,843980537	-4,173558317
151	84	0	A_23_P68155	NM_022168	-0,619809143	5,997274924	-3,589466409	0,012729377	0,843980537	-4,173729565
330	22	0	A_24_P464273	A_24_P464273	0,936299949	5,516390118	3,585468079	0,012788243	0,843980537	-4,174131294
228	31	0	A_24_P37253	NM_194317	0,801411095	8,002545736	3,585362057	0,012789808	0,843980537	-4,174141957
494	33	0	A_32_P6139	NM_001104629	0,801879909	5,660864732	3,583857213	0,012812045	0,843980537	-4,174293356
73	32	0	A_23_P391711	NM_000134	0,668362682	5,084807559	3,577315252	0,012909218	0,843980537	-4,174952764
108	62	0	A_24_P410117	A_24_P410117	-0,73697225	6,346227579	-3,575047394	0,012943096	0,843980537	-4,175181826
108	80	0	A_24_P610961	NP110384	-1,242223137	6,502065921	-3,568216392	0,013045742	0,843980537	-4,175873244

105	62	0	A_23_P74145	NM_001778	-0,556800562	6,175791156	-3,567440663	0,013057456	0,843980537	-4,175951901
138	65	0	A_32_P11096	NM_203303	-0,802818816	6,5631952	-3,567148584	0,013061869	0,843980537	-4,175981524
110	75	0	A_24_P934487	NM_006949	-1,054844319	6,146861716	-3,566313541	0,013074497	0,843980537	-4,176066238
332	39	0	A_24_P135933	TCONS_l2_00009281	0,602635427	6,642096513	3,563725762	0,013113715	0,843980537	-4,176328975
222	35	0	A_24_P610405	BF570136	0,570163803	5,782673184	3,563251878	0,013120911	0,843980537	-4,176377122
171	17	0	A_24_P806076	A_24_P806076	0,653249095	5,727753744	3,562820927	0,013127459	0,843980537	-4,176420917
10	39	0	A_23_P159622	NM_000718	-0,605598541	7,023640117	-3,561112099	0,01315346	0,843980537	-4,17659466
205	12	0	A_23_P79587	NM_001632	0,575158744	7,320404585	3,560050367	0,013169643	0,843980537	-4,176702681
47	48	0	A_23_P139635	NM_001917	-0,534302779	6,216371571	-3,558339858	0,013195762	0,843980537	-4,176876819
158	65	0	A_32_P203280	TCONS_00001493	-0,587592933	5,32068696	-3,557877969	0,013202825	0,843980537	-4,176923866
457	82	0	A_32_P46544	A_32_P46544	-0,674396084	5,900210907	-3,555154498	0,013244556	0,843980537	-4,177201476
173	81	0	A_23_P53370	NM_014470	-0,601215272	6,593142964	-3,552432065	0,013286419	0,843980537	-4,177479331
94	71	0	A_32_P80338	A_32_P80338	-0,660904182	6,621586335	-3,550592555	0,013314789	0,843980537	-4,177667274
71	51	0	A_24_P151582	NM_003216	-0,542300044	6,619505895	-3,548043702	0,01335421	0,843980537	-4,177927956
128	70	0	A_23_P62133	NM_000252	-0,862701852	6,352703796	-3,547082841	0,013369104	0,843980537	-4,178026308
333	25	0	A_23_P102331	NM_002976	0,658145758	5,282483495	3,544881841	0,013403293	0,843980537	-4,178251763
255	55	0	A_24_P288848	NM_176819	-0,608141067	6,257502635	-3,537686242	0,013515745	0,843980537	-4,178990444
22	66	0	A_23_P90014	NM_001080452	-0,630653897	8,083896217	-3,537546952	0,013517933	0,843980537	-4,179004767
330	31	0	A_24_P228700	ENST00000490103	0,699879294	5,554352308	3,536508967	0,013534244	0,843980537	-4,179111535
111	71	0	A_32_P865343	AL359654	-0,735257508	6,227126095	-3,536157635	0,01353977	0,843980537	-4,179147685
504	67	0	A_24_P944427	NM_001080517	-0,612938996	5,967868763	-3,536150388	0,013539884	0,843980537	-4,179148431
156	72	0	A_23_P412577	NM_173505	-0,860239838	6,521025739	-3,531671272	0,013610555	0,843980537	-4,179609824
429	39	0	A_23_P18053	NR_024615	0,613352067	6,726615984	3,52920271	0,01364968	0,843980537	-4,179864522
331	7	0	A_32_P142149	BC007784	0,475190565	8,799440404	3,529200382	0,013649717	0,843980537	-4,179864762
39	70	0	A_23_P158297	NM_197975	-0,756763752	5,459052034	-3,525880578	0,013702531	0,843980537	-4,18020775
180	5	0	A_23_P253495	NM_000847	0,85091556	6,303726356	3,523221611	0,013744997	0,843980537	-4,180482844
61	58	0	A_32_P84049	THC2624002	-1,00860885	6,387435214	-3,522293863	0,013759848	0,843980537	-4,180578909
83	44	0	A_24_P213594	ENST00000502505	-0,590415163	5,944766282	-3,518815675	0,013815686	0,843980537	-4,180939429
136	9	0	A_24_P537149	A_24_P537149	0,544897848	6,257382907	3,518432109	0,013821859	0,843980537	-4,180979222
230	9	0	A_24_P740620	ENST00000428155	0,53064893	6,481970019	3,514857729	0,013879533	0,843980537	-4,181350387
285	13	0	A_24_P72012	NM_031438	0,698451618	5,708242187	3,511411286	0,013935397	0,843980537	-4,181708851
225	8	0	A_24_P892402	ENST00000606641	0,711694764	7,630128005	3,508114702	0,013989067	0,843980537	-4,182052266
82	48	0	A_23_P201837	NM_001531	-0,607037767	5,987999821	-3,50741873	0,014000427	0,843980537	-4,182124835
98	80	0	A_24_P794189	A_24_P794189	-1,002033974	5,937903576	-3,502498504	0,014081032	0,843980537	-4,182638536
199	59	0	A_23_P204581	NM_003330	-0,5849636	8,437689591	-3,502211652	0,014085747	0,843980537	-4,182668521
337	8	0	A_24_P475014	A_24_P475014	0,920684342	5,604333372	3,502071785	0,014088047	0,843980537	-4,182683143
329	73	0	A_24_P349145	NM_001085447	0,504544104	6,457978258	3,499641704	0,014128071	0,843980537	-4,182937342
150	19	0	A_24_P355816	NM_018370	0,961031495	7,853079827	3,498763586	0,014142564	0,843980537	-4,183029269
144	62	0	A_24_P98047	NM_018593	-0,561621635	6,253641753	-3,495879769	0,01419028	0,843980537	-4,183331427
199	84	0	A_23_P8497	NM_000823	-1,340995429	6,685022624	-3,494994441	0,014204964	0,843980537	-4,18342427
77	79	0	A_32_P40433	THC2657737	-1,387413631	5,135774078	-3,493642451	0,014227422	0,843980537	-4,183566126
114	7	0	A_24_P264293	A_24_P264293	0,565489053	6,13804163	3,492631875	0,014244234	0,843980537	-4,183672217
246	28	0	A_24_P152775	A_24_P152775	0,656078745	7,094158464	3,490510165	0,014279604	0,843980537	-4,183895118
492	56	0	A_23_P311358	NM_003575	-0,641643713	6,241706519	-3,490170625	0,014285273	0,843980537	-4,18393081
110	64	0	A_23_P119735	NM_014898	-0,836618925	5,404045889	-3,488262535	0,014317179	0,843980537	-4,184131488
118	8	0	A_24_P392110	NM_182707	0,55776187	5,860325506	3,486899515	0,014340019	0,843980537	-4,184274949
318	5	0	A_24_P341482	M62303	0,796803395	5,889124756	3,484301314	0,01438367	0,843980537	-4,184548668
102	71	0	A_23_P428973	ENST00000354500	-0,839972576	6,08989297	-3,480635335	0,014445512	0,843980537	-4,184935438
140	16	0	A_23_P34031	NM_001079538	0,620649591	6,490568451	3,47856687	0,014480536	0,843980537	-4,185153957
445	23	0	A_24_P940738	A_24_P940738	0,667132674	6,294510955	3,478416702	0,014483083	0,843980537	-4,18516983
277	48	0	A_23_P130130	NM_181844	0,630031619	6,726362112	3,476663505	0,014512848	0,843980537	-4,18535522
121	81	0	A_23_P117992	NM_014861	-0,889162655	5,891452194	-3,472722072	0,014580015	0,843980537	-4,185772556
525	44	0	A_23_P97841	NM_030569	0,711534671	5,658868772	3,469300229	0,014638607	0,843980537	-4,186135495
163	79	0	A_24_P307744	AK093664	-0,834362249	5,444902579	-3,468872696	0,014645946	0,843980537	-4,186180882
23	48	0	A_23_P201059	NM_020131	-0,506144995	7,756404677	-3,468588261	0,014650831	0,843980537	-4,186211082
389	5	0	A_24_P23546	NM_198956	0,777748648	5,475820576	3,467453986	0,01467033	0,843980537	-4,186331557
439	78	0	A_32_P186678	A_32_P186678	-0,784593055	8,200963574	-3,466894039	0,014679966	0,843980537	-4,186391053
170	64	0	A_23_P323749	NM_025049	-0,568003913	7,492964793	-3,463643072	0,014736051	0,843980537	-4,186736789
166	71	0	A_23_P104275	NM_213606	-0,706294327	5,533905103	-3,461119123	0,014779759	0,843980537	-4,187005567
182	27	0	A_23_P116523	ENST00000330381	0,49567802	6,343302685	3,458212721	0,014830268	0,843980537	-4,187315463
136	4	0	A_24_P354954	NM_138771	0,493021024	7,322246148	3,456329715	0,014863095	0,843980537	-4,187516462
140	71	0	A_23_P50517	NM_001277075	-0,807583774	5,475123769	-3,455997641	0,014868893	0,843980537	-4,187551927
188	1	0	A_24_P841662	AK093643	0,528268556	7,165630637	3,447838337	0,015012135	0,843980537	-4,18842505
150	80	0	A_24_P153643	NM_004947	-0,648294541	5,86477411	-3,445616366	0,015051408	0,843980537	-4,188663395
362	5	0	A_32_P447001	NM_001198784	0,504800388	7,019413897	3,445047432	0,015061483	0,843980537	-4,188724462
415	14	0	A_32_P178945	NM_018566	0,455836983	10,06153332	3,442134391	0,015113183	0,843980537	-4,189037391
399	57	0	A_24_P348791	A_24_P348791	0,824075967	5,876329296	3,439644428	0,015157531	0,843980537	-4,189305207
287	83	0	A_23_P250136	NM_175768	-0,487942189	6,31376212	-3,438014955	0,015186632	0,843980537	-4,189480637
198	67	0	A_23_P412603	NM_144773	-0,525094818	6,075378637	-3,437561544	0,01519474	0,843980537	-4,189529476
273	50	0	A_23_P10980	NM_015236	0,749412727	6,851590248	3,437496839	0,015195898	0,843980537	-4,189536446
266	23	0	A_32_P177124	AA854066	0,687031652	5,247719199	3,43465886	0,015246763	0,843980537	-4,189842376
151	85	0	A_24_P333234	AF274942	-0,874812861	6,200915451	-3,434540866	0,015248882	0,843980537	-4,189855104
229	13	0	A_32_P220912	A_32_P220912	0,55930138	6,681383373	3,43322088	0,015272609	0,843980537	-4,189997541
292	84	0	A_24_P889237	AK057637	-1,040924404	5,846600732	-3,430636406	0,015319184	0,843980537	-4,19027668
304	13	0	A_32_P178365	THC2559669	0,744469862	5,601564105	3,430449056	0,015322566	0,843980537	-4,190296928
173	74	0	A_24_P247324	A_24_P247324	-1,054451655	6,808225262	-3,430190078	0,015327243	0,843980537	-4,19032492
99	79	0	A_32_P18617	BX099457	-0,762996955	6,033178038	-3,426305268	0,01539759	0,843980537	-4,190745219
165	65	0	A_24_P264004	A_24_P264004	-0,656430712	6,920348825	-3,425540655	0,015411478	0,843980537	-4,190828032
166	85	0	A_23_P393144	NM_153368	-0,882522116	6,469182626	-3,423670783	0,0154455	0,843980537	-4,191030675
119	76	0	A_23_P103256	NM_021023	-0,840116873	6,124564288	-3,422461678	0,015467544	0,843980537	-4,191161803
437	45	0	A_23_P321452	NM_173791	0,581296032	8,898825738	3,420936418	0,015495401	0,843980537	-4,191327323
333	80	0	A_23_P278039	NM_006571	0,499373329	8,232111174	3,420306252	0,015506927	0,843980537	-4,191395743
265	1	0	A_24_P780709	A_24_P780709	0,593355863	12,87372521	3,416968146	0,015568141	0,843980537	-4,191758505
204	81	0	A_24_P645900	AK023895	-0,827887917	6,49390723	-3,416870995	0,015569926	0,843980537	-4,191769071
414	54	0	A_23_P319512	NM_003597	0,75250602	7,111335764	3,410488327	0,015687735	0,843980537	-4,192464292
161	66	0	A_24_P102249	AK094289	-0,627144284	6,277712059	-3,409899909	0,015698645	0,843980537	-4,192528487
82	46	0	A_24_P404868	NM_001024452	0,459466849	5,618199849	3,409291844	0,015709929	0,843980537	-4,192594845
49	64	0	A_24_P928467	A_24_P928467	-1,071043057	5,836537023	-3,408628056	0,015722257	0,843980537	-4,192667305
262	8	0	A_23_P215382	A_23_P215382	0,686555875	5,91358653	3,40686712	0,015755014	0,843980537	-4,19285964
418	46	0	A_23_P24948	NM_005472	0,961994645	5,477093689	3,403999046	0,015808528	0,843980537	-4,193173234
168	67	0	A_23_P503064	NM_004795	-0,521003625	6,262241865	-3,401856826	0,01584863	0,843980537	-4,193407736
422	64	0	A_23_P213745	NM_004887	0,8119121	7,104900797	3,401355835	0,015858025	0,843980537	-4,193462612
144	85	0	A_24_P265177	ENST00000497658	-1,526185932	6,026380846	-3,400816675	0,015868142	0,843980537	-4,193521682
269	28	0	A_24_P112447	NM_020354	-0,497196785	6,531588445	-3,398549565	0,015910764	0,843980537	-4,193770228

47	38	0	A_24_P941751	A_24_P941751	-0,748794759	6,001495419	-3,398406335	0,015913461	0,843980537	-4,193785939
307	5	0	A_32_P45152	ENST00000449362	0,65065437	6,356932571	3,397671349	0,015927309	0,843980537	-4,193866578
425	39	0	A_32_P254762	NR_001559	0,563486147	5,574450487	3,396562633	0,015948223	0,843980537	-4,193988273
14	70	0	A_24_P923723	AK128263	-0,628251731	6,187250268	-3,393898992	0,015998593	0,843980537	-4,194280894
276	76	0	A_32_P124238	THC2530072	0,586047011	6,743015557	3,393593966	0,016004373	0,843980537	-4,194314426
193	83	0	A_32_P45866	A_32_P45866	-0,627272379	6,343106403	-3,391462708	0,016044819	0,843980537	-4,194548853
330	67	0	A_23_P416774	NM_016929	0,925793686	6,116427539	3,391184427	0,016050109	0,843980537	-4,19457948
532	70	0	A_24_P246406	NM_001252271	-0,669246153	6,695034066	-3,3887276	0,016096892	0,843980537	-4,19485004
259	8	0	A_24_P201353	NM_144591	0,620556434	9,037767143	3,386306878	0,016143136	0,843980537	-4,195116925
294	25	0	A_24_P41276	NM_153366	0,476811894	6,279599071	3,384719683	0,016173537	0,843980537	-4,195292076
517	33	0	A_23_P340698	NM_002426	0,762829643	5,461584369	3,384420114	0,016179282	0,843980537	-4,195325149
62	72	0	A_32_P137812	THC2669156	-1,304303079	6,053263239	-3,382969623	0,01620713	0,843980537	-4,195485349
98	83	0	A_23_P140405	NM_005197	0,492653979	12,62613886	3,379768887	0,016268772	0,843980537	-4,195839236
433	46	0	A_23_P102320	NM_138285	0,469850931	10,92472282	3,376026316	0,016341179	0,843980537	-4,196253695
224	30	0	A_24_P278375	A_24_P278375	0,536745864	5,994087166	3,375863388	0,016344339	0,843980537	-4,196271754
209	24	0	A_23_P130056	NM_020426	0,779257039	6,249472138	3,375043608	0,01636025	0,843980537	-4,196362641
146	20	0	A_23_P159775	NM_004961	0,511076262	7,345751616	3,374335848	0,016374001	0,843980537	-4,196441136
258	49	0	A_23_P310	NM_023009	-0,741089887	12,28526095	-3,372809129	0,016403707	0,843980537	-4,196610546
240	39	0	A_32_P148824	NM_017847	0,480297777	8,977818497	3,372543168	0,016408888	0,843980537	-4,19664007
155	6	0	A_23_P87556	NM_153634	0,819985415	5,699686325	3,369848721	0,01646148	0,843980537	-4,196939384
162	48	0	A_23_P47426	NM_014384	-0,456567169	7,3546159	-3,369802565	0,016462382	0,843980537	-4,196944514
88	59	0	A_23_P216679	NM_033331	-0,63781413	6,202077796	-3,367443518	0,016508584	0,843980537	-4,197206882
72	57	0	A_23_P60599	NM_001072	-0,745626599	5,809110079	-3,366318696	0,016530664	0,843980537	-4,197332083
190	64	0	A_23_P34093	NM_000402	-0,536898425	8,949075222	-3,366281312	0,016531398	0,843980537	-4,197336245
441	49	0	A_23_P399897	NM_178554	0,521614053	6,137210073	3,365367435	0,016549363	0,843980537	-4,197438016
177	83	0	A_23_P10753	ENST00000572499	-0,662657203	6,26593333	-3,364691283	0,016562668	0,843980537	-4,197513341
47	51	0	A_23_P136786	NM_032336	-0,53535835	6,457681055	-3,364482703	0,016566774	0,843980537	-4,197536582
158	81	0	A_24_P676216	A_24_P676216	-0,563339379	6,383193821	-3,361755515	0,016620575	0,843980537	-4,197840666
13	72	0	A_23_P418597	NM_033396	-0,665170811	7,662405821	-3,359505071	0,016665117	0,843980537	-4,198091881
470	40	0	A_32_P55135	NM_005635	0,505934	5,562607323	3,357453874	0,01670583	0,843980537	-4,198321082
95	58	0	A_24_P346967	THC2627335	-0,519388062	6,337126044	-3,357445167	0,016706003	0,843980537	-4,198322055
76	68	0	A_24_P165949	NM_021101	-0,788828767	6,465331962	-3,356821698	0,0167184	0,843980537	-4,198391765
239	1	0	A_24_P155761	NM_006738	0,68705362	8,506105363	3,356050636	0,016733746	0,843980537	-4,198478005
312	15	0	A_23_P10232	NM_017935	0,527954495	5,373997815	3,353823198	0,016778165	0,843980537	-4,198727306
499	68	0	A_24_P94034	NM_015276	-0,663764869	7,751371117	-3,352133987	0,016811938	0,843980537	-4,198916539
23	82	0	A_23_P106024	NM_002226	-0,776539017	6,706909304	-3,351988641	0,016814847	0,843980537	-4,198932828
235	69	0	A_32_P10831	A_32_P10831	-0,522311909	5,847008097	-3,350669898	0,016841271	0,843980537	-4,199080672
102	73	0	A_24_P100830	NM_001278412	-0,547953256	6,810888537	-3,349951633	0,016855682	0,843980537	-4,199161234
48	77	0	A_23_P37685	NM_024600	-0,605855067	6,560681779	-3,349251033	0,016869752	0,843980537	-4,199239841
218	31	0	A_32_P178017	A_32_P178017	0,594065526	5,868633856	3,348490489	0,016885041	0,843980537	-4,199325202
162	82	0	A_23_P71226	NM_022444	-0,551828113	6,195678284	-3,347615792	0,016902643	0,843980537	-4,199423413
103	68	0	A_32_P221966	NR_027082	-0,455192217	6,082006515	-3,346336597	0,016928422	0,843980537	-4,199567111
61	48	0	A_24_P342236	BC034740	-0,704513251	5,711885302	-3,346142972	0,016932328	0,843980537	-4,19958887
178	69	0	A_23_P65615	NM_015859	-0,510857093	6,468456089	-3,345449068	0,016946334	0,843980537	-4,199666862
155	1	0	A_24_P413941	NM_153689	0,495582523	8,410286128	3,344755336	0,016960349	0,843980537	-4,19974486
103	77	0	A_23_P410998	NM_002868	-0,575124537	8,59359917	-3,342797517	0,016999971	0,843980537	-4,199965117
368	5	0	A_32_P72758	NM_018349	0,610450146	5,679340048	3,339312786	0,017070749	0,843980537	-4,200357646
515	40	0	A_32_P126023	THC2559380	0,733947302	5,57632499	3,337789143	0,017101798	0,843980537	-4,200529471
266	26	0	A_32_P173922	A_32_P173922	0,54449832	8,474408479	3,330251183	0,017256332	0,843980537	-4,201381327
217	14	0	A_23_P51767	NM_001765	0,883257152	6,053537823	3,32926718	0,017276619	0,843980537	-4,201492747
499	81	0	A_23_P117125	NM_000966	-0,687565992	7,07946876	-3,328330721	0,017295951	0,843980537	-4,20159883
124	5	0	A_23_P22800	NM_001077697	0,662870948	5,678080174	3,32754399	0,01731221	0,843980537	-4,201687987
159	28	0	A_24_P177844	ENST00000604066	0,53763545	6,316824144	3,325703946	0,017350303	0,843980537	-4,201896639
138	60	0	A_24_P241672	NM_080283	-0,574404171	5,887441256	-3,325370175	0,017357223	0,843980537	-4,201934506
421	83	0	A_24_P142118	NM_003246	-0,585561615	6,725602697	-3,321893367	0,017429487	0,843980537	-4,202329302
370	7	0	A_23_P393051	NM_152365	0,49117654	5,857627544	3,321727215	0,017432949	0,843980537	-4,202348185
98	73	0	A_32_P62211	XR_241982	-0,935116046	6,772484682	-3,320760234	0,017453111	0,843980537	-4,202458107
113	79	0	A_23_P412427	NR_046316	-0,690878403	7,098562795	-3,318998853	0,017489903	0,843980537	-4,202658461
40	75	0	A_23_P130648	NM_005716	-0,726648446	9,076815318	-3,317546868	0,017520297	0,843980537	-4,202823743
86	53	0	A_24_P516594	ENST00000451601	-0,540545408	5,246861845	-3,315975142	0,017553263	0,843980537	-4,203002781
38	63	0	A_32_P26536	A_32_P26536	-0,767411353	5,916737838	-3,315872434	0,01755542	0,843980537	-4,203014485
246	53	0	A_23_P31921	NM_000050	-0,467829915	8,983062825	-3,315759799	0,017557785	0,843980537	-4,203027321
256	78	0	A_24_P348885	NM_182580	-0,531039635	6,664723169	-3,315481412	0,017563633	0,843980537	-4,203059049
419	43	0	A_23_P96611	NM_016383	0,689515909	5,89189275	3,314598384	0,017582197	0,843980537	-4,203159716
384	65	0	A_23_P20392	NM_015310	0,513467091	9,694450348	3,312109269	0,017634642	0,843980537	-4,2034437
120	85	0	A_32_P23960	A_32_P23960	-0,869768593	7,284330974	-3,308534384	0,017710267	0,843980537	-4,20385213
206	48	0	A_32_P130217	A_32_P130217	-0,446686604	6,729566252	-3,306610193	0,017751121	0,843980537	-4,204072247
217	72	0	A_32_P154342	NM_180991	-0,559739062	6,385141837	-3,306590036	0,01775155	0,843980537	-4,204074554
481	72	0	A_32_P193262	BG115931	-0,86565787	5,787016525	-3,306366598	0,0177563	0,843980537	-4,204100127
109	69	0	A_23_P407132	NM_032531	-0,831762231	5,702811295	-3,306345492	0,017756749	0,843980537	-4,204102543
289	42	0	A_23_P200524	NM_001005353	-0,433016368	9,178587471	-3,303745091	0,017812146	0,843980537	-4,204400361
484	36	0	A_32_P62796	TCONS_00009108	0,827010932	5,91125033	3,299763941	0,017897328	0,843980537	-4,204857005
365	3	0	A_24_P246573	NM_015209	0,558973005	8,04217293	3,298662933	0,017920966	0,843980537	-4,20498344
530	35	0	A_23_P218405	NM_004489	-0,450631639	8,405548621	-3,296013972	0,017977977	0,843980537	-4,205287897
137	80	0	A_23_P357760	ENST00000481340	-0,649197729	6,58821658	-3,295680258	0,017985174	0,843980537	-4,205326279
247	80	0	A_24_P918739	BC028378	-0,589857621	6,948517792	-3,295576199	0,017987418	0,843980537	-4,205338249
93	67	0	A_23_P422212	NM_173508	-0,798360333	6,398071072	-3,293125345	0,018040376	0,843980537	-4,205620324
485	61	0	A_23_P51019	NM_021007	0,538569177	6,991495344	3,293037846	0,01804227	0,843980537	-4,205630401
195	33	0	A_23_P314835	NM_178456	0,690825164	5,823766674	3,290246232	0,018102808	0,843980537	-4,205952096
176	38	0	A_24_P822427	A_24_P822427	0,597271339	5,567973783	3,289568635	0,018117536	0,843980537	-4,206030242
485	58	0	A_23_P325411	NM_001003699	0,528449521	8,439436049	3,288077111	0,018150002	0,843980537	-4,206202343
201	81	0	A_32_P174805	A_32_P174805	-0,734451706	6,973951029	-3,286859097	0,018176562	0,843980537	-4,206342972
127	4	0	A_24_P293530	NM_178033	0,743638062	5,413127291	3,286761201	0,018178699	0,843980537	-4,206354279
160	83	0	A_23_P250629	NM_004159	-0,832969487	6,378659728	-3,285488121	0,018206509	0,843980537	-4,206501356
168	8	0	A_24_P340941	NR_036507	0,704059033	6,160684518	3,282232352	0,018277847	0,843980537	-4,206877884
306	34	0	A_24_P222324	ENST00000439423	0,756789246	5,456903561	3,281945243	0,018284153	0,843980537	-4,206911115
121	53	0	A_24_P932719	THC2599662	-0,556104548	5,764592946	-3,280640915	0,01831283	0,843980537	-4,207062138
485	85	0	A_24_P154948	NM_002047	0,588671201	8,011590284	3,278257423	0,018365362	0,843980537	-4,207338347
386	41	0	A_24_P854643	AL833114	0,457313025	6,059979474	3,277296751	0,018386582	0,843980537	-4,20744976
134	52	0	A_32_P421527	ENST00000419926	-0,586227946	5,246746566	-3,276886351	0,018395656	0,843980537	-4,20749737
175	37	0	A_23_P58251	NM_001014448	0,524836039	6,642236518	3,276552659	0,018403037	0,843980537	-4,207536088
128	8	0	A_32_P141086	A_32_P141086	0,485972019	5,9922299	3,275821324	0,018419226	0,843980537	-4,207620965
434	55	0	A_32_P228124	NM_002072	0,674470332	9,642046883	3,273773878	0,018464632	0,843980537	-4,20785874
22	54	0	A_23_P118536	NM_018042	-0,553907532	5,354795026	-3,273765629	0,018464815	0,843980537	-4,207859698

92	72	0	A_24_P339416	NM_014960	-0,750306406	6,924021959	-3,273601169	0,018468468	0,843980537	-4,207878807
169	69	0	A_23_P115444	NM_005092	-0,831151208	6,039936579	-3,272146464	0,018500811	0,843980537	-4,208047894
158	18	0	A_23_P102017	NM_020711	0,630779214	5,892581123	3,271036462	0,018525533	0,843980537	-4,208176991
498	41	0	A_32_P115606	XM_005265254	0,439972392	8,06688837	3,270477109	0,018538004	0,843980537	-4,208242071
149	15	0	A_24_P7322	A_24_P7322	0,499666533	5,850233345	3,268736337	0,018576877	0,843980537	-4,208444713
212	22	0	A_24_P342055	NM_001286563	0,736076165	5,995526654	3,268620838	0,018579459	0,843980537	-4,208458164
132	77	0	A_23_P153941	NM_024766	-0,596493267	6,657971426	-3,268360629	0,018585278	0,843980537	-4,20848847
58	34	0	A_23_P395418	NM_032097	-0,609386412	6,109094702	-3,267717319	0,018599674	0,843980537	-4,208563411
222	81	0	A_23_P127840	NM_013249	-0,725399379	6,873172046	-3,267400904	0,018606759	0,843980537	-4,20860028
153	16	0	A_24_P279704	NM_080600	0,943899368	6,19427485	3,265590108	0,018647363	0,843980537	-4,208811375
168	79	0	A_24_P221615	ENST00000560724	-0,897120822	5,98577732	-3,264260248	0,018677245	0,843980537	-4,208966516
202	13	0	A_23_P397937	NM_001258275	0,687690048	5,728810477	3,263309333	0,018698645	0,843980537	-4,209077508
259	69	0	A_23_P436117	NM_018200	-0,530138588	8,784077203	-3,262759742	0,018711026	0,843980537	-4,209141678
267	75	0	A_23_P113789	NM_006677	-0,661716822	7,410455184	-3,262612828	0,018714337	0,843980537	-4,209158835
431	43	0	A_32_P114675	BX105849	0,873509909	6,340820005	3,262603565	0,018714546	0,843980537	-4,209159917
142	12	0	A_23_P330561	NM_174918	0,627888545	5,592919033	3,262586865	0,018714922	0,843980537	-4,209161867
456	50	0	A_32_P183451	A_32_P183451	0,520881524	6,218756098	3,262543116	0,018715908	0,843980537	-4,209166976
524	75	0	A_24_P417706	NM_001142935	-0,591423027	7,032370611	-3,261981372	0,018728576	0,843980537	-4,209232589
90	79	0	A_32_P87013	NM_000584	-0,922016238	5,411613043	-3,260655015	0,018758523	0,843980537	-4,209387579
446	50	0	A_32_P5568	XR_249807	0,619313115	6,027113863	3,260276673	0,018767075	0,843980537	-4,209431806
293	31	0	A_23_P168532	NM_003088	-0,438334314	10,12828434	-3,259721936	0,018779622	0,843980537	-4,209496668
27	70	0	A_24_P136683	NR_026551	-0,782029228	8,086527115	-3,258871115	0,018798884	0,843980537	-4,209596182
135	65	0	A_24_P256434	AK097130	-0,870379609	6,14053257	-3,257455634	0,018830978	0,843980537	-4,209761825
181	78	0	A_23_P77228	NM_022769	-0,619309758	8,809001197	-3,256508316	0,018852491	0,843980537	-4,209872742
526	14	0	A_23_P212665	NM_005688	-0,675681975	6,109453817	-3,254349042	0,018901628	0,843980537	-4,210125743
180	46	0	A_24_P929835	A_24_P929835	0,501017472	5,187884726	3,252972867	0,018933019	0,843980537	-4,210287119
22	68	0	A_24_P730679	A_24_P730679	-0,652473351	5,460003667	-3,252104691	0,018952852	0,843980537	-4,210388978
30	66	0	A_23_P105794	NM_033255	-0,670053222	6,475815885	-3,251449717	0,01896783	0,843980537	-4,21046585
407	16	0	A_32_P113462	A_32_P113462	-0,483149269	6,01054598	-3,251249926	0,018972401	0,843980537	-4,210489303
23	73	0	A_32_P62469	A_32_P62469	-0,703374387	6,371546801	-3,25076811	0,01898343	0,843980537	-4,210545871
470	70	0	A_23_P209464	NM_024969	-1,068054515	6,77063299	-3,250009119	0,019000819	0,843980537	-4,210635008
397	49	0	A_23_P52951	A_23_P52951	0,444998989	6,286505084	3,249969831	0,019001719	0,843980537	-4,210639623
39	85	0	A_23_P95879	NM_000999	0,676478032	15,19092822	3,249925938	0,019002725	0,843980537	-4,210644778
389	2	0	A_23_P873	BC031655	0,509932273	9,979296363	3,249788389	0,019005879	0,843980537	-4,210660936
339	7	0	A_24_P340036	NM_194463	0,6059083	6,304683196	3,2479377	0,019048364	0,843980537	-4,210878434
104	84	0	A_24_P214858	NM_024807	-0,720821523	5,759385729	-3,247530605	0,019057724	0,843980537	-4,210926302
13	58	0	A_24_P150791	NM_020655	-0,642281539	7,021912611	-3,243813336	0,019143424	0,843980537	-4,211363805
195	76	0	A_32_P77139	THC2731337	-0,439422718	6,618346026	-3,243554564	0,019149405	0,843980537	-4,211394289
32	51	0	A_32_P228356	A_32_P228356	-0,610896343	5,690904504	-3,24328288	0,019155688	0,843980537	-4,211426298
98	66	0	A_32_P217428	A_32_P217428	-1,132889878	7,02122598	-3,243063797	0,019160755	0,843980537	-4,211452112
130	64	0	A_23_P42063	AF090903	-0,941525913	6,152331719	-3,242225979	0,019180149	0,843980537	-4,211550856
129	65	0	A_23_P165280	NM_002695	-0,465299279	9,694800516	-3,242053658	0,019184141	0,843980537	-4,21157117
294	12	0	A_23_P9415	NM_002197	0,749448253	8,493699201	3,241152571	0,019205028	0,843980537	-4,211677421
181	15	0	A_32_P153892	NM_031923	0,492407747	8,342195901	3,240606026	0,019217709	0,843980537	-4,211741888
148	73	0	A_24_P892137	THC2495542	-1,086900967	5,675543581	-3,240048485	0,019230655	0,843980537	-4,211807669
124	41	0	A_32_P96188	ENST00000590308	-0,560023079	5,651070567	-3,238851997	0,019258469	0,843980537	-4,211948891
255	14	0	A_24_P332218	NM_001038705	0,652075428	7,070103429	3,238083937	0,019276348	0,843980537	-4,212039587
152	63	0	A_24_P213724	ENST00000493170	-0,437802694	6,376012149	-3,237649488	0,019286469	0,843980537	-4,212090902
293	7	0	A_24_P830667	NM_000982	0,762662314	10,5707005	3,237620198	0,019287151	0,843980537	-4,212094362
95	79	0	A_24_P53778	NM_080878	-0,740022798	5,576786105	-3,237045989	0,019300537	0,843980537	-4,212162202
182	24	0	A_23_P407905	ENST00000480186	0,578871757	5,987467571	3,236805203	0,019306154	0,843980537	-4,212190655
224	25	0	A_24_P707156	NM_001184749	0,626400093	5,685832881	3,23667409	0,019309213	0,843980537	-4,21220615
88	81	0	A_24_P452326	A_24_P452326	-0,998412114	6,344645679	-3,236596322	0,019311027	0,843980537	-4,212215341
28	83	0	A_24_P8524	A_24_P8524	-0,745278102	5,963648938	-3,236577356	0,01931147	0,843980537	-4,212217582
87	67	0	A_24_P945165	ENST00000371316	-1,229126586	5,47932569	-3,236151506	0,01932141	0,843980537	-4,212267917
62	49	0	A_32_P137849	THC2772705	-0,473813261	7,152386801	-3,234748312	0,019354205	0,843980537	-4,212433841
194	24	0	A_23_P130677	NM_018687	0,484229258	6,492071757	3,234219732	0,019366574	0,843980537	-4,212496372
149	69	0	A_23_P138089	NM_000530	-1,020580286	6,740043681	-3,232055159	0,01941732	0,843980537	-4,212752598
234	20	0	A_24_P414376	NM_016531	0,590306199	8,901935651	3,231436022	0,019431862	0,843980537	-4,212825933
305	54	0	A_23_P79441	NM_017880	0,505975168	7,847532084	3,229858889	0,019468958	0,843980537	-4,213012836
252	74	0	A_23_P88776	NM_001080492	-0,667464827	6,598157252	-3,227660838	0,019520791	0,843980537	-4,213273547
153	28	0	A_32_P775679	U22172	0,513598185	5,319232272	3,226599043	0,019545884	0,843980537	-4,21339958
157	1	0	A_23_P100595	ENST00000584952	0,656314757	6,954586284	3,225499212	0,019571913	0,843980537	-4,213530193
310	35	0	A_23_P27040	NM_015544	-0,451501019	12,11758829	-3,225175408	0,019579584	0,843980537	-4,21356866
180	77	0	A_24_P340116	NM_021806	-1,157394634	6,921241801	-3,225079855	0,019581848	0,843980537	-4,213580012
156	80	0	A_24_P911663	NM_001080414	-0,590769549	6,546843623	-3,22496962	0,01958446	0,843980537	-4,21359311
252	46	0	A_32_P30991	THC2705074	0,485993488	6,566173084	3,224381516	0,019598405	0,843980537	-4,213662995
199	71	0	A_24_P308851	NM_032087	-0,572070711	7,121537637	-3,223982581	0,01960787	0,843980537	-4,213710412
526	11	0	A_24_P212715	A_24_P212715	-0,467347611	7,272114764	-3,223803043	0,019612131	0,843980537	-4,213731755
428	21	0	A_23_P86570	NM_004034	0,612905434	8,246230495	3,223173308	0,019627086	0,843980537	-4,213806628
79	65	0	A_23_P300847	ENST00000555882	-0,613475034	5,861728404	-3,219381754	0,019717394	0,843980537	-4,214257887
129	66	0	A_24_P509893	AK056358	-0,55003332	6,678046103	-3,218972232	0,019727175	0,843980537	-4,214306674
444	12	0	A_24_P220472	NM_181727	-0,472654159	5,433131715	-3,218936069	0,019728039	0,843980537	-4,214310983
93	74	0	A_23_P16754	NM_198582	-1,110846404	6,639881876	-3,218039407	0,019749476	0,843980537	-4,214417837
226	62	0	A_24_P74997	THC2786072	0,631441238	6,379225884	3,217259174	0,01976815	0,843980537	-4,214510852
313	11	0	A_23_P28898	NM_000933	0,55292113	8,688054275	3,217242513	0,019768549	0,843980537	-4,214512838
105	64	0	A_23_P119344	NM_003598	-0,589832005	7,801980365	-3,215547718	0,01980918	0,843980537	-4,214714999
31	52	0	A_32_P30330	AK130878	-0,709035407	6,636058028	-3,214645569	0,019830846	0,843980537	-4,214822674
144	76	0	A_24_P921485	A_24_P921485	-0,663262234	5,766465631	-3,213726233	0,019852952	0,843980537	-4,214932446
87	64	0	A_23_P205867	NM_014249	-0,563159254	6,750922188	-3,213678543	0,0198541	0,843980537	-4,214938141
127	72	0	A_32_P115475	NM_001284514	-0,852550865	6,372794941	-3,211775328	0,019899952	0,843980537	-4,215165543
436	51	0	A_24_P323148	NM_182573	0,870231346	6,219277161	3,211099899	0,019916253	0,843980537	-4,215246293
148	61	0	A_23_P252250	AK022183	-0,4541213	8,285053324	-3,21087342	0,019921722	0,843980537	-4,215273375
145	54	0	A_24_P486924	A_24_P486924	-0,449445231	5,597105636	-3,210018618	0,019942379	0,843980537	-4,215375616
240	22	0	A_23_P401547	NM_015480	0,664981091	8,350108233	3,209191415	0,019962392	0,843980537	-4,215474594
264	66	0	A_32_P130630	NM_004731	-0,502101494	6,544299601	-3,208646378	0,01997559	0,843980537	-4,21553983
168	9	0	A_32_P13081	A_32_P13081	0,549910298	6,8720921	3,206439606	0,020029126	0,843980537	-4,215804128
515	7	0	A_32_P92505	NM_182551	0,427003698	9,393853199	3,205945619	0,020041132	0,843980537	-4,215863328
486	10	0	A_24_P76319	A_24_P76319	0,646525411	6,187690113	3,205841582	0,020043661	0,843980537	-4,215875797
190	67	0	A_24_P915117	A_24_P915117	-0,652279281	6,151146581	-3,205670243	0,020047828	0,843980537	-4,215896335
444	47	0	A_32_P126698	NM_001284269	0,712514591	6,195808883	3,204773784	0,020069644	0,843980537	-4,216003814
205	36	0	A_23_P8408	NM_001099220	-0,495611194	6,920865359	-3,203221685	0,020107477	0,843980537	-4,216190005
95	66	0	A_24_P13649	NM_032714	-0,580331464	7,629483871	-3,203050985	0,020111643	0,843980537	-4,216210491
168	10	0	A_23_P500998	NM_152739	0,818643637	5,786241832	3,202114839	0,020134505	0,843980537	-4,216322864

505	75	0	A_23_P34510	NM_198040	-0,51730785	7,514591623	-3,2010153	0,020161394	0,843980537	-4,216454912
487	35	0	A_23_P134663	NM_024812	0,490259572	7,185697328	3,200967706	0,020162559	0,843980537	-4,216460629
114	15	0	A_24_P463658	THC2730580	0,588575473	6,132848724	3,200907584	0,020164031	0,843980537	-4,216467852
407	61	0	A_24_P497464	NR_103738	0,704281602	7,779024587	3,199025373	0,020210159	0,843980537	-4,216694059
7	42	0	A_23_P304311	BC000189	-0,516554229	5,543993925	-3,198546296	0,020221918	0,843980537	-4,216751667
85	74	0	A_24_P296792	NM_002698	-0,587269488	6,43440236	-3,197853388	0,02023894	0,843980537	-4,216835009
88	79	0	A_32_P93558	A_32_P93558	-0,633955339	5,521850887	-3,197051783	0,020258652	0,843980537	-4,216931458
131	82	0	A_23_P18292	NM_001034996	0,494928584	13,9198031	3,196973774	0,020260571	0,843980537	-4,216940845
326	2	0	A_24_P13831	NM_182854	0,830212795	6,18011549	3,19674608	0,020266174	0,843980537	-4,216968249
210	31	0	A_24_P274831	NM_153236	0,585391315	5,664917522	3,19536244	0,020300262	0,843980537	-4,217134835
140	13	0	A_23_P73097	NM_170587	0,633790685	7,30050054	3,195241317	0,020303249	0,843980537	-4,217149422
110	68	0	A_24_P315547	XM_005257848	-0,78377799	5,870774513	-3,194523884	0,020320952	0,843980537	-4,217235845
237	1	0	A_24_P478617	NR_037881	0,805103088	6,388114943	3,19419073	0,020329178	0,843980537	-4,217275987
183	1	0	A_24_P381604	NM_021999	0,66370087	6,869791651	3,192674116	0,020366674	0,843980537	-4,217458801
189	78	0	A_24_P170025	A_24_P170025	-0,595020229	6,670556262	-3,192056385	0,020381968	0,843980537	-4,217533298
265	73	0	A_23_P20532	ENST00000276974	0,560452035	7,171439054	3,191538548	0,020394798	0,843980537	-4,217595766
342	23	0	A_24_P696009	A_24_P696009	0,624677224	5,373290209	3,190433726	0,020422203	0,843980537	-4,21772909
240	25	0	A_23_P413585	NM_004474	0,817972958	5,654062132	3,190308726	0,020425306	0,843980537	-4,217744179
175	16	0	A_24_P501285	AL080072	0,567491585	5,78447708	3,190154507	0,020429135	0,843980537	-4,217762796
85	82	0	A_24_P881608	A_24_P881608	0,54032315	13,0772875	3,189539171	0,020444421	0,843980537	-4,21783709
78	83	0	A_23_P401524	BC029818	0,75556175	10,35683198	3,188477824	0,020470817	0,843980537	-4,217965284
265	82	0	A_32_P64668	TCONS_l2_00007928	-0,705275449	6,330526519	-3,187593428	0,020492841	0,843980537	-4,218072153
178	58	0	A_24_P911094	ENST00000376439	-0,434158268	6,497604663	-3,186798912	0,020512648	0,843980537	-4,218168197
90	2	0	A_24_P628797	A_24_P628797	0,586024315	6,598136216	3,185120862	0,020554553	0,843980537	-4,218371162
413	26	0	A_32_P452382	ENST00000435356	-0,451620082	5,831985188	-3,185046881	0,020556403	0,843980537	-4,218380113
180	19	0	A_24_P93341	ENST00000331659	0,913015093	5,821269151	3,184806583	0,020562412	0,843980537	-4,218409192
159	69	0	A_24_P698816	A_24_P698816	-0,859318295	5,63553274	-3,184301444	0,02057505	0,843980537	-4,218470328
98	78	0	A_32_P413094	NR_001536	-1,207371555	6,242966963	-3,182993384	0,020607816	0,843980537	-4,218628708
477	47	0	A_24_P360601	NM_017948	0,509196312	10,65332599	3,182430249	0,02062194	0,843980537	-4,218696922
163	25	0	A_24_P934729	AF264627	0,593838438	7,956174679	3,181973903	0,020633394	0,843980537	-4,218752212
80	85	0	A_23_P32199	NM_000755	-0,732745162	7,961530301	-3,18151704	0,020644867	0,843980537	-4,218807577
230	14	0	A_32_P195719	NR_033773	0,630698012	6,139433159	3,181066222	0,020656196	0,843980537	-4,218862221
189	20	0	A_24_P324838	AK128568	0,713526047	6,394027689	3,181002418	0,0206578	0,843980537	-4,218869956
254	22	0	A_24_P919640	U94903	0,802395494	6,445371925	3,179961358	0,020683989	0,843980537	-4,218996189
207	61	0	A_24_P174346	NM_001002258	-0,509305554	7,346483102	-3,178539003	0,020719829	0,843980537	-4,219168753
310	61	0	A_23_P384965	ENST00000498717	0,712297814	6,574499045	3,175394681	0,020799303	0,843980537	-4,219550629
203	81	0	A_23_P217399	NM_002641	-0,83781978	6,518520224	-3,170534998	0,020922795	0,843980537	-4,22014191
247	30	0	A_23_P217899	NM_030937	-0,458865505	10,29116742	-3,169902258	0,020938934	0,843980537	-4,220218992
280	41	0	A_24_P924671	A_24_P924671	1,212625973	6,53572657	3,169863158	0,020939931	0,843980537	-4,220223756
136	66	0	A_32_P91005	BM697215	-0,629744622	6,849277149	-3,169582188	0,020947102	0,843980537	-4,220257993
144	63	0	A_32_P140153	ENST00000412050	-0,499475946	5,976723657	-3,169303999	0,020954205	0,843980537	-4,220291894
125	70	0	A_23_P396818	NM_139285	-0,715873657	6,775513677	-3,168219346	0,020981924	0,843980537	-4,220424118
154	84	0	A_24_P692426	A_24_P692426	-0,4667213	6,149372959	-3,167389557	0,021003157	0,843980537	-4,220525316
340	78	0	A_32_P345444	ENST00000587624	-0,657314506	6,22530575	-3,167116008	0,021010162	0,843980537	-4,220558686
54	55	0	A_24_P12573	NM_006072	-0,426680038	5,333500553	-3,166486674	0,021026287	0,843980537	-4,220635473
335	11	0	A_23_P46426	NM_001554	0,570829562	8,264833503	3,165139592	0,021060849	0,843980537	-4,220799908
191	2	0	A_23_P4400	NM_033059	0,472702981	8,913374932	3,163330455	0,021107364	0,843980537	-4,221020903
14	68	0	A_23_P86470	NM_003956	-0,683150716	5,440137349	-3,163283231	0,02110858	0,843980537	-4,221026674
487	48	0	A_23_P325562	NM_006671	0,667033049	7,591983561	3,162379936	0,021131849	0,843980537	-4,221137087
112	76	0	A_24_P926693	AK001038	-0,631836184	6,031033296	-3,161190916	0,021162521	0,843980537	-4,221282494
123	56	0	A_24_P388502	NM_022485	-0,585256042	6,017565938	-3,160945507	0,021168858	0,843980537	-4,221312515
204	27	0	A_23_P203972	NM_007197	0,570714304	6,841261618	3,156565518	0,021282306	0,843980537	-4,22184889
447	38	0	A_24_P669088	ENST00000414565	0,69848606	5,394082449	3,156117724	0,021293942	0,843980537	-4,221903787
515	52	0	A_23_P370479	NM_015352	0,622537957	4,639685659	3,155811363	0,021301907	0,843980537	-4,221941351
201	20	0	A_24_P942132	NM_001079537	0,908753839	5,561846822	3,155555011	0,021308575	0,843980537	-4,221972788
512	10	0	A_23_P112452	NR_003191	-0,41637381	5,637990725	-3,154749406	0,021329542	0,843980537	-4,222071604
289	50	0	A_32_P885640	NR_038850	0,473343987	6,547840922	3,15417469	0,021344515	0,843980537	-4,222142122
182	69	0	A_24_P938036	A_24_P938036	-0,63887891	6,094959175	-3,152372366	0,021391543	0,843980537	-4,222363385
157	81	0	A_32_P76804	THC2787201	-0,502634702	5,657684691	-3,151234067	0,021421304	0,843980537	-4,222503223
361	16	0	A_23_P116578	NM_015885	0,46004974	7,99142845	3,149878388	0,021456807	0,843980537	-4,22266986
391	7	0	A_24_P920227	A_24_P920227	0,555230964	6,033228807	3,148284843	0,021498623	0,843980537	-4,222865865
328	2	0	A_24_P372959	NM_018318	0,578191749	8,120227643	3,147373281	0,021522584	0,843980537	-4,222978051
176	37	0	A_24_P493100	A_24_P493100	0,598201779	5,304170467	3,146198697	0,021553501	0,843980537	-4,223122676
171	83	0	A_23_P104438	NM_032578	-0,989486341	6,697442273	-3,145764565	0,021564941	0,843980537	-4,223176149
194	69	0	A_24_P929629	A_24_P929629	-0,700109707	6,133999066	-3,145017319	0,021584647	0,843980537	-4,223268214
151	74	0	A_24_P226278	NM_015288	-0,47171168	7,885885639	-3,143819221	0,021616284	0,843980537	-4,223415893
214	1	0	A_23_P161424	NM_032812	0,51312438	6,766159224	3,143454848	0,021625916	0,843980537	-4,223460821
136	83	0	A_24_P941625	NM_021916	-0,440786198	7,381459072	-3,142414957	0,02165343	0,843980537	-4,223589085
126	33	0	A_24_P931568	A_24_P931568	0,56164173	5,651632371	3,141467093	0,021678543	0,843980537	-4,223706051
199	81	0	A_23_P106405	NM_002487	-0,76303877	6,044420439	-3,141367834	0,021681175	0,843980537	-4,223718303
331	16	0	A_23_P118675	NM_002144	0,621952499	5,979812961	3,136837512	0,021801663	0,843980537	-4,224278062
191	85	0	A_23_P51745	NM_032414	-0,773446301	5,855548515	-3,136514928	0,02181027	0,843980537	-4,224317964
24	53	0	A_24_P84099	NM_001144872	-0,472618906	5,928134692	-3,135991445	0,021824246	0,843980537	-4,224382729
335	79	0	A_24_P118341	A_24_P118341	-0,445183378	10,67607876	-3,131788828	0,021936806	0,843980537	-4,224903227
338	81	0	A_24_P915926	ENST00000496396	-1,028419366	6,232886437	-3,129979557	0,021985461	0,843980537	-4,225127613
89	69	0	A_24_P103060	NM_139279	-0,5626301	7,431193942	-3,129635017	0,02199474	0,843980537	-4,225170364
152	75	0	A_23_P110175	NM_001334	-0,804433218	5,375626738	-3,126975862	0,022066499	0,843980537	-4,225500538
483	40	0	A_23_P407583	NM_006210	0,424410334	6,752751587	3,125422786	0,022108529	0,843980537	-4,22569356
65	76	0	A_23_P314145	NM_005715	-0,835513877	5,487547336	-3,123547637	0,022159393	0,843980537	-4,225926792
238	63	0	A_24_P765715	THC2535569	-0,428535072	6,612564564	-3,121212711	0,022222908	0,843980537	-4,226217487
377	22	0	A_32_P185943	A_32_P185943	0,763558659	5,71346207	3,120965487	0,022229644	0,843980537	-4,226248284
414	20	0	A_32_P40184	NM_021248	0,793142133	7,32660645	3,120842526	0,022232996	0,843980537	-4,226263603
320	15	0	A_32_P3290	NM_017645	0,467458063	10,49085688	3,120797938	0,022234211	0,843980537	-4,226269158
498	38	0	A_32_P168727	A_32_P168727	0,490258316	7,707097307	3,120482262	0,022242819	0,843980537	-4,226308491
402	40	0	A_23_P345068	ENST00000458085	0,637462496	5,845352101	3,119482854	0,022270093	0,843980537	-4,226433051
277	46	0	A_32_P48397	NM_012401	-0,474447144	11,01652426	-3,118639262	0,022293143	0,843980537	-4,226538235
506	38	0	A_32_P105228	ENST00000590149	0,576833203	6,027190905	3,117830494	0,022315267	0,843980537	-4,226639115
108	48	0	A_23_P374281	NM_152623	-0,569968358	5,366035616	-3,117162176	0,022333567	0,843980537	-4,226722504
202	4	0	A_23_P218369	NM_032963	0,562909509	6,63589268	3,117024819	0,02233733	0,843980537	-4,226739646
471	83	0	A_32_P75068	THC2688348	-0,822522589	6,180639067	-3,115560535	0,022377491	0,843980537	-4,226922451
109	64	0	A_23_P84063	NM_016522	-0,489879174	5,659178858	-3,115171244	0,022388181	0,843980537	-4,226971072
199	43	0	A_24_P933706	XR_108763	-0,485654972	6,679492515	-3,114512265	0,02240629	0,843980537	-4,227053395
186	83	0	A_24_P15563	ENST00000554125	-0,52604935	7,075845933	-3,113971816	0,022421154	0,843980537	-4,227120928
192	73	0	A_23_P132308	NM_032561	-0,738523937	7,065105991	-3,113828868	0,022425087	0,843980537	-4,227138794

510	41	0	A_24_P337012	NM_138402	0,755829951	6,268988102	3,113552082	0,022432705	0,843980537	-4,227173389
376	82	0	A_23_P325040	NM_003276	-0,526892812	7,847439497	-3,113305422	0,022439496	0,843980537	-4,227204223
51	73	0	A_24_P397489	NM_001491	-1,107086949	5,548060004	-3,112018343	0,02247497	0,843980537	-4,227365169
224	75	0	A_24_P781757	NR_015434	-0,579795027	8,032503119	-3,11111491	0,022499906	0,843980537	-4,227478198
374	27	0	A_32_P160115	ENST00000561973	-0,448143449	5,111487909	-3,110536801	0,022515879	0,843980537	-4,227550549
480	41	0	A_24_P484965	ENST00000423031	0,56488626	5,398352167	3,110008654	0,022530483	0,843980537	-4,227616664
514	74	0	A_24_P16378	NM_152419	-0,847881737	5,954080652	-3,109841783	0,022535099	0,843980537	-4,227637557
127	83	0	A_32_P35047	NR_033878	-1,146182848	6,268243664	-3,109547339	0,022543247	0,843980537	-4,227674426
140	4	0	A_24_P347319	NM_139136	0,654131216	5,863830112	3,109503199	0,022544469	0,843980537	-4,227679954
508	48	0	A_24_P323131	NR_001538	0,574601083	6,386876085	3,107957382	0,0225873	0,843980537	-4,227873599
355	19	0	A_23_P122127	NM_001465	0,850976959	5,972865744	3,107358203	0,022603927	0,843980537	-4,227948695
169	17	0	A_24_P58597	A_24_P58597	0,409820841	7,633438986	3,107166476	0,022609249	0,843980537	-4,227972729
164	67	0	A_23_P45536	NM_005369	-0,55505825	6,23524506	-3,106803746	0,022619324	0,843980537	-4,228018204
193	81	0	A_24_P764690	ENST00000544044	-0,685868408	6,209614171	-3,106043436	0,022640456	0,843980537	-4,228113548
475	15	0	A_24_P778836	NM_015659	0,42278275	9,790733407	3,105743296	0,022648804	0,843980537	-4,228151195
500	35	0	A_32_P68381	ENST00000515306	0,649343683	5,861289147	3,105512195	0,022655235	0,843980537	-4,228180186
124	75	0	A_23_P8142	NM_001832	-0,959363825	6,577175227	-3,104686385	0,022678229	0,843980537	-4,228283806
93	52	0	A_24_P758323	ENST00000437348	-0,581395845	5,231618423	-3,104671613	0,02267864	0,843980537	-4,22828566
181	74	0	A_23_P56604	NM_003854	-0,591672933	6,108162186	-3,10192388	0,022755336	0,843980537	-4,228630716
301	13	0	A_32_P198412	ENST00000602412	0,636957171	8,991613194	3,101468553	0,022768072	0,843980537	-4,228687937
8	57	0	A_24_P24645	A_24_P24645	-0,699707818	6,197016275	-3,101151825	0,022776937	0,843980537	-4,228727747
252	17	0	A_32_P39210	A_32_P39210	0,444926136	6,460610124	3,10104294	0,022779985	0,843980537	-4,228741434
132	83	0	A_23_P323761	NM_025228	-0,500001989	6,200368988	-3,095433067	0,022937644	0,843980537	-4,229447528
448	2	0	A_24_P6764	NM_182626	-0,566422991	6,185908616	-3,095287789	0,022941742	0,843980537	-4,229465837
159	75	0	A_32_P83556	ENST00000435434	-0,426746227	6,34334488	-3,095029798	0,022949023	0,843980537	-4,229498355
206	69	0	A_32_P22139	NM_020786	-0,467490415	7,261948737	-3,09477567	0,022956197	0,843980537	-4,229530389
147	85	0	A_24_P161914	A_24_P161914	-0,772669923	6,924648873	-3,09417942	0,02297304	0,843980537	-4,229605564
368	25	0	A_23_P86461	ENST00000372263	-0,615496474	6,183118868	-3,094100446	0,022975271	0,843980537	-4,229615523
82	66	0	A_32_P394951	NM_145807	-0,735643627	7,188060589	-3,093979636	0,022978686	0,843980537	-4,229630758
127	75	0	A_24_P929106	ENST00000484265	-0,952849442	6,20912076	-3,093031113	0,023005514	0,843980537	-4,2297504
161	62	0	A_24_P935893	NM_017709	-0,604700688	5,413501262	-3,092705665	0,023014727	0,843980537	-4,229791462
367	15	0	A_23_P166219	NM_002040	0,705836431	8,077315974	3,091142496	0,023059035	0,843980537	-4,229988774
315	32	0	A_23_P127684	NM_003697	0,597553581	5,810245676	3,090560495	0,023075555	0,843980537	-4,230062273
464	74	0	A_32_P159023	A_32_P159023	0,471549159	7,358346651	3,090304015	0,02308284	0,843980537	-4,23009467
114	85	0	A_32_P133821	A_32_P133821	-0,861214448	7,083141815	-3,089944209	0,023093063	0,843980537	-4,230140123
390	54	0	A_23_P300600	NM_021076	0,444217269	11,3091107	3,089573694	0,023103596	0,843980537	-4,230186937
99	76	0	A_24_P280333	NM_182497	-0,897338788	6,053075487	-3,089257938	0,023112577	0,843980537	-4,230226839
406	75	0	A_24_P34168	NM_005964	0,513846474	8,449789279	3,089143741	0,023115825	0,843980537	-4,230241271
413	52	0	A_24_P384029	A_24_P384029	0,702088495	5,926152054	3,088407538	0,023136782	0,843980537	-4,230334331
22	39	0	A_23_P94689	NM_030914	-0,430967208	7,626728618	-3,086666924	0,023186413	0,843980537	-4,230554477
513	61	0	A_23_P107933	NM_016440	-0,547384168	7,249823152	-3,086627889	0,023187527	0,843980537	-4,230559416
78	65	0	A_23_P300136	AK074445	-0,663649924	5,910784063	-3,084169902	0,023257816	0,843980537	-4,230870591
200	67	0	A_24_P16913	NM_005845	-0,48041085	6,850028326	-3,084016484	0,023262211	0,843980537	-4,230890025
29	4	0	A_24_P742352	NR_026852	0,898067973	6,57092469	3,083905162	0,0232654	0,843980537	-4,230904128
119	85	0	A_24_P144963	AK056727	-1,073370038	6,397991794	-3,082626338	0,023302075	0,843980537	-4,23106618
214	6	0	A_23_P133854	NM_001007531	1,03234368	6,172184844	3,081567829	0,02333248	0,843980537	-4,231200384
208	19	0	A_23_P120594	NM_032501	0,460117119	7,067604314	3,080298628	0,023368994	0,843980537	-4,231361386
285	12	0	A_24_P678034	A_24_P678034	0,695111261	6,365353932	3,079410798	0,023394574	0,843980537	-4,231474065
151	76	0	A_23_P157766	NM_001039395	-1,063841049	6,213011903	-3,079306042	0,023397594	0,843980537	-4,231487363
226	37	0	A_24_P936298	A_24_P936298	0,542602549	5,139480253	3,078995088	0,023406562	0,843980537	-4,23152684
134	76	0	A_24_P910667	AF220235	-0,541302195	6,394437679	-3,078816019	0,023411728	0,843980537	-4,231549576
173	77	0	A_23_P53390	NM_002837	-0,789156473	6,615445572	-3,077618298	0,023446312	0,843980537	-4,231701697
76	85	0	A_24_P932810	A_24_P932810	-1,477587551	6,937715444	-3,077497582	0,023449801	0,843980537	-4,231717034
185	84	0	A_23_P112646	NM_020469	-0,473063346	5,841760256	-3,077390704	0,02345289	0,843980537	-4,231730613
192	14	0	A_23_P117363	NM_001756	0,457948362	7,68064615	3,074194441	0,023545487	0,843980537	-4,232137009
113	1	0	A_24_P929003	ENST00000573377	0,52148587	5,9472573	3,071832545	0,02361417	0,843980537	-4,232437694
23	81	0	A_23_P157495	NM_005605	0,543451794	8,086933157	3,071134723	0,023634505	0,843980537	-4,232526592
95	77	0	A_23_P371682	NM_005708	-0,518229766	7,27349123	-3,070804072	0,023644147	0,843980537	-4,232568725
158	77	0	A_24_P854440	ENST00000519996	-0,809973228	6,132096411	-3,070022211	0,023666963	0,843980537	-4,232668378
389	64	0	A_32_P11733	THC2743491	1,087987564	6,211560035	3,068065398	0,023724174	0,843980537	-4,232917938
244	3	0	A_23_P70677	NM_001135575	0,445884557	7,15458123	3,068003803	0,023725977	0,843980537	-4,232925797
478	39	0	A_32_P196047	NM_181787	0,472719352	9,313589442	3,067132018	0,023751516	0,843980537	-4,233037054
214	10	0	A_24_P457345	THC2692336	0,464137201	5,288369099	3,066644238	0,023765818	0,843980537	-4,233099323
123	66	0	A_24_P753476	NR_002942	-0,758826889	6,484034212	-3,065185672	0,023808643	0,843980537	-4,233285602
272	46	0	A_24_P456367	AK021614	-0,535344739	6,392125831	-3,064238647	0,023836494	0,843980537	-4,233406616
173	21	0	A_23_P403443	NM_173593	0,430753481	5,819500091	3,06368983	0,02385265	0,843980537	-4,233476769
276	83	0	A_24_P40358	THC2540041	-0,682054656	7,125934262	-3,061861503	0,02390656	0,843980537	-4,233710602
218	4	0	A_23_P29953	NM_172175	0,449644124	9,227289068	3,061041198	0,023930791	0,843980537	-4,233815577
363	17	0	A_23_P431638	ENST00000396825	0,678799345	8,714732959	3,059271804	0,02398314	0,843980537	-4,23404214
176	68	0	A_32_P99549	BC045573	-0,624267736	6,567486409	-3,058377477	0,02400966	0,843980537	-4,234156722
285	19	0	A_24_P413286	A_24_P413286	0,793488924	6,693206242	3,058087628	0,024018259	0,843980537	-4,234193868
115	71	0	A_24_P206603	AK001176	-0,503951713	6,203769088	-3,057597167	0,024032818	0,843980537	-4,234256735
318	13	0	A_24_P41309	BC045662	0,917821963	6,492621452	3,056509777	0,024065131	0,843980537	-4,234396164
505	43	0	A_23_P112666	NM_012186	0,612036372	6,49955358	3,056026368	0,024079511	0,843980537	-4,234458171
527	42	0	A_24_P463929	NM_001195597	0,674781638	5,850955463	3,053803791	0,024145748	0,843980537	-4,234743433
149	3	0	A_23_P33664	NM_022142	0,471960241	6,377517026	3,05355847	0,024153071	0,843980537	-4,234774936
327	60	0	A_23_P358995	NM_015525	0,419835888	10,35606286	3,051648916	0,024210158	0,843980537	-4,235020277
355	45	0	A_32_P147001	THC2655314	0,618723987	4,920597657	3,050920449	0,024231975	0,843980537	-4,235113926
438	18	0	A_24_P306644	A_24_P306644	-0,616456762	6,568161065	-3,050633699	0,024240569	0,843980537	-4,235150798
256	23	0	A_23_P30687	NM_004155	0,485442622	5,903480038	3,047782819	0,024326191	0,843980537	-4,23551764
372	66	0	A_32_P143850	NR_026540	0,548863256	7,021241873	3,047140559	0,024345526	0,843980537	-4,235600349
293	11	0	A_24_P876734	NR_038260	0,685957607	7,072338158	3,047083857	0,024347234	0,843980537	-4,235607652
76	83	0	A_24_P381975	NM_001077416	-0,964843992	5,880457406	-3,046618566	0,024361253	0,843980537	-4,235667588
130	6	0	A_23_P3221	NM_021199	0,543083682	7,932445492	3,045490646	0,024395274	0,843980537	-4,23581293
159	63	0	A_23_P165796	NM_000523	-0,662556791	6,701071857	-3,045455646	0,024396331	0,843980537	-4,235817441
147	84	0	A_23_P70634	NM_002511	-0,702443024	6,12590612	-3,044461307	0,024426367	0,843980537	-4,235945634
185	20	0	A_32_P111678	NR_026731	0,545127718	6,646169766	3,044107974	0,02443705	0,843980537	-4,2359912
241	1	0	A_23_P23894	NM_015375	0,483861167	9,029361008	3,043844932	0,024445007	0,843980537	-4,236025127
442	70	0	A_23_P434944	NM_004516	-0,434077272	10,08782432	-3,040454211	0,024547823	0,843980537	-4,236462818
113	23	0	A_23_P373017	D00044	0,59643829	6,379267485	3,038334277	0,024612346	0,843980537	-4,236736808
382	46	0	A_24_P237511	NM_004681	1,112521603	6,423390796	3,03795329	0,024623962	0,843980537	-4,236786076
95	61	0	A_24_P921727	A_24_P921727	-0,483338533	5,813365142	-3,036650398	0,02466373	0,843980537	-4,236954626
169	78	0	A_32_P179572	AJ001827	-0,453685713	7,162350823	-3,036442296	0,024670089	0,843980537	-4,236981557
119	71	0	A_23_P85453	NM_016382	-0,642418112	5,722493409	-3,036394038	0,024671564	0,843980537	-4,236987802

491	33	0	A_23_P203540	NM_012153	0,778029524	5,249887993	3,036356213	0,02467272	0,843980537	-4,236992697
364	24	0	A_24_P813667	AF116659	-0,438290384	5,881918714	-3,036258465	0,024675707	0,843980537	-4,237005348
31	67	0	A_24_P934355	NM_000620	-0,619700148	6,377141034	-3,034345395	0,024734259	0,843980537	-4,237253056
429	85	0	A_23_P213754	NM_001033112	0,439453348	11,65128016	3,033734717	0,024752982	0,843980537	-4,237332173
167	73	0	A_24_P918527	ENST00000562680	-0,47111867	7,941336078	-3,033622964	0,024756409	0,843980537	-4,237346653
138	23	0	A_23_P86064	NM_024746	0,618566045	5,918806258	3,032644646	0,02478644	0,843980537	-4,237473451
167	83	0	A_24_P76694	A_24_P76694	-0,617184536	5,531460394	-3,032345878	0,024795619	0,843980537	-4,237512184
264	35	0	A_23_P201901	BX537432	-0,530334415	6,357709449	-3,032305222	0,024796869	0,843980537	-4,237517456
327	37	0	A_23_P339687	NM_138330	0,548624545	8,031422965	3,032244277	0,024798742	0,843980537	-4,237525358
425	71	0	A_24_P932594	NM_014683	-0,50156473	6,371679998	-3,030660674	0,024847464	0,843980537	-4,23773076
156	79	0	A_23_P86489	X90828	-0,905897638	5,938997955	-3,029660671	0,024878285	0,843980537	-4,23786054
48	5	0	A_23_P146765	NM_007218	0,504345013	9,598090273	3,028946628	0,024900318	0,843980537	-4,237953245
166	23	0	A_24_P333525	NM_014857	0,607657018	7,355424494	3,02744146	0,024946833	0,843980537	-4,238148758
69	84	0	A_24_P350126	A_24_P350126	0,544178962	14,04861054	3,025757275	0,024998993	0,843980537	-4,238367681
288	24	0	A_23_P148829	NM_080431	0,684382734	6,246192018	3,025564837	0,02500496	0,843980537	-4,238392707
102	19	0	A_32_P49832	NM_001042475	0,71886468	6,276904588	3,025244042	0,025014911	0,843980537	-4,238434428
438	11	0	A_24_P116909	NM_006785	-0,448395235	6,344040855	-3,023657805	0,025064181	0,843980537	-4,238640818
531	70	0	A_24_P291074	A_24_P291074	-0,430351325	5,612546769	-3,022182016	0,025110116	0,843980537	-4,238832968
37	77	0	A_23_P147121	NM_003437	-0,717089283	7,397856694	-3,020043016	0,025176858	0,843980537	-4,239111695
191	6	0	A_23_P150189	NM_005590	0,395800293	8,374577615	3,018990664	0,025209765	0,843980537	-4,239248922
224	82	0	A_23_P45811	NM_000792	-0,694013588	6,283001367	-3,018178052	0,025235208	0,843980537	-4,23935493
481	82	0	A_24_P271014	NM_001042388	-0,896427349	6,630721337	-3,017564479	0,025254438	0,843980537	-4,239434999
26	66	0	A_24_P24444	NM_005107	-0,48334475	5,728960982	-3,016358853	0,025292269	0,843980537	-4,239592393
48	46	0	A_23_P385766	NM_145865	-0,427442588	4,650815327	-3,01621092	0,025296915	0,843980537	-4,239611711
45	28	0	A_23_P27894	NM_014649	-0,398612133	8,666092634	-3,016204364	0,025297121	0,843980537	-4,239612567
92	56	0	A_23_P352648	ENST00000580125	-0,393594626	6,18867015	-3,015633566	0,025315058	0,843980537	-4,239687119
400	16	0	A_24_P932385	A_24_P932385	0,589978906	5,804022581	3,015209818	0,025328383	0,843980537	-4,239742477
249	32	0	A_23_P107454	NM_031958	0,547538274	5,680109682	3,014146661	0,025361847	0,843980537	-4,239881413
27	44	0	A_23_P398530	NM_133334	-0,496859509	6,72595521	-3,013563218	0,025380233	0,843980537	-4,239957687
482	20	0	A_32_P234414	THC2621536	0,606188846	5,419452238	3,012809878	0,025403994	0,843980537	-4,240056201
330	17	0	A_23_P430411	XM_005261115	0,796879836	6,005084998	3,011622473	0,025441495	0,843980537	-4,240211545
146	57	0	A_23_P202388	NM_001034954	-0,617047613	6,247146947	-3,011143085	0,025456652	0,843980537	-4,240274285
512	36	0	A_32_P61998	AW874698	0,495789586	6,10155151	3,010451852	0,025478525	0,843980537	-4,240364774
344	2	0	A_23_P143034	AK001904	0,762145002	5,686953229	3,009787871	0,025499555	0,843980537	-4,240451721
122	84	0	A_24_P350941	AF116632	-1,203394711	5,376798878	-3,008771487	0,025531783	0,843980537	-4,240584865
248	50	0	A_24_P322771	NM_003225	0,732492231	7,028763109	3,008185116	0,02555397	0,843980537	-4,240661706
385	20	0	A_24_P75220	NM_004742	-0,428006898	6,444397046	-3,008004145	0,025556145	0,843980537	-4,240685426
176	72	0	A_23_P8328	NM_001526	-0,593135939	5,363999398	-3,007710278	0,025565481	0,843980537	-4,240723946
177	85	0	A_23_P155931	NM_020203	-0,544876214	6,65586148	-3,006507786	0,025603723	0,843980537	-4,240881623
155	85	0	A_24_P932066	NM_018126	-0,668235567	5,856868001	-3,006436075	0,025606006	0,843980537	-4,240891028
161	68	0	A_24_P416543	NM_016626	-0,4616762	7,309048915	-3,005282659	0,02564275	0,843980537	-4,241042355
136	61	0	A_23_P117679	NM_013309	-0,467801122	6,706334589	-3,00486035	0,025656218	0,843980537	-4,24109778
52	17	0	A_23_P141055	NM_001042454	-0,419408985	8,026886742	-3,004399862	0,025670912	0,843980537	-4,241158228
30	62	0	A_23_P125435	NM_000812	-0,580436698	6,065006991	-3,004037786	0,025682473	0,843980537	-4,241205767
93	83	0	A_24_P886960	ENST00000442130	-0,716447484	5,908702817	-3,003878043	0,025687575	0,843980537	-4,241226743
424	80	0	A_32_P75122	NM_152274	0,43443151	9,66719035	3,001838787	0,025752805	0,843980537	-4,241494648
190	74	0	A_24_P927507	BC041563	-0,471701118	6,646476179	-3,001171133	0,025774201	0,843980537	-4,241582413
337	2	0	A_24_P914859	A_24_P914859	0,438712728	6,156902963	2,999808774	0,025817921	0,843980537	-4,241761581
140	35	0	A_24_P913156	ENST00000513302	0,589824536	6,265778703	2,999376665	0,025831804	0,843980537	-4,241818431
527	10	0	A_24_P169258	NM_005993	-0,407866471	7,863805939	-2,998805683	0,025850162	0,843980537	-4,241893569
122	76	0	A_23_P44857	ENST00000390455	-0,597691851	6,24539633	-2,998290379	0,025866743	0,843980537	-4,241961397
31	72	0	A_32_P213706	ENST00000568063	-0,857199378	5,054263314	-2,997485546	0,025892662	0,843980537	-4,242067366
260	83	0	A_24_P926734	A_24_P926734	-0,480565277	6,315839049	-2,996531752	0,025923416	0,843980537	-4,242192997
99	80	0	A_24_P557355	AF070564	-1,148897476	6,123625953	-2,995847449	0,025945505	0,843980537	-4,242283164
306	77	0	A_24_P131236	NM_014849	-0,44352243	6,723920767	-2,995839523	0,025945761	0,843980537	-4,242284209
112	21	0	A_23_P128744	NM_000710	0,528089829	7,936903153	2,995351178	0,025961537	0,843980537	-4,242348572
195	68	0	A_23_P45180	NM_002099	-0,885461764	5,738124423	-2,994899832	0,025976127	0,843980537	-4,242408072
371	17	0	A_23_P84018	NM_000943	0,671911975	8,982444713	2,994242117	0,025997405	0,843980537	-4,242494799
143	77	0	A_23_P217507	NM_004729	-0,494924091	7,163702761	-2,994169992	0,025999739	0,843980537	-4,242504311
447	12	0	A_24_P940188	NM_014870	-0,481136869	5,611894639	-2,993785663	0,026012182	0,843980537	-4,242555002
361	6	0	A_32_P426044	NM_203408	0,899864394	5,896754592	2,993522704	0,0260207	0,843980537	-4,24258969
474	38	0	A_23_P119835	NM_021209	1,019013821	5,218894322	2,992792663	0,026044363	0,843980537	-4,242686014
117	83	0	A_23_P142310	NM_017572	0,421008854	13,18346038	2,99259146	0,026050889	0,843980537	-4,242712567
150	32	0	A_23_P53057	NM_013250	0,422464223	5,534832874	2,991385628	0,026090036	0,843980537	-4,242871751
381	82	0	A_32_P216030	ENST00000532562	-0,91806269	6,130242434	-2,98896194	0,026168915	0,843980537	-4,243191965
336	5	0	A_24_P735072	NM_001284514	0,583104018	5,430954651	2,98860221	0,026180645	0,843980537	-4,243239522
24	77	0	A_32_P737256	NR_027267	-0,472341872	5,830185476	-2,987677171	0,026210834	0,843980537	-4,243361848
240	28	0	A_32_P508722	TCONS_00029313	0,729302354	5,687001734	2,98707251	0,026230588	0,843980537	-4,243441834
458	81	0	A_32_P65954	A_32_P65954	-0,759940402	6,440274609	-2,986565849	0,026247153	0,843980537	-4,243508874
495	77	0	A_24_P221335	A_24_P221335	-0,456643651	6,715979777	-2,986448956	0,026250976	0,843980537	-4,243524342
179	78	0	A_23_P216935	NR_026677	-0,553970228	6,320884692	-2,985963497	0,026266861	0,843980537	-4,243588594
235	59	0	A_32_P197156	NR_102696	-0,548633988	5,386445178	-2,984886206	0,02630215	0,843980537	-4,243731225
131	14	0	A_24_P599340	NM_015550	0,687699292	5,817452321	2,98469628	0,026308376	0,843980537	-4,243756379
30	80	0	A_23_P166584	NM_130830	-0,553248506	6,236566169	-2,984307408	0,026321131	0,843980537	-4,243807886
504	49	0	A_24_P825874	NM_001277406	0,41965282	6,826409379	2,983846405	0,026336259	0,843980537	-4,243868958
115	75	0	A_32_P138833	NM_001145678	-0,676703632	6,093932455	-2,983588011	0,026344743	0,843980537	-4,243903195
45	59	0	A_23_P43504	NM_001606	-0,525667006	7,838346092	-2,98340674	0,026350697	0,843980537	-4,243927216
41	71	0	A_24_P333216	XR_112023	-0,993359757	5,303059531	-2,982938636	0,026366077	0,843980537	-4,243989254
318	12	0	A_32_P2883	XR_158838	0,444145221	8,420424384	2,98031889	0,026452337	0,843980537	-4,24433669
177	81	0	A_23_P200361	NM_001286	-0,455312602	6,752685429	-2,980280493	0,026453604	0,843980537	-4,244341785
317	85	0	A_23_P159012	NM_023039	0,465623294	10,25892998	2,980146307	0,02645803	0,843980537	-4,244359592
26	10	0	A_23_P342484	NM_000567	0,641264663	5,790705489	2,979996774	0,026462964	0,843980537	-4,244379437
296	56	0	A_24_P164998	NM_001085474	-0,702116675	5,896845251	-2,978743697	0,026504352	0,843980537	-4,24454579
132	70	0	A_32_P231641	THC2671299	-0,676087873	6,313455897	-2,977958293	0,026530328	0,843980537	-4,244650104
177	58	0	A_24_P350683	ENST00000374086	-0,5670378	6,622692754	-2,97702596	0,026561201	0,843980537	-4,244773979
246	15	0	A_23_P343303	NM_004188	0,760331558	6,305693729	2,976749301	0,026570369	0,843980537	-4,244810747
172	85	0	A_32_P214107	THC2671876	-0,518190203	5,320961146	-2,976666779	0,026573105	0,843980537	-4,244821716
103	82	0	A_23_P406508	NM_005020	-0,754651596	5,224734226	-2,976249031	0,026586957	0,843980537	-4,244877246
141	70	0	A_23_P214444	NM_000544	-0,73414396	5,705023734	-2,975933967	0,02659741	0,843980537	-4,244919133
442	29	0	A_23_P154420	NM_021193	0,73477298	6,072487334	2,97565304	0,026606734	0,843980537	-4,244956487
109	13	0	A_24_P101101	A_24_P101101	0,428706632	9,723571744	2,975651015	0,026606801	0,843980537	-4,244956756
163	82	0	A_23_P140967	NM_000243	-0,532340384	6,571582423	-2,975385386	0,026615621	0,843980537	-4,24499208
167	79	0	A_23_P104282	NM_018121	-0,706005683	7,30961041	-2,974952989	0,026629984	0,843980537	-4,24504959
188	34	0	A_23_P156025	NM_033267	0,533094674	4,675166512	2,974758783	0,026636438	0,843980537	-4,245075424

398	35	0	A_24_P213503	NM_006504	0,53840113	7,115162944	2,973581593	0,026675596	0,843980537	-4,245232063
72	69	0	A_24_P267814	CU676521	-1,014933512	5,691762095	-2,973209455	0,026687987	0,843980537	-4,245281598
199	73	0	A_24_P466231	THC2515749	-0,632811853	6,494335669	-2,973181716	0,026688911	0,843980537	-4,245285291
163	66	0	A_24_P762409	AU145411	-0,64431244	5,696490401	-2,973130553	0,026690615	0,843980537	-4,245292102
60	74	0	A_24_P282031	NM_015914	-0,876045736	6,003069973	-2,972684853	0,026705467	0,843980537	-4,245351441
454	42	0	A_32_P19466	A_32_P19466	0,482842462	7,335355741	2,972622618	0,026707541	0,843980537	-4,245359727
266	63	0	A_24_P105391	NM_001017922	-0,44979993	6,10631229	-2,97180686	0,026734749	0,843980537	-4,245468368
532	58	0	A_23_P397285	NM_017527	-0,673747007	6,43983058	-2,970307706	0,026784828	0,843980537	-4,245668125
160	33	0	A_23_P148473	NM_000206	0,61362312	5,009205826	2,969783571	0,026802361	0,843980537	-4,245737996
424	78	0	A_23_P54720	NM_001008393	-0,678240556	6,593213612	-2,968072042	0,026859702	0,843980537	-4,245966266
404	5	0	A_24_P357406	NM_001006121	0,552418724	5,758329492	2,967936164	0,02686426	0,843980537	-4,245984396
484	61	0	A_23_P39375	NM_018316	-0,442618565	7,586969625	-2,967888269	0,026865867	0,843980537	-4,245990787
392	76	0	A_23_P3368	NM_002569	-0,501005643	5,762320797	-2,967314769	0,026885115	0,843980537	-4,246067321
318	38	0	A_23_P148047	NM_000958	0,60973805	6,231771178	2,96681648	0,026901852	0,843980537	-4,246133834
499	5	0	A_24_P194707	NM_182493	0,453866507	5,509082391	2,966475917	0,026913297	0,843980537	-4,246179301
152	78	0	A_24_P248079	BC031319	-0,800131394	6,209206624	-2,966449503	0,026914185	0,843980537	-4,246182828
319	56	0	A_23_P7957	NM_018960	-0,489725385	6,711143315	-2,966431622	0,026914786	0,843980537	-4,246185215
35	84	0	A_24_P902764	A_24_P902764	-0,920414605	6,380651014	-2,965278994	0,026953564	0,843980537	-4,246339154
126	80	0	A_23_P213045	NM_016269	-1,127890217	6,794942778	-2,965104149	0,026959452	0,843980537	-4,246362512
409	71	0	A_24_P33575	XR_254201	-0,730242723	6,770687932	-2,964897254	0,026966421	0,843980537	-4,246390154
154	64	0	A_23_P433218	NR_002185	-0,417313473	6,184593395	-2,963196503	0,027023782	0,843980537	-4,246617479
447	22	0	A_23_P329798	NM_005454	0,565592112	5,777772669	2,962939481	0,027032462	0,843980537	-4,246651848
405	57	0	A_24_P16833	NM_001008781	0,497661171	6,186346075	2,962869045	0,027034841	0,843980537	-4,246661267
99	75	0	A_32_P102745	A_32_P102745	-0,551706124	6,092071541	-2,962672817	0,027041471	0,843980537	-4,24668751
408	49	0	A_23_P380724	NM_021826	0,424127678	11,33698089	2,961555089	0,027079268	0,843980537	-4,246837035
296	58	0	A_32_P234926	NM_001014977	0,783489551	6,41410768	2,960786124	0,027105304	0,843980537	-4,246939947
163	83	0	A_23_P111260	NM_002526	-1,197091607	6,312944674	-2,96012802	0,027127609	0,843980537	-4,24702805
338	63	0	A_23_P167941	NM_018965	0,924026036	6,208097215	2,959035441	0,027164684	0,843980537	-4,247174375
178	50	0	A_23_P22672	NM_001257231	0,4853036	10,24290607	2,958917852	0,027168677	0,843980537	-4,247190128
428	12	0	A_23_P94840	NM_130897	0,615446244	5,911498653	2,958824909	0,027171834	0,843980537	-4,247202579
465	42	0	A_23_P7727	NM_001884	0,51184867	5,613351481	2,958739581	0,027174733	0,843980537	-4,247214011
203	10	0	A_24_P272917	NM_001242326	0,69071675	6,131523541	2,957346643	0,027222099	0,843980537	-4,247400689
499	83	0	A_32_P46571	NM_017821	-0,739845428	5,967345923	-2,957181708	0,027227713	0,843980537	-4,247422801
266	5	0	A_24_P7181	A_24_P7181	0,5104961	11,0946134	2,955811683	0,027274399	0,843980537	-4,247606534
39	10	0	A_32_P209094	NM_018291	0,409400504	8,403886335	2,955703116	0,027278102	0,843980537	-4,247621099
317	82	0	A_32_P228804	NM_001278458	-0,421734791	6,306488168	-2,955662284	0,027279495	0,843980537	-4,247626577
80	72	0	A_23_P150343	NM_003063	-1,200694222	5,523582351	-2,955318981	0,02729121	0,843980537	-4,247672637
395	60	0	A_23_P13822	NM_018423	0,998859625	5,968911749	2,954299955	0,027326015	0,843980537	-4,2478094
7	12	0	A_23_P109774	NM_014415	0,541454774	8,659044964	2,953553465	0,027351542	0,843980537	-4,247909625
287	80	0	A_23_P87346	NM_000519	-0,93083141	6,725477424	-2,951796266	0,027411735	0,843980537	-4,248145681
157	9	0	A_23_P431912	NM_152577	0,616110939	6,149648227	2,951721077	0,027414313	0,843980537	-4,248155786
151	24	0	A_23_P42530	NM_015548	0,533915184	8,046973136	2,951122041	0,027434869	0,843980537	-4,248236303
100	81	0	A_23_P109864	NM_004122	-0,636412605	6,375907953	-2,950987353	0,027439492	0,843980537	-4,24825441
145	66	0	A_23_P20139	NM_016943	-0,920513192	5,692203623	-2,950843323	0,027444438	0,843980537	-4,248273773
253	75	0	A_23_P23630	NM_030649	-0,424442105	8,370872527	-2,949197132	0,027501033	0,843980537	-4,248495176
330	42	0	A_24_P353905	NM_032348	-0,412707918	7,978426269	-2,948495606	0,02752519	0,843980537	-4,248589577
196	8	0	A_24_P732565	THC2650367	0,626692285	6,644023393	2,948230196	0,027534336	0,843980537	-4,248625299
508	55	0	A_24_P8474	NM_002851	0,773228895	5,519366963	2,948215495	0,027534842	0,843980537	-4,248627278
504	34	0	A_23_P5685	NM_006593	0,656712396	5,138321638	2,947846514	0,027547562	0,843980537	-4,248676947
324	22	0	A_32_P50603	NM_001105519	0,801604635	7,312167177	2,947142005	0,027571867	0,843980537	-4,248771806
405	79	0	A_23_P930	NM_001077628	-0,63373996	9,030361201	-2,946574587	0,027591459	0,843980537	-4,248848227
125	15	0	A_24_P919063	A_24_P919063	0,516891823	5,436832082	2,945799302	0,027618253	0,843980537	-4,248952675
164	13	0	A_24_P726495	A_24_P726495	0,752521006	5,868477532	2,944538567	0,027661885	0,843980537	-4,249122601
87	60	0	A_32_P58614	NM_020802	-0,675216	5,142730719	-2,944152297	0,027675268	0,843980537	-4,249174683
516	52	0	A_23_P62652	NM_003037	0,594282479	5,720284589	2,944003265	0,027680433	0,843980537	-4,24919478
512	40	0	A_24_P290692	NM_001145079	1,057351672	5,018924008	2,9417793	0,027757642	0,843980537	-4,249494835
495	57	0	A_24_P170067	A_24_P170067	0,764455259	6,286818165	2,941620971	0,027763147	0,843980537	-4,249516207
299	19	0	A_24_P72518	NM_015328	0,424922379	8,794415269	2,941210953	0,02777741	0,843980537	-4,249571563
118	72	0	A_23_P212672	NM_178130	-0,681893771	6,668737717	-2,941202139	0,027777717	0,843980537	-4,249572753
183	80	0	A_24_P99005	A_24_P99005	-0,852929458	6,190504719	-2,940682001	0,027795822	0,843980537	-4,24964299
509	37	0	A_32_P87491	U79293	0,475079684	6,310358199	2,939664075	0,027831293	0,843980537	-4,249780492
246	14	0	A_23_P79094	NM_006865	0,43916063	6,561170511	2,939652882	0,027831683	0,843980537	-4,249782004
14	45	0	A_23_P11070	NM_032553	-0,609621663	5,313719382	-2,939548381	0,027835327	0,843980537	-4,249796124
109	28	0	A_32_P810302	BC032019	0,845884298	5,697433309	2,93840693	0,027875167	0,843980537	-4,249950394
10	77	0	A_24_P942017	NR_034127	-0,442298299	7,355593111	-2,938333573	0,02787773	0,843980537	-4,249960311
190	77	0	A_24_P465697	ENST00000446333	-0,792477127	6,473454033	-2,937665751	0,02790107	0,843980537	-4,250050608
39	72	0	A_24_P289854	A_24_P289854	-0,534039707	5,787162979	-2,937143497	0,027919337	0,843980537	-4,250121241
118	80	0	A_23_P102634	NM_080827	-1,049580794	5,864746437	-2,936707979	0,027934581	0,843980537	-4,250180155
134	51	0	A_24_P287189	NM_019009	-0,756025821	5,638723009	-2,93627491	0,027949748	0,843980537	-4,25023875
225	31	0	A_23_P51002	NM_176825	0,62626901	6,439217284	2,93620745	0,027952111	0,843980537	-4,250247878
250	12	0	A_23_P139986	NM_080818	0,453679444	5,824272237	2,935925232	0,027962001	0,843980537	-4,25028607
46	32	0	A_24_P193570	NM_016284	-0,45554836	9,934758737	-2,934865636	0,027999165	0,843980537	-4,250429503
117	48	0	A_23_P105409	NM_006301	-0,434947345	8,494391743	-2,933358089	0,028052135	0,843980537	-4,25063369
235	11	0	A_23_P378288	NM_022465	0,685983397	8,408037766	2,93274764	0,028073615	0,843980537	-4,250716409
239	47	0	A_24_P316305	ENST00000156471	0,554524149	8,3629942	2,931862799	0,028104781	0,843980537	-4,25083635
384	28	0	A_32_P108066	NM_000339	0,684701563	7,559304919	2,930841534	0,0281408	0,843980537	-4,250974842
116	1	0	A_23_P1998	NM_001164	-0,414417275	9,010693885	-2,930500457	0,028152841	0,843980537	-4,251021109
289	51	0	A_23_P30240	AF116675	-0,935309204	6,294130772	-2,930244581	0,028161878	0,843980537	-4,251055822
42	69	0	A_24_P932760	ENST00000553286	-0,50411612	7,511698616	-2,929590225	0,028185002	0,843980537	-4,251144614
110	83	0	A_32_P133780	ENST00000451149	-0,982261717	6,879890924	-2,929293011	0,028195512	0,843980537	-4,251184953
184	25	0	A_23_P142096	NM_001506	0,480125431	5,866460112	2,929094994	0,028202516	0,843980537	-4,251211831
51	68	0	A_24_P217330	NM_032806	-0,397892068	8,434382139	-2,928905009	0,028209238	0,843980537	-4,251237621
175	15	0	A_23_P26404	NM_005752	0,674602051	5,992740531	2,928783126	0,028213552	0,843980537	-4,251254168
464	85	0	A_24_P402825	NM_018398	-0,629380361	6,402452608	-2,928424558	0,028226246	0,843980537	-4,251302852
9	9	0	A_24_P489399	A_24_P489399	0,625916179	7,586353752	2,928280312	0,028231354	0,843980537	-4,251322438
190	70	0	A_23_P158481	NM_000769	-0,74227507	6,095066695	-2,928269696	0,02823173	0,843980537	-4,25132388
420	11	0	A_23_P147578	AK096677	-0,534241788	5,789035867	-2,928254497	0,028232269	0,843980537	-4,251325944
53	75	0	A_24_P49427	NM_020864	-0,753896796	6,064906079	-2,928080149	0,028238445	0,843980537	-4,25134962
188	77	0	A_24_P15658	NM_198273	-0,580402986	5,805541171	-2,927423291	0,028261726	0,843980537	-4,251438837
517	12	0	A_24_P686975	NM_173822	-0,488472018	5,197327527	-2,926572008	0,02829193	0,843980537	-4,251554499
132	65	0	A_24_P264154	NM_014157	-0,629628387	7,00010845	-2,926402938	0,028297932	0,843980537	-4,251577476
502	19	0	A_23_P210554	NM_006038	-0,403815028	6,962443349	-2,926258745	0,028303053	0,843980537	-4,251597073
103	24	0	A_24_P133288	X97675	0,769067303	6,649706829	2,925128396	0,02834323	0,843980537	-4,25175074
477	84	0	A_24_P329229	NM_153033	-0,386663195	6,292418734	-2,923358331	0,028406269	0,843980537	-4,251991528

432	31	0	A_24_P918561	A_24_P918561	1,042103285	5,892679178	2,922155316	0,028449201	0,843980537	-4,252155285
236	22	0	A_23_P62446	NM_001001877	0,559334221	5,422005617	2,922145247	0,02844956	0,843980537	-4,252156656
146	80	0	A_32_P982	TCONS_00008108	-0,769892956	5,750719622	-2,921483091	0,028473221	0,843980537	-4,252246828
365	14	0	A_23_P148629	NM_004681	0,427625881	8,886390392	2,92148196	0,028473262	0,843980537	-4,252246982
350	1	0	A_24_P237936	NM_175769	0,639618686	6,639627369	2,920879864	0,028494795	0,843980537	-4,252328998
358	50	0	A_23_P27528	NM_000764	0,630816265	6,329453926	2,920869542	0,028495164	0,843980537	-4,252330404
156	75	0	A_24_P406245	BC010535	-0,40914742	9,024394026	-2,920151831	0,028520856	0,843980537	-4,252428197
458	6	0	A_24_P65941	NR_026812	0,426980524	5,333597511	2,919608174	0,028540334	0,843980537	-4,252502295
146	79	0	A_23_P69699	NM_000909	-0,983983158	6,575319985	-2,919544458	0,028542618	0,843980537	-4,25251098
96	35	0	A_23_P338410	NM_147198	0,575189178	5,719699436	2,918807733	0,028569038	0,843980537	-4,252611423
22	17	0	A_23_P387624	NM_001243007	0,875123763	6,030795794	2,918038512	0,028596653	0,843980537	-4,252716332
223	79	0	A_32_P170397	ENST00000309874	-0,540985095	6,856134318	-2,917869056	0,02860274	0,843980537	-4,252739447
376	29	0	A_24_P921832	A_24_P921832	0,62413174	5,913664155	2,916222052	0,028661978	0,843980537	-4,252964206
274	45	0	A_23_P138352	NM_004185	0,439882382	6,57892022	2,913307986	0,028767117	0,843980537	-4,253362273
12	70	0	A_23_P89587	NM_003396	-0,49145346	6,215278462	-2,912898337	0,028781931	0,843980537	-4,253418273
375	10	0	A_23_P29282	NM_014293	-0,542675266	6,577084346	-2,912657125	0,028790658	0,843980537	-4,253451252
142	71	0	A_32_P41070	NM_001017395	-0,477528677	6,842871167	-2,912510373	0,028795968	0,843980537	-4,253471318
137	82	0	A_32_P233278	THC2723346	0,4603955	7,759756924	2,912233722	0,028805983	0,843980537	-4,253509149
112	73	0	A_23_P402899	NM_144975	-0,918047476	5,762315898	-2,911834492	0,028820441	0,843980537	-4,25356375
219	48	0	A_23_P151915	NM_004751	-0,749704609	6,583745464	-2,909353083	0,028910483	0,843980537	-4,253903337
47	78	0	A_24_P322353	NM_024430	-0,901472818	6,745370599	-2,909052624	0,028921407	0,843980537	-4,253944481
87	79	0	A_23_P36458	NM_001167856	-0,848069497	7,074447272	-2,908628799	0,028936823	0,843980537	-4,254002527
273	28	0	A_23_P127781	NM_006552	0,537156813	5,912611925	2,908481659	0,028942177	0,843980537	-4,254022682
91	80	0	A_32_P122468	THC2562062	-0,599738874	6,969371624	-2,908431574	0,028944	0,843980537	-4,254029543
357	64	0	A_24_P933984	A_24_P933984	0,633138463	5,801363539	2,908221011	0,028951665	0,843980537	-4,254058387
442	55	0	A_24_P7121	NM_024677	0,497195875	7,066300699	2,908092389	0,028956347	0,843980537	-4,254076009
521	33	0	A_24_P557274	NR_038962	0,513019959	6,734759916	2,907867292	0,028964545	0,843980537	-4,254106849
388	82	0	A_32_P227657	BX114900	0,480921788	7,629658046	2,902998989	0,029142455	0,843980537	-4,254774601
14	56	0	A_23_P6381	NM_002430	-0,759181376	5,946274059	-2,901789216	0,02918685	0,843980537	-4,254940759
201	79	0	A_23_P310086	NM_152731	-0,746818763	6,44724128	-2,901688182	0,029190561	0,843980537	-4,25495464
55	45	0	A_24_P9883	NM_138368	-0,601423082	5,41529015	-2,901626764	0,029192817	0,843980537	-4,254963078
210	30	0	A_23_P318616	NM_001039029	0,503825365	6,097260681	2,901408431	0,029200839	0,843980537	-4,254993077
300	79	0	A_23_P104445	NM_152309	-0,466167346	5,899886007	-2,900359749	0,029239403	0,843980537	-4,255137205
27	54	0	A_23_P203613	THC2729336	-0,462723248	6,224135338	-2,900277885	0,029242416	0,843980537	-4,255148459
63	10	0	A_23_P152218	NM_001950	-0,584609861	7,956570574	-2,899713802	0,029263184	0,843980537	-4,255226015
75	56	0	A_32_P150676	A_32_P150676	-0,459504892	5,597398516	-2,899456082	0,029272678	0,843980537	-4,255261455
198	79	0	A_24_P927863	ENST00000455661	-0,411894471	5,616102107	-2,89943095	0,029273604	0,843980537	-4,255264912
206	7	0	A_23_P34478	NM_017655	0,430271269	5,998942944	2,898152171	0,029320764	0,843980537	-4,255440825
226	31	0	A_23_P13031	NM_001335	0,695351305	6,431081452	2,898079895	0,029323432	0,843980537	-4,25545077
38	74	0	A_23_P159435	ENST00000390283	-0,661334712	6,787505971	-2,896482901	0,02938245	0,843980537	-4,255670604
450	77	0	A_23_P82839	NM_178819	-0,495292306	8,433906359	-2,896008137	0,02940002	0,843980537	-4,255735987
467	74	0	A_23_P205724	NM_017970	-0,637543696	7,552843113	-2,895505789	0,029418624	0,843980537	-4,255805184
66	63	0	A_23_P156505	NM_002460	-0,544025155	6,532783629	-2,894339364	0,02946187	0,843980537	-4,255965913
93	68	0	A_24_P68991	NM_006168	-0,861828427	6,325739266	-2,893781616	0,029482573	0,843980537	-4,256042798
36	37	0	A_24_P261032	NM_172037	-0,553677538	6,011756162	-2,893759133	0,029483408	0,843980537	-4,256045898
489	65	0	A_23_P142918	NM_153689	0,443627255	7,545464723	2,892990582	0,029511963	0,843980537	-4,256151874
213	46	0	A_23_P155351	NM_000060	-0,436432722	6,158897129	-2,892595578	0,029526651	0,843980537	-4,256206355
398	43	0	A_24_P357405	A_24_P357405	0,41351936	5,527729563	2,891056051	0,029583972	0,843980537	-4,256418785
477	52	0	A_23_P121120	NM_023915	0,40468583	6,209015065	2,890053259	0,029621375	0,843980537	-4,256557231
490	34	0	A_32_P13337	THC2645080	0,498242368	5,615941969	2,889433238	0,029644527	0,843980537	-4,256642862
93	49	0	A_32_P40250	THC2506535	-0,551275275	5,733799113	-2,88899664	0,029660841	0,843980537	-4,256703174
453	25	0	A_23_P163361	NM_000326	0,666372663	5,892910277	2,888134565	0,029693083	0,843980537	-4,256822297
393	4	0	A_32_P116206	NM_001085400	0,521749971	9,586698338	2,887949426	0,029700012	0,843980537	-4,256847885
121	68	0	A_23_P144980	NM_181523	-0,413190378	6,731700549	-2,886723526	0,029745939	0,843980537	-4,257017373
135	81	0	A_32_P232327	A_32_P232327	-0,608865137	6,997728133	-2,886612063	0,029750119	0,843980537	-4,257032787
310	32	0	A_32_P909570	NM_182758	0,431884118	5,03382726	2,886512176	0,029753865	0,843980537	-4,257046602
235	54	0	A_23_P20832	NM_003127	-0,492215272	12,5189072	-2,886384444	0,029758656	0,843980537	-4,257064268
248	76	0	A_23_P144872	NM_000405	-0,43592696	6,864155934	-2,885894193	0,029777053	0,843980537	-4,257132084
191	71	0	A_23_P38696	NM_004948	-0,953354234	6,70989817	-2,885842235	0,029779003	0,843980537	-4,257139272
467	41	0	A_23_P150595	NM_004179	0,443186142	6,300503934	2,883860269	0,029853509	0,843980537	-4,257413587
528	67	0	A_24_P303815	NM_152896	-0,441031804	8,011623624	-2,882733754	0,02989547	0,843980537	-4,257569609
227	32	0	A_24_P922173	A_24_P922173	0,78744411	5,310421311	2,882445615	0,029906813	0,843980537	-4,257609529
332	85	0	A_32_P151087	A_32_P151087	-0,464498996	6,650696143	-2,882207874	0,029915781	0,843980537	-4,25764247
158	85	0	A_32_P235753	BQ060012	-0,779542368	6,630566324	-2,8821106	0,029919451	0,843980537	-4,257655949
505	24	0	A_23_P136589	NM_207189	0,57423844	5,295195292	2,881488511	0,029942935	0,843980537	-4,257742164
399	27	0	A_24_P231104	NM_001003679	0,527469528	6,875885744	2,880804564	0,029968777	0,843980537	-4,25783698
394	37	0	A_23_P254558	NR_026783	0,578242514	5,893994668	2,880482642	0,029980949	0,843980537	-4,257881618
499	7	0	A_24_P6864	NP106540	0,537616167	5,288856318	2,880086958	0,029995917	0,843980537	-4,257936492
172	84	0	A_32_P530933	NM_015617	-0,787797988	6,768376537	-2,879830795	0,030005611	0,843980537	-4,257972022
456	37	0	A_23_P253123	NM_016267	0,467107422	6,121793684	2,879223182	0,03002862	0,843980537	-4,258056316
208	57	0	A_23_P92899	NM_031908	-0,396876002	7,425662979	-2,878626022	0,030051252	0,843980537	-4,25813918
143	67	0	A_32_P215745	A_32_P215745	-0,774570272	5,734331914	-2,877460885	0,030095463	0,843980537	-4,258300922
177	9	0	A_24_P923028	ENST00000566010	0,777812513	6,188768962	2,876657415	0,030125992	0,843980537	-4,258412506
288	39	0	A_24_P485012	BC017563	0,792834353	6,854633285	2,875478966	0,03017083	0,843980537	-4,258576237
39	81	0	A_23_P84496	NM_012418	-0,824309133	6,378383902	-2,875396244	0,03017398	0,843980537	-4,258587734
376	17	0	A_23_P74928	NM_001531	0,70100636	6,008842707	2,874787581	0,03019717	0,843980537	-4,258672336
143	1	0	A_24_P84719	TCONS_l2_00019431	0,668619021	6,040256532	2,874565234	0,030205646	0,843980537	-4,258703247
238	14	0	A_23_P82929	NM_002514	0,602469993	6,434756522	2,874177846	0,03022042	0,843980537	-4,25875711
233	71	0	A_32_P150012	NM_017780	0,554426055	8,454095802	2,87355144	0,030244326	0,843980537	-4,258844225
351	63	0	A_24_P683583	A_24_P683583	0,491896801	5,690956381	2,873516653	0,030245654	0,843980537	-4,258849064
158	13	0	A_32_P82515	A_32_P82515	0,652296532	7,455177606	2,873371254	0,030251206	0,843980537	-4,258869288
439	34	0	A_32_P45309	ENST00000569751	0,431877131	5,494895372	2,872803832	0,030272884	0,843980537	-4,258948227
265	58	0	A_32_P142815	NM_001692	-0,548690904	7,389970583	-2,872696716	0,030276979	0,843980537	-4,258963131
132	52	0	A_24_P919585	H24400	-0,405421876	5,789916793	-2,872297495	0,030292243	0,843980537	-4,259018685
108	68	0	A_24_P371425	NM_032166	-0,521923154	7,362076327	-2,872227521	0,030294919	0,843980537	-4,259028423
460	41	0	A_23_P402778	NM_138700	0,597328835	6,5311168	2,872076563	0,030300694	0,843980537	-4,259049432
147	9	0	A_23_P379327	NM_001040450	0,423688031	8,303813822	2,871927067	0,030306414	0,843980537	-4,25907024
491	29	0	A_32_P23308	NR_026945	0,867971182	5,423339882	2,871285693	0,030330967	0,843980537	-4,259159525
26	56	0	A_24_P225189	NM_014801	-0,488683402	6,656356574	-2,871217679	0,030333572	0,843980537	-4,259168994
169	73	0	A_24_P316506	ENST00000419257	-0,420167052	6,728298955	-2,869047829	0,030416808	0,843980537	-4,25947125
283	73	0	A_32_P190648	BX096698	0,660175551	9,404312143	2,868385976	0,030442246	0,843980537	-4,259563502
307	17	0	A_24_P212819	AK057884	0,604416298	7,29969665	2,867642564	0,030470846	0,843980537	-4,259667153
137	7	0	A_32_P230523	NM_173526	0,46822492	5,930053336	2,866375236	0,03051967	0,843980537	-4,25984393
465	71	0	A_23_P144202	NM_021937	-0,709325882	6,711705367	-2,86598544	0,030534704	0,843980537	-4,259898322

472	82	0	A_23_P321466	NM_080662	-0,671430385	6,841779762	-2,86421289	0,030603172	0,843980537	-4,260145777
153	24	0	A_24_P833067	ENST00000605164	0,527413574	5,945440979	2,863422895	0,03063374	0,843980537	-4,260256124
455	45	0	A_24_P228875	NM_004840	-0,689856118	6,136359609	-2,863214188	0,030641821	0,843980537	-4,260285283
18	6	0	A_23_P210100	NM_019885	0,764050615	5,596928567	2,86277401	0,030658873	0,843980537	-4,26034679
39	78	0	A_32_P196142	ENST00000586949	-0,780321636	6,255270337	-2,862214219	0,030680574	0,843980537	-4,260425028
397	20	0	A_32_P174258	THC2641587	0,632823181	8,672709338	2,862136702	0,03068358	0,843980537	-4,260435864
144	47	0	A_23_P200222	NM_033300	0,680973428	8,208779247	2,861232192	0,030718682	0,843980537	-4,260562325
218	15	0	A_23_P213137	NM_032622	0,627746529	5,756247174	2,860767903	0,030736718	0,843980537	-4,260627257
166	14	0	A_24_P891042	A_24_P891042	0,676530193	6,253290501	2,85957408	0,030783145	0,843980537	-4,260794277
13	39	0	A_23_P28246	NM_144712	-0,940979727	6,200260757	-2,858474022	0,030825993	0,843980537	-4,260948256
502	84	0	A_24_P222139	AK025047	-0,446370778	6,027258993	-2,858234284	0,030835339	0,843980537	-4,260981823
336	8	0	A_24_P332806	NM_005540	0,584181176	5,871092582	2,857478495	0,030864825	0,843980537	-4,261087667
525	42	0	A_24_P96171	NM_030578	0,48529238	8,938945866	2,856205393	0,030914562	0,843980537	-4,261266037
467	18	0	A_23_P207245	NM_031898	0,478778939	5,904201963	2,855983206	0,030923251	0,843980537	-4,261297177
36	81	0	A_23_P419714	NM_001018072	-0,733641815	7,129679676	-2,855920384	0,030925709	0,843980537	-4,261305982
442	23	0	A_32_P101195	A_32_P101195	0,39869558	10,36971774	2,854404431	0,030985071	0,843980537	-4,261518533
445	44	0	A_23_P42588	NM_018384	0,877651264	5,206985366	2,854011628	0,031000472	0,843980537	-4,26157363
245	9	0	A_24_P335263	NM_199040	0,505378144	9,227841969	2,853749813	0,031010743	0,843980537	-4,261610359
309	37	0	A_23_P431430	ENST00000378252	0,585083199	6,45354944	2,853301899	0,031028322	0,843980537	-4,261673205
109	12	0	A_23_P208238	NR_023311	0,425623756	6,773086948	2,852766005	0,031049368	0,843980537	-4,261748412
136	29	0	A_32_P221507	NR_037694	0,700868113	6,131758623	2,852413788	0,031063209	0,843980537	-4,261797851
168	18	0	A_24_P585902	AK074346	0,534410038	6,41266721	2,852322574	0,031066795	0,843980537	-4,261810656
330	85	0	A_32_P153937	THC2549728	-0,574555568	6,778146883	-2,852315591	0,031067069	0,843980537	-4,261811636
111	27	0	A_24_P4170	NM_020361	0,649754202	5,148932244	2,851922417	0,03108253	0,843980537	-4,261866835
185	75	0	A_24_P152929	AK025112	-0,75688617	6,785973102	-2,849774901	0,031167123	0,843980537	-4,262168501
207	81	0	A_24_P244575	NM_001143819	-0,696457207	7,19420345	-2,849696755	0,031170206	0,843980537	-4,262179484
138	66	0	A_23_P125705	NM_021963	-0,77539914	5,261556872	-2,84887653	0,031202586	0,843980537	-4,26229478
176	44	0	A_24_P461389	A_24_P461389	0,431999197	8,997228356	2,847939876	0,031239606	0,843980537	-4,262426494
289	83	0	A_24_P888941	A_24_P888941	-0,846165241	6,458837944	-2,847309399	0,031264552	0,843980537	-4,262515182
421	9	0	A_24_P355006	NM_021723	0,399370516	9,368766452	2,847234946	0,031267499	0,843980537	-4,262525657
181	77	0	A_24_P363259	NM_033181	-0,772059111	6,504318199	-2,845734941	0,031326943	0,843980537	-4,262736765
112	72	0	A_24_P936419	THC2781374	-0,47180403	7,009253673	-2,845190952	0,031348531	0,843980537	-4,262813359
176	71	0	A_23_P8297	NM_198081	-0,423896459	6,336826196	-2,844773219	0,031365119	0,843980537	-4,262872188
231	33	0	A_24_P247408	AY129010	0,677930323	7,339435885	2,844610484	0,031371584	0,843980537	-4,262895109
66	70	0	A_23_P14612	NM_002009	-0,985519939	6,086783357	-2,844328425	0,031382793	0,843980537	-4,26293484
230	1	0	A_23_P30254	NM_006622	0,477509968	8,685127311	2,843632581	0,031410463	0,843980537	-4,263032879
162	15	0	A_23_P332713	NM_015174	0,518237741	5,364694481	2,843372104	0,031420828	0,843980537	-4,263069585
143	64	0	A_24_P538839	BC020376	-0,59734786	5,971455372	-2,842508803	0,031455207	0,843980537	-4,263191272
309	41	0	A_32_P72447	NM_014501	-0,404181267	13,85728737	-2,842021786	0,03147462	0,843980537	-4,263259939
85	22	0	A_23_P39215	NM_001105570	0,539929673	8,401304604	2,841867187	0,031480785	0,843980537	-4,26328174
454	40	0	A_32_P122860	ENST00000544421	0,816458967	6,393068478	2,839429236	0,031578178	0,843980537	-4,263625723
193	85	0	A_24_P272313	NM_207362	-0,461892666	7,100796497	-2,839359527	0,031580967	0,843980537	-4,263635564
105	37	0	A_24_P173823	NM_002585	0,402124613	10,20803099	2,83928308	0,031584027	0,843980537	-4,263646356
133	1	0	A_24_P919595	A_24_P919595	0,628664429	6,5638783	2,839107584	0,031591052	0,843980537	-4,263671133
171	29	0	A_32_P217853	XR_242680	0,484741492	5,821032059	2,838438605	0,031617846	0,843980537	-4,2637656
101	26	0	A_32_P500271	NM_001098627	0,433339694	9,031229417	2,836816009	0,031682937	0,843980537	-4,26399484
189	23	0	A_24_P621312	A_24_P621312	0,39729264	6,611654814	2,836700673	0,031687569	0,843980537	-4,264011141
24	68	0	A_24_P463779	ENST00000450527	-0,696794521	5,015268644	-2,836485935	0,031696195	0,843980537	-4,264041492
139	1	0	A_24_P234391	NM_025202	0,501158505	6,663371324	2,836182333	0,031708396	0,843980537	-4,264084409
83	83	0	A_23_P93629	NM_015905	0,421868169	12,57627896	2,836142049	0,031710015	0,843980537	-4,264090104
486	81	0	A_24_P314640	NM_153487	-0,497108351	6,566426354	-2,835438539	0,031738308	0,843980537	-4,264189575
72	66	0	A_23_P106559	NM_001193388	-0,67194335	7,032539425	-2,835005203	0,03175575	0,843980537	-4,26425086
300	23	0	A_24_P943411	A_24_P943411	0,528511102	5,784135334	2,834360652	0,031781711	0,843980537	-4,264342038
224	81	0	A_23_P49532	NM_014906	-0,77476203	6,473060212	-2,834269978	0,031785365	0,843980537	-4,264354867
402	33	0	A_23_P91168	NM_144710	0,596559829	7,668386442	2,834086187	0,031792773	0,843980537	-4,264380872
40	68	0	A_23_P107974	NM_000511	-0,662810686	6,068464386	-2,833029089	0,031835417	0,843980537	-4,264530482
484	83	0	A_24_P10444	NM_173623	-0,54415958	5,388500978	-2,832806736	0,031844395	0,843980537	-4,26456196
289	38	0	A_23_P341312	NM_198969	-0,454677885	8,920030214	-2,832045313	0,03187516	0,843980537	-4,264669776
308	74	0	A_24_P63827	NM_005494	-0,581979747	6,857987994	-2,831479041	0,03189806	0,843980537	-4,264749982
260	32	0	A_23_P216361	NM_021110	0,495735146	6,386451938	2,831049974	0,031915424	0,843980537	-4,264810768
218	30	0	A_23_P319565	NM_170753	0,498882479	7,637048764	2,831022009	0,031916556	0,843980537	-4,26481473
390	84	0	A_23_P86610	NM_014951	0,413850112	7,413808202	2,830758923	0,031927208	0,843980537	-4,264852007
202	77	0	A_23_P161231	NM_030971	-0,384267984	7,83490242	-2,828368287	0,032024181	0,843980537	-4,265190937
459	47	0	A_24_P306594	ENST00000433342	0,377290191	6,123756745	2,827356957	0,0320653	0,843980537	-4,265334422
213	1	0	A_23_P205828	NM_003257	0,453222989	8,49317592	2,826623499	0,032095158	0,843980537	-4,265438523
197	21	0	A_23_P103349	NM_022375	0,616800966	5,966652766	2,825801939	0,032128637	0,843980537	-4,265555167
222	16	0	A_24_P928819	A_24_P928819	0,578729146	6,221545715	2,825330516	0,032147866	0,843980537	-4,265622118
248	4	0	A_23_P331943	NM_020823	0,664803207	8,999824899	2,82521289	0,032152665	0,843980537	-4,265638825
78	60	0	A_24_P358554	NM_198427	-0,654954874	6,357545819	-2,824248662	0,032192039	0,843980537	-4,265775813
184	54	0	A_32_P141969	ENST00000394480	0,481624752	6,854928131	2,824242797	0,032192279	0,843980537	-4,265776647
320	3	0	A_23_P163306	NM_032866	0,50683226	7,825193787	2,823941508	0,032204593	0,843980537	-4,265819463
193	72	0	A_24_P345822	NM_006070	-0,389419899	10,80883676	-2,821852833	0,032290099	0,843980537	-4,266116436
155	60	0	A_32_P207147	ENST00000422438	-0,488294262	6,192390061	-2,821413579	0,032308113	0,843980537	-4,266178924
86	21	0	A_23_P335039	NM_133474	0,417155543	9,602884273	2,821279468	0,032313615	0,843980537	-4,266198005
50	56	0	A_32_P141238	NM_001278597	-0,439449486	5,772547899	-2,821010674	0,032324646	0,843980537	-4,266236252
140	78	0	A_24_P24724	A_24_P24724	0,408962638	8,302942247	2,820913942	0,032328616	0,843980537	-4,266250017
320	45	0	A_23_P433753	NM_212472	-0,369696898	10,36508515	-2,819946275	0,032368366	0,843980537	-4,266387749
165	70	0	A_24_P557479	NM_017523	-0,540470365	6,237406842	-2,819624547	0,032381593	0,843980537	-4,266433555
433	1	0	A_23_P112397	NM_018998	-0,411924429	10,4773558	-2,819579322	0,032383453	0,843980537	-4,266439994
12	75	0	A_24_P457226	TCONS_00024099	-1,18118234	6,121405556	-2,819506134	0,032386463	0,843980537	-4,266450415
205	73	0	A_24_P483956	BM913108	-0,70147652	7,330858748	-2,81910394	0,03240301	0,843980537	-4,266507689
257	61	0	A_24_P367571	NM_017794	-0,460172971	7,223562556	-2,817536863	0,03246757	0,843980537	-4,266730941
254	68	0	A_24_P88583	ENST00000473274	-0,386070575	6,211859256	-2,817280638	0,032478139	0,843980537	-4,266767458
74	69	0	A_23_P1912	NM_207341	-0,659251957	6,788040952	-2,817263579	0,032478843	0,843980537	-4,266769889
482	84	0	A_24_P935919	ENST00000513816	-0,807736674	6,200507031	-2,816548892	0,032508343	0,843980537	-4,266871768
313	18	0	A_24_P51037	NM_001201335	0,449531717	8,714317502	2,815291305	0,032560324	0,843980537	-4,267051114
268	35	0	A_23_P22433	NM_006915	0,416486024	9,75458882	2,813754267	0,032623979	0,843980537	-4,267270445
301	78	0	A_23_P53826	NM_033132	-1,227532107	6,134509338	-2,813353375	0,032640604	0,843980537	-4,267327675
173	84	0	A_24_P920157	A_24_P920157	-0,861942974	6,580341862	-2,813059642	0,032652791	0,843980537	-4,267369614
324	11	0	A_23_P409438	NM_172138	0,644838433	6,540803688	2,812802676	0,032663456	0,843980537	-4,267406307
369	48	0	A_23_P425967	NM_175064	0,571293219	5,532801498	2,812411833	0,032679685	0,843980537	-4,267462125
290	85	0	A_24_P187119	AK098491	-0,456369076	8,082462596	-2,812244762	0,032686625	0,843980537	-4,267485989
308	5	0	A_23_P253622	AK024934	0,414512278	8,918443747	2,811295177	0,032726101	0,843980537	-4,267621652
205	21	0	A_24_P914817	NM_013255	0,429191079	7,540332055	2,810313122	0,03276698	0,843980537	-4,267762014

341	43	0	A_23_P155624	NM_004068	-0,43267558	10,90380994	-2,809568881	0,032797998	0,843980537	-4,267868424
130	15	0	A_24_P930963	NR_036442	0,557698016	6,17659315	2,809067507	0,032818911	0,843980537	-4,26794013
147	67	0	A_24_P303091	NM_001565	-0,893712737	6,444027777	-2,809012183	0,03282122	0,843980537	-4,267948043
396	84	0	A_24_P403561	NM_002334	1,011162541	8,539599698	2,808611212	0,032837957	0,843980537	-4,268005401
91	78	0	A_24_P584992	A_24_P584992	-0,394196465	10,06764617	-2,806903185	0,032909356	0,843980537	-4,268249843
78	63	0	A_32_P63886	CU674743	-0,620029557	6,506748184	-2,806537453	0,032924667	0,843980537	-4,268302208
276	22	0	A_32_P11579	NM_021032	0,462858794	6,206425097	2,806475585	0,032927258	0,843980537	-4,268311066
467	7	0	A_23_P61987	NM_025268	0,524423829	9,052831708	2,806473417	0,032927348	0,843980537	-4,268311377
60	75	0	A_23_P49192	NM_198061	-0,451538582	7,946879069	-2,806263686	0,032936132	0,843980537	-4,26834141
200	28	0	A_24_P925455	A_24_P925455	0,442468924	5,502191216	2,806237931	0,032937211	0,843980537	-4,268345098
149	2	0	A_24_P373152	NM_021914	0,587316782	7,130346683	2,806165801	0,032940233	0,843980537	-4,268355428
250	17	0	A_32_P214178	NR_026960	0,868001183	6,131017248	2,80581518	0,032954925	0,843980537	-4,268405645
168	15	0	A_24_P110827	AK096522	0,552356274	6,529535478	2,805491267	0,032968505	0,843980537	-4,268452043
427	53	0	A_23_P107578	AK022058	0,576097817	6,898867728	2,804117144	0,033026181	0,843980537	-4,268648949
206	12	0	A_23_P93282	NM_003535	0,506416683	7,146134142	2,803814152	0,033038914	0,843980537	-4,268692382
253	48	0	A_24_P311036	NM_002479	0,631524267	7,020792712	2,803784222	0,033040172	0,843980537	-4,268696672
123	85	0	A_24_P557232	ENST00000423311	0,72930596	9,122664685	2,80345794	0,033053889	0,843980537	-4,268743451
84	60	0	A_24_P143686	NM_001080394	-0,460892999	9,226782554	-2,802004375	0,033115074	0,843980537	-4,268951926
180	73	0	A_32_P50431	NM_003932	-0,845595326	5,060266436	-2,801282532	0,033145505	0,843980537	-4,269055503
109	77	0	A_24_P85606	A_24_P85606	-0,636652768	5,3815057	-2,800290563	0,033187372	0,843980537	-4,269197894
90	85	0	A_24_P59278	AF068286	-0,91294071	6,610543728	-2,799442046	0,033223231	0,843980537	-4,26931974
167	28	0	A_24_P149689	NM_147180	0,576123853	6,148611899	2,799330239	0,033227959	0,843980537	-4,269335799
151	26	0	A_24_P46946	NM_022454	0,494468987	8,013246882	2,79930553	0,033229004	0,843980537	-4,269339348
108	29	0	A_24_P1929	NM_007010	0,406892131	9,502409894	2,799284982	0,033229873	0,843980537	-4,2693423
240	13	0	A_24_P170569	NM_017639	0,614377529	5,751542134	2,799170486	0,033234716	0,843980537	-4,269358746
292	24	0	A_23_P107048	ENST00000580169	0,712270115	7,390466361	2,798838446	0,033248765	0,843980537	-4,269406445
346	81	0	A_24_P187448	A_24_P187448	-0,862511768	6,791260584	-2,797770027	0,033294014	0,843980537	-4,269559972
427	77	0	A_23_P15369	NM_174892	-0,61011346	6,898395236	-2,797666716	0,033298393	0,843980537	-4,269574821
525	58	0	A_23_P141394	NM_017983	0,526911846	9,931745563	2,797129146	0,033321188	0,843980537	-4,269652098
315	5	0	A_24_P646573	THC2720182	0,540623408	5,723710213	2,797079144	0,033323309	0,843980537	-4,269659287
94	83	0	A_32_P224855	NM_024646	-0,726024809	6,224725963	-2,796304119	0,033356206	0,843980537	-4,269770732
504	83	0	A_32_P177267	NM_001034172	-0,488694283	5,747083212	-2,796028279	0,033367923	0,843980537	-4,269810406
430	78	0	A_24_P524164	ENST00000450890	-0,544150192	5,97105797	-2,795153719	0,033405101	0,843980537	-4,269936223
220	32	0	A_32_P118461	AW328034	0,41909307	5,964397964	2,794837018	0,033418575	0,843980537	-4,269981797
163	24	0	A_24_P341089	NR_015424	0,456650564	9,032464822	2,794526974	0,033431772	0,843980537	-4,270026418
104	4	0	A_24_P666553	TCONS_00001027	0,581899663	5,327542127	2,794424009	0,033436156	0,843980537	-4,270041238
251	49	0	A_23_P123794	NM_001807	0,644306802	6,845500533	2,794394805	0,033437399	0,843980537	-4,270045442
103	63	0	A_24_P215352	NM_145040	-0,432942557	5,981877291	-2,794197673	0,033445794	0,843980537	-4,270073818
114	72	0	A_23_P137797	NM_001035	-0,828411258	6,383411938	-2,79394417	0,033456593	0,843980537	-4,270110311
423	13	0	A_24_P244096	NM_001002762	0,510323985	7,492549603	2,79381382	0,033462148	0,843980537	-4,270129078
495	80	0	A_32_P221552	A_32_P221552	0,376646587	8,650968469	2,793456414	0,033477382	0,843980537	-4,270180539
88	45	0	A_24_P59494	NM_020367	-0,565070181	5,856591998	-2,793146965	0,033490579	0,843980537	-4,270225101
65	56	0	A_24_P91985	NM_170710	-0,652963218	4,720265286	-2,792777159	0,033506357	0,843980537	-4,270278362
10	43	0	A_23_P87664	NM_014706	-0,393195509	9,837470336	-2,79180354	0,033547935	0,843980537	-4,270418629
178	18	0	A_23_P411235	NM_152460	0,969679509	5,639830291	2,791688409	0,033552855	0,843980537	-4,27043522
174	85	0	A_24_P522267	DA383986	-0,607320378	6,440911611	-2,791191021	0,033574121	0,843980537	-4,270506903
205	76	0	A_23_P349127	NM_138477	-0,424463878	7,300807814	-2,790201742	0,033616461	0,843980537	-4,270649524
18	4	0	A_23_P6201	NM_198996	0,665468488	6,27928409	2,790090147	0,033621241	0,843980537	-4,270665616
15	57	0	A_23_P72668	NM_004657	-0,468628783	5,951141549	-2,789813532	0,033633092	0,843980537	-4,270705507
282	14	0	A_24_P76267	NM_005265	-0,408091604	6,535516246	-2,789695612	0,033638145	0,843980537	-4,270722514
322	7	0	A_23_P66011	NM_019065	0,924144775	7,301203613	2,789488498	0,033647023	0,843980537	-4,270752387
34	73	0	A_32_P134931	ENST00000554678	-0,536677069	6,417502443	-2,788483564	0,033690135	0,843980537	-4,27089737
286	15	0	A_23_P214176	NM_133493	0,656877673	5,362067035	2,788339056	0,033696339	0,843980537	-4,270918224
86	73	0	A_23_P58835	NM_005242	-0,648494645	5,584922534	-2,788005572	0,033710662	0,843980537	-4,270966353
232	1	0	A_24_P604665	AF203728	0,411834749	6,39346613	2,787988128	0,033711411	0,843980537	-4,270968871
162	81	0	A_23_P65845	A_23_P65845	-0,574853427	5,312559557	-2,787485972	0,033732991	0,843980537	-4,271041357
198	74	0	A_24_P336137	NM_032561	-0,450681475	7,567419976	-2,787150407	0,03374742	0,843980537	-4,271089804
345	21	0	A_32_P515431	XR_250705	0,930940188	6,844647287	2,787083552	0,033750296	0,843980537	-4,271099457
107	72	0	A_24_P724807	A_24_P724807	-0,49714628	6,626593825	-2,786132401	0,033791234	0,843980537	-4,271236821
202	81	0	A_24_P200420	NM_014331	-0,720840299	5,495050134	-2,784218467	0,033873776	0,843980537	-4,271513398
108	78	0	A_24_P7974	AF161369	-0,684314203	7,001820723	-2,783637648	0,033898868	0,843980537	-4,271597376
225	14	0	A_24_P140057	NM_020868	0,73043428	5,896404178	2,782325958	0,033955609	0,843980537	-4,271787102
93	71	0	A_24_P85400	ENST00000471826	-0,896890689	6,117759986	-2,782061811	0,033967048	0,843980537	-4,271825322
168	66	0	A_23_P103877	NM_001010847	-0,388759159	6,0723949	-2,78192304	0,033973059	0,843980537	-4,271845403
188	85	0	A_23_P416289	NM_001037954	-0,43603916	8,27635449	-2,781738061	0,033981073	0,843980537	-4,271872172
171	71	0	A_32_P10623	A_32_P10623	-0,930087044	6,16649575	-2,781579214	0,033987957	0,843980537	-4,271895161
153	67	0	A_24_P7192	NM_032446	-0,684354445	6,114707444	-2,780884758	0,034018071	0,843980537	-4,271995685
253	46	0	A_32_P212406	CA437634	0,553645944	7,309180651	2,780106694	0,034051844	0,843980537	-4,272108346
9	70	0	A_23_P200707	NM_018208	-0,625097489	6,207677858	-2,77919794	0,034091336	0,843980537	-4,272239979
318	8	0	A_32_P225816	NM_022114	0,635083438	6,048869843	2,778514442	0,034121071	0,843980537	-4,272339016
100	74	0	A_24_P303052	NM_013261	-0,438393483	6,171227147	-2,778263272	0,034132005	0,843980537	-4,272375418
193	58	0	A_24_P118271	A_24_P118271	-0,417541913	7,181018911	-2,777496516	0,034165408	0,843980537	-4,272486566
106	74	0	A_24_P256764	NM_005964	-0,897119302	7,079386928	-2,777476106	0,034166298	0,843980537	-4,272489525
127	19	0	A_32_P6164	CU693307	0,626556434	4,992754328	2,777083775	0,034183404	0,843980537	-4,272546412
497	55	0	A_24_P51805	NM_022461	0,493606455	7,670753518	2,776982637	0,034187815	0,843980537	-4,272561078
194	56	0	A_32_P232192	NM_001257281	-0,636042026	7,259178498	-2,776079611	0,034227227	0,843980537	-4,272692055
486	53	0	A_23_P35977	NM_024791	0,682114237	5,803686332	2,775491513	0,034252921	0,843980537	-4,272777381
96	70	0	A_23_P397566	NM_139013	-0,465139939	6,679665028	-2,775414658	0,034256281	0,843980537	-4,272788534
370	73	0	A_24_P168232	NM_030576	-0,438282883	8,868588812	-2,775401604	0,034256851	0,843980537	-4,272790428
516	77	0	A_23_P62099	NM_016249	-0,612387785	5,103308205	-2,775322112	0,034260327	0,843980537	-4,272801964
65	82	0	A_32_P211117	ENST00000459979	-0,946010236	6,145050373	-2,775260211	0,034263033	0,843980537	-4,272810946
218	11	0	A_32_P102935	NM_001008779	0,641207352	6,667645836	2,773411768	0,034343954	0,843980537	-4,273079301
425	44	0	A_32_P172534	BM998048	0,426860287	5,077774358	2,771773486	0,034415847	0,843980537	-4,273317321
207	1	0	A_23_P66180	NM_006539	0,52613476	6,132889349	2,770864051	0,034455827	0,843980537	-4,273449522
270	41	0	A_32_P114117	ENST00000443870	-0,387537651	7,833085894	-2,770268648	0,034482029	0,843980537	-4,273536101
120	81	0	A_32_P213330	NM_001177693	-0,472857483	8,608917039	-2,770200439	0,034485032	0,843980537	-4,273546021
66	43	0	A_32_P507580	BC035147	-0,470103827	9,39186277	-2,770162027	0,034486723	0,843980537	-4,273551607
179	81	0	A_23_P75609	NM_014956	-0,729197393	8,990110483	-2,769652799	0,034509153	0,843980537	-4,273625676
165	72	0	A_24_P212860	NM_016481	-0,569555337	6,99053471	-2,76919012	0,034529546	0,843980537	-4,273692988
330	2	0	A_23_P144208	ENST00000394191	0,983909896	6,015971561	2,768605078	0,034555351	0,843980537	-4,273778121
105	79	0	A_24_P357535	NM_001005190	-0,606866279	6,120554795	-2,768436612	0,034562786	0,843980537	-4,27380264
205	65	0	A_24_P84970	A_24_P84970	-0,401259952	6,393505627	-2,768275732	0,034569888	0,843980537	-4,273826056
187	63	0	A_24_P944478	A_24_P944478	-0,608538412	5,316404156	-2,767663196	0,03459694	0,843980537	-4,273915225
365	1	0	A_23_P333150	ENST00000559819	0,377712675	6,740880276	2,767159247	0,034619214	0,843980537	-4,273988604

141	75	0	A_32_P138933	A_32_P138933	-1,109172269	6,28228466	-2,766454502	0,03465039	0,843980537	-4,274091247
196	80	0	A_23_P305292	NR_027180	-0,479209848	6,921995319	-2,766344147	0,034655274	0,843980537	-4,274107322
17	61	0	A_23_P168909	NM_012082	-0,770305686	6,102261766	-2,765306002	0,03470126	0,843980537	-4,274258587
131	5	0	A_24_P937231	ENST00000426841	0,755178859	5,141206804	2,764688575	0,034728641	0,843980537	-4,274348582
70	85	0	A_24_P324733	AF274940	-1,05158213	6,175817483	-2,764552964	0,034734658	0,843980537	-4,274368351
183	68	0	A_24_P155502	NM_005538	-0,463389498	6,597084639	-2,764002141	0,03475911	0,843980537	-4,274448663
507	76	0	A_24_P940365	NM_001145080	-0,549508006	5,919401299	-2,762591677	0,034821807	0,843980537	-4,274654398
496	26	0	A_32_P223464	NM_213608	0,408271015	6,069325436	2,762341193	0,034832954	0,843980537	-4,274690947
404	82	0	A_24_P623782	NM_001005217	-0,430778985	5,938123598	-2,761793132	0,034857358	0,843980537	-4,274770931
527	26	0	A_24_P511187	ENST00000569137	0,462433321	6,13115261	2,76172258	0,034860501	0,843980537	-4,274781229
446	38	0	A_23_P317082	NM_138780	0,762149832	6,010675434	2,761428171	0,034873619	0,843980537	-4,274824204
244	1	0	A_32_P226009	NM_023073	0,493657303	7,785694633	2,761359679	0,034876672	0,843980537	-4,274834203
235	84	0	A_23_P341860	ENST00000538936	-0,461710772	7,441087885	-2,761199326	0,03488382	0,843980537	-4,274857613
193	74	0	A_24_P390172	NM_024979	-0,532273261	6,633474365	-2,761154154	0,034885833	0,843980537	-4,274864207
80	43	0	A_24_P941946	ENST00000427175	-0,470016935	7,071997542	-2,760777728	0,03490262	0,843980537	-4,274919169
302	27	0	A_24_P406006	NM_024830	-0,376451328	8,374284854	-2,759996495	0,034937487	0,843980537	-4,275033263
210	74	0	A_32_P109777	THC2555723	-0,757654896	7,496646507	-2,759654135	0,034952779	0,843980537	-4,275083275
493	42	0	A_24_P928112	A_24_P928112	0,92491608	5,350042815	2,759467899	0,0349611	0,843980537	-4,275110483
170	33	0	A_23_P45248	NM_002351	0,452927401	5,31735491	2,759330006	0,034967263	0,843980537	-4,27513063
356	9	0	A_32_P185628	A_32_P185628	0,508092082	6,188540349	2,759151505	0,034975242	0,843980537	-4,275156712
168	11	0	A_23_P121596	NM_002704	0,76526935	6,176249812	2,758890838	0,034986898	0,843980537	-4,275194803
98	65	0	A_23_P24361	NM_000842	-0,634239722	6,204547771	-2,7588331	0,034989481	0,843980537	-4,275203241
336	7	0	A_32_P48842	NM_001163438	0,582096655	6,998790722	2,758529428	0,035003066	0,843980537	-4,275247623
254	17	0	A_24_P919340	A_24_P919340	0,535904955	6,372357767	2,758299671	0,035013348	0,843980537	-4,275281206
253	76	0	A_23_P323178	ENST00000302728	-0,757587832	6,347808824	-2,757710816	0,035039716	0,843980537	-4,275367292
97	68	0	A_32_P13565	THC2683527	-0,408056028	7,896269596	-2,75742126	0,03505269	0,843980537	-4,275409631
103	61	0	A_24_P929221	ENST00000436137	-0,477091187	5,441550993	-2,757376816	0,035054682	0,843980537	-4,27541613
181	5	0	A_32_P217261	ENST00000570137	0,495373843	7,351862793	2,757210069	0,035062156	0,843980537	-4,275440514
86	49	0	A_32_P3322	NR_102746	-0,575508625	5,982021719	-2,757082499	0,035067875	0,843980537	-4,275459171
85	6	0	A_24_P924230	A_24_P924230	0,50385505	5,471219622	2,756762345	0,035082232	0,843980537	-4,275505997
482	30	0	A_32_P125736	A_32_P125736	0,482629868	6,942571084	2,756688254	0,035085556	0,843980537	-4,275516834
269	19	0	A_24_P234768	NM_001040173	0,539639104	5,3980582	2,755963536	0,035118083	0,843980537	-4,275622858
275	35	0	A_24_P419309	NM_004814	-0,459594064	9,319752898	-2,755634789	0,035132849	0,843980537	-4,275670963
166	17	0	A_23_P45424	NM_012278	0,39087521	7,776117254	2,755593654	0,035134697	0,843980537	-4,275676983
474	84	0	A_24_P67308	NR_026660	0,363403736	14,67104911	2,754877853	0,035166872	0,843980537	-4,275781751
315	12	0	A_24_P74559	NM_013385	0,637917028	5,993752611	2,754557529	0,035181281	0,843980537	-4,275828645
455	52	0	A_32_P150786	THC2714964	0,579967125	5,690133719	2,754226659	0,035196171	0,843980537	-4,27587709
297	82	0	A_32_P21848	THC2716178	-0,519356679	7,279952964	-2,754224705	0,035196259	0,843980537	-4,275877377
368	33	0	A_32_P204137	NM_000809	0,485591752	5,198236123	2,75420376	0,035197202	0,843980537	-4,275880443
128	64	0	A_23_P29081	NM_002626	-0,376602183	8,657292674	-2,753991763	0,035206746	0,843980537	-4,275911487
133	21	0	A_23_P414328	NM_145811	0,406685606	5,773307173	2,753762486	0,035217072	0,843980537	-4,275945065
417	42	0	A_23_P40096	NM_000312	0,791438888	6,636408966	2,75277418	0,035261618	0,843980537	-4,276089839
14	74	0	A_24_P78862	NM_152346	-0,499194059	6,881274618	-2,752352585	0,035280639	0,843980537	-4,276151615
81	79	0	A_23_P131899	NM_080489	-0,482992426	6,639363549	-2,752142585	0,035290117	0,843980537	-4,276182391
456	84	0	A_23_P66766	NM_001160246	0,559715363	11,25609133	2,750690437	0,035355738	0,843980537	-4,276395279
455	72	0	A_24_P384588	NR_033351	-0,411893949	8,787280567	-2,750408773	0,035368481	0,843980537	-4,276436587
237	80	0	A_23_P135454	NM_006796	-0,401181744	8,453615229	-2,749826987	0,035394819	0,843980537	-4,276521925
97	80	0	A_24_P919789	AF130080	-0,969439818	6,825398907	-2,748365658	0,035461067	0,843980537	-4,276736371
251	8	0	A_23_P161156	NM_182755	0,474912967	8,837190148	2,747741015	0,035489425	0,843980537	-4,276828076
311	85	0	A_24_P226037	NM_002138	0,458325216	9,956922965	2,747483956	0,035501103	0,843980537	-4,276865822
239	62	0	A_24_P100387	NM_203391	-0,416026638	7,102974277	-2,746903896	0,035527468	0,843980537	-4,276951012
356	3	0	A_32_P149435	NM_173495	0,892431724	6,734552874	2,746901642	0,035527571	0,843980537	-4,276951344
348	6	0	A_24_P365322	NM_152601	0,570154548	7,72099241	2,74667067	0,035538075	0,843980537	-4,276985271
18	71	0	A_24_P177948	NM_173812	-0,637916466	5,715159465	-2,746587054	0,035541879	0,843980537	-4,276997554
187	3	0	A_24_P322908	NM_001145073	0,629779555	8,000295098	2,745984444	0,035569304	0,843980537	-4,277086091
113	63	0	A_23_P145930	THC2765070	-0,66526777	5,963459278	-2,745631182	0,035585392	0,843980537	-4,277138003
200	3	0	A_24_P752279	A_24_P752279	0,546607293	7,36855683	2,74535815	0,035597832	0,843980537	-4,277178131
259	19	0	A_24_P470809	A_24_P470809	0,444491159	6,432295337	2,745128994	0,035608276	0,843980537	-4,277211814
225	78	0	A_24_P221515	ENST00000506539	-0,806853345	5,80621646	-2,744993726	0,035614442	0,843980537	-4,277231698
335	22	0	A_23_P302005	NM_006873	0,572438663	8,457578632	2,744056925	0,035657181	0,843980537	-4,277369437
200	82	0	A_24_P161525	AY956764	0,572201065	10,3507656	2,743922492	0,035663319	0,843980537	-4,277389208
185	82	0	A_32_P181077	NM_203447	-0,619987137	5,271744436	-2,743390125	0,035687636	0,843980537	-4,277467511
131	65	0	A_24_P156922	NM_001007098	-0,537480956	6,239077597	-2,742816399	0,035713862	0,843980537	-4,277551918
65	49	0	A_32_P6917	NM_025207	-0,657601735	8,577770467	-2,742634154	0,035722197	0,843980537	-4,277578734
197	84	0	A_23_P422778	NM_004518	-0,649449247	6,637242321	-2,742559397	0,035725616	0,843980537	-4,277589735
412	35	0	A_32_P210872	NM_001037558	0,476757799	5,491470564	2,741879102	0,035756752	0,843980537	-4,277689857
87	29	0	A_23_P204052	NM_031989	-0,379392677	10,143731	-2,740742739	0,035808827	0,843980537	-4,277857166
125	74	0	A_23_P110288	NM_024751	-0,368655578	7,422691119	-2,740459525	0,035821818	0,843980537	-4,277898877
119	81	0	A_32_P166392	A_32_P166392	0,552299509	12,68351838	2,740331724	0,035827682	0,843980537	-4,277917701
428	47	0	A_32_P156237	BC010054	0,625686873	6,265907103	2,740323463	0,035828061	0,843980537	-4,277918917
490	5	0	A_23_P82990	NM_033014	0,389296136	5,432041154	2,740099423	0,035838343	0,843980537	-4,277951919
303	11	0	A_23_P148204	NM_017944	0,450913457	8,414109427	2,739715785	0,035855958	0,843980537	-4,278008436
189	9	0	A_23_P302386	ENST00000460054	0,524337645	6,680031277	2,739672176	0,035857961	0,843980537	-4,278014861
111	23	0	A_24_P521409	ENST00000472940	1,078490236	5,90335362	2,739578599	0,035862259	0,843980537	-4,278028648
509	16	0	A_23_P211773	NM_012287	0,406234168	9,475045075	2,738647633	0,035905051	0,843980537	-4,278165844
483	13	0	A_23_P80684	NM_001278221	-0,431530709	6,147795139	-2,738288095	0,035921592	0,843980537	-4,278218843
251	33	0	A_23_P65629	NM_021161	0,571971508	5,824118818	2,738276837	0,03592211	0,843980537	-4,278220503
249	27	0	A_23_P171385	NM_032335	0,550031332	7,959440492	2,737438698	0,035960703	0,843980537	-4,278344084
327	20	0	A_23_P314250	NM_033387	0,38436118	7,546784759	2,737269844	0,035968483	0,843980537	-4,278368986
31	60	0	A_23_P305507	NM_003286	-0,420173297	10,14979975	-2,737230592	0,035970292	0,843980537	-4,278374775
184	65	0	A_24_P331704	NM_182507	-0,751518694	7,296584303	-2,737130683	0,035974897	0,843980537	-4,278389511
166	80	0	A_24_P918106	THC2649445	-0,595353006	4,516904369	-2,737106579	0,035976008	0,843980537	-4,278393066
38	6	0	A_23_P368862	NM_178349	-0,519691688	4,870961491	-2,736433314	0,036007055	0,843980537	-4,278492383
525	36	0	A_24_P913609	A_24_P913609	0,753117728	5,2148057	2,736067903	0,036023918	0,843980537	-4,278546298
29	80	0	A_24_P664258	A_24_P664258	-0,686335192	5,833468064	-2,735769327	0,036037703	0,843980537	-4,278590358
71	62	0	A_32_P165557	ENST00000451962	-0,604049781	5,431446079	-2,735595268	0,036045742	0,843980537	-4,278616047
222	34	0	A_23_P345575	NM_001455	-0,459974043	10,31008883	-2,735589658	0,036046001	0,843980537	-4,278616875
149	45	0	A_23_P255695	NM_006632	-0,392071968	5,819775164	-2,735135276	0,036066996	0,843980537	-4,278683943
192	24	0	A_24_P48522	NM_018111	0,567944525	9,92990848	2,73423772	0,036108506	0,843980537	-4,278816462
432	23	0	A_32_P120043	NM_153711	0,704332703	5,927255671	2,734012341	0,036118937	0,843980537	-4,278849747
464	4	0	A_24_P578445	A_24_P578445	-0,560990967	6,64902598	-2,733391258	0,0361477	0,843980537	-4,278941484
212	78	0	A_23_P20328	CX165016	-0,400376034	9,053264962	-2,733169886	0,036157958	0,843980537	-4,278974188
240	37	0	A_23_P217411	NM_006306	-0,38301975	12,00728003	-2,733086357	0,03616183	0,843980537	-4,278986529
176	13	0	A_24_P878388	A_24_P878388	0,608034744	9,370909929	2,732264553	0,036199943	0,843980537	-4,279107968

505	49	0	A_24_P265064	NM_020717	0,426920149	6,316977611	2,731400514	0,036240061	0,843980537	-4,279235693
375	31	0	A_32_P148322	NM_004932	0,655360802	5,516172776	2,730997026	0,036258812	0,843980537	-4,279295354
327	15	0	A_32_P78385	NR_027768	0,514083911	6,219025148	2,730593093	0,036277594	0,843980537	-4,279355091
57	32	0	A_23_P94397	NM_005014	-0,403516262	6,153305611	-2,730415996	0,036285833	0,843980537	-4,279381284
232	66	0	A_23_P205296	NM_018199	-0,3868175	7,26460022	-2,729003435	0,036351613	0,843980537	-4,279590281
513	37	0	A_23_P321307	NM_021599	0,496497847	6,941396596	2,728977853	0,036352805	0,843980537	-4,279594067
340	82	0	A_24_P372613	NM_001164	-0,397034806	9,638032962	-2,728528855	0,036373742	0,843980537	-4,279660526
394	19	0	A_24_P943258	A_24_P943258	0,602204353	5,054162121	2,728136709	0,036392039	0,843980537	-4,27971858
344	15	0	A_32_P63284	BC036627	0,47526191	5,69617537	2,72719796	0,036435879	0,843980537	-4,279857593
338	39	0	A_24_P44780	NM_001145472	0,527254415	6,398110801	2,727023866	0,036444015	0,843980537	-4,27988338
22	72	0	A_24_P927287	BC029545	-0,830414029	4,844770055	-2,726754921	0,036456588	0,843980537	-4,279923219
128	69	0	A_23_P126159	NM_002143	-0,48804594	6,755966606	-2,72663559	0,036462169	0,843980537	-4,279940897
386	45	0	A_23_P158096	ENST00000468565	0,421856344	8,903836788	2,726186387	0,036483183	0,843980537	-4,280007452
390	66	0	A_23_P134085	NM_173515	0,367218519	8,781299043	2,726088145	0,036487781	0,843980537	-4,28002201
80	3	0	A_32_P215556	NR_039993	0,491341661	8,179795255	2,724805988	0,036547843	0,843980537	-4,280212054
62	58	0	A_32_P30477	THC2746700	-0,942808689	5,529184462	-2,724673165	0,036554071	0,843980537	-4,280231747
157	80	0	A_24_P478940	THC2668815	0,414468086	10,5644254	2,724637898	0,036555724	0,843980537	-4,280236976
348	17	0	A_24_P651859	A_24_P651859	0,40435789	5,640654955	2,723783542	0,036595815	0,843980537	-4,280363677
33	47	0	A_23_P346813	NM_002578	-0,613030336	6,670715054	-2,723722137	0,036598698	0,843980537	-4,280372785
203	75	0	A_24_P187174	A_24_P187174	-0,911258124	6,980018006	-2,723603731	0,036604258	0,843980537	-4,280390348
111	34	0	A_24_P279520	NM_015510	0,366780889	8,686252361	2,722498015	0,036656227	0,843980537	-4,280554406
25	63	0	A_32_P78349	AK022024	-0,610421852	5,856548169	-2,722314668	0,036664852	0,843980537	-4,280581617
201	10	0	A_24_P237036	NM_003807	0,673583365	5,795215511	2,721699981	0,036693785	0,843980537	-4,280672859
22	58	0	A_23_P104086	NM_014675	-0,549345152	7,004124501	-2,72140403	0,036707723	0,843980537	-4,280716798
458	44	0	A_24_P239718	A_24_P239718	0,620535167	5,858673421	2,721281993	0,036713473	0,843980537	-4,280734918
24	63	0	A_23_P9209	NM_018376	-0,456868175	7,426261095	-2,720743306	0,036738863	0,843980537	-4,280814912
151	81	0	A_23_P134139	NM_001446	-0,499004222	5,851004309	-2,720586687	0,036746248	0,843980537	-4,280838174
177	77	0	A_23_P70998	NR_034084	0,380903498	9,070854663	2,720225183	0,036763301	0,843980537	-4,280891871
461	36	0	A_23_P39790	NM_015717	0,466087828	5,517675809	2,720128373	0,03676787	0,843980537	-4,280906252
352	12	0	A_23_P45864	NM_003285	-0,543272835	6,970508849	-2,718854352	0,036828045	0,843980537	-4,281095565
234	72	0	A_23_P11787	NM_030761	-0,463406623	6,018392626	-2,718665324	0,036836982	0,843980537	-4,281123662
147	19	0	A_24_P931932	A_24_P931932	0,474731377	5,924337477	2,718107158	0,036863387	0,843980537	-4,281206641
310	72	0	A_24_P336276	NM_013272	-0,508371227	7,336963703	-2,717816377	0,03687715	0,843980537	-4,281249877
164	16	0	A_24_P574914	AI796784	0,43917801	6,341385467	2,717700779	0,036882623	0,843980537	-4,281267067
475	85	0	A_32_P186474	NM_013277	0,856931716	8,251062884	2,717401844	0,036896781	0,843980537	-4,281311524
21	75	0	A_32_P44047	A_32_P44047	-0,600512452	6,123766715	-2,716946399	0,036918362	0,843980537	-4,281379266
485	6	0	A_24_P280148	NM_080817	0,510806149	5,525291642	2,716707792	0,036929674	0,843980537	-4,281414762
288	80	0	A_24_P941922	ENST00000315544	-0,46618143	7,884011232	-2,716310718	0,036948506	0,843980537	-4,281473839
416	62	0	A_23_P77653	NM_000339	0,927644785	7,129303862	2,715806738	0,036972424	0,843980537	-4,281548836
529	64	0	A_32_P83326	NR_027439	-0,424828409	10,56346697	-2,714446022	0,037037085	0,843980537	-4,281751403
424	83	0	A_23_P111487	NM_001128853	-0,362391253	7,701435398	-2,713750953	0,037070161	0,843980537	-4,281854921
84	27	0	A_23_P501722	NM_139022	0,392784024	5,706423078	2,713467098	0,037083679	0,843980537	-4,281897205
316	74	0	A_24_P222844	NM_018061	0,471110994	8,637344917	2,712445821	0,037132356	0,843980537	-4,282049379
194	13	0	A_32_P129669	XM_003960833	0,612876129	6,745091997	2,712193874	0,037144375	0,843980537	-4,282086931
241	51	0	A_24_P76644	ENST00000568556	0,65317942	10,34638175	2,711239205	0,037189955	0,843980537	-4,282229254
220	30	0	A_24_P926529	BC029395	0,59829933	5,673489711	2,711089438	0,037197111	0,843980537	-4,282251587
187	30	0	A_24_P234116	NM_017860	0,384261783	10,4826386	2,710878384	0,037207198	0,843980537	-4,28228306
10	78	0	A_23_P128609	NM_005073	-0,556616858	5,72664991	-2,710370535	0,037231482	0,843980537	-4,282358806
340	40	0	A_23_P141946	NM_001042544	-0,368852007	10,20354966	-2,709908268	0,037253601	0,843980537	-4,282427767
333	8	0	A_32_P138178	BE835389	0,412791776	7,901956401	2,709868692	0,037255496	0,843980537	-4,282433672
398	19	0	A_32_P123088	NM_001164468	0,546369054	7,594024156	2,709480618	0,037274077	0,843980537	-4,282491576
529	32	0	A_24_P548624	ENST00000426704	0,363590308	6,390681069	2,708477138	0,03732217	0,843980537	-4,282641348
382	69	0	A_32_P122662	NM_018988	0,431263591	9,042833642	2,708467936	0,037322612	0,843980537	-4,282642722
312	30	0	A_23_P139881	NM_001759	0,552840814	6,139119814	2,708315607	0,037329918	0,843980537	-4,282665463
382	32	0	A_32_P110178	THC2766102	-0,379967724	6,546115497	-2,706450098	0,037419524	0,843980537	-4,282944083
115	57	0	A_24_P228026	NM_144611	-0,380870949	8,662748201	-2,706074139	0,03743761	0,843980537	-4,28300026
186	74	0	A_24_P601915	AL157490	-0,569117912	6,168419564	-2,705902041	0,037445893	0,843980537	-4,283025978
210	21	0	A_32_P764462	AK097855	0,549529137	5,940209111	2,70542623	0,037468802	0,843980537	-4,283097093
406	51	0	A_23_P150963	NM_001282616	0,50806973	6,025635599	2,705394055	0,037470352	0,843980537	-4,283101903
92	76	0	A_32_P138586	A_32_P138586	0,394076634	9,549863364	2,705162529	0,037481505	0,843980537	-4,283136512
222	20	0	A_24_P49371	NM_017867	0,40262266	8,754600089	2,704279289	0,037524087	0,843980537	-4,283268574
311	43	0	A_24_P96709	XM_005244781	-0,357562829	9,7718416	-2,704033386	0,037535951	0,843980537	-4,28330535
441	46	0	A_24_P25252	NM_015208	0,384519059	9,504145273	2,703857576	0,037544436	0,843980537	-4,283331646
501	30	0	A_32_P142586	A_32_P142586	0,886422557	5,47569835	2,703780218	0,03754817	0,843980537	-4,283343217
499	77	0	A_23_P348349	THC2703908	-0,515749413	6,363542836	-2,703432851	0,037564943	0,843980537	-4,283395179
23	75	0	A_24_P925786	A_24_P925786	-0,543429498	6,052405231	-2,70251782	0,037609164	0,843980537	-4,283532095
9	56	0	A_24_P127748	NM_015097	-0,428984946	6,52351668	-2,702083437	0,037630177	0,843980537	-4,28359711
470	20	0	A_32_P166235	A_32_P166235	-0,429781068	7,252018474	-2,701326887	0,037666803	0,843980537	-4,283710372
37	10	0	A_23_P138796	NM_130847	0,641049546	8,36442085	2,701100409	0,037677775	0,843980537	-4,283744285
401	15	0	A_24_P161197	NR_003680	0,419275504	5,862288449	2,700141025	0,037724292	0,843980537	-4,283887979
197	68	0	A_32_P437004	NM_175569	-0,877252334	5,666084295	-2,699674232	0,037746947	0,843980537	-4,283957914
486	23	0	A_24_P247902	NM_014510	-0,384316876	7,771740394	-2,699599583	0,037750571	0,843980537	-4,2839691
146	61	0	A_23_P83289	NM_016174	-0,448450872	6,451860424	-2,699104324	0,037774627	0,843980537	-4,284043318
320	75	0	A_24_P297551	NM_178539	0,540098166	7,143473862	2,698942582	0,037782486	0,843980537	-4,284067559
66	23	0	A_24_P260371	NM_015393	0,503258833	5,57408346	2,698634148	0,037797479	0,843980537	-4,284113791
148	64	0	A_32_P23512	THC2629801	-0,411603738	5,875649368	-2,698359328	0,037810843	0,843980537	-4,284154989
493	33	0	A_24_P930432	BX648337	0,52652063	6,12673752	2,697953014	0,037830611	0,843980537	-4,284215909
493	79	0	A_32_P107029	NM_004851	-1,02430849	7,06170965	-2,697537056	0,03785086	0,843980537	-4,284278285
42	76	0	A_32_P366909	NR_103561	-0,787076809	6,323589748	-2,696383805	0,037907061	0,843980537	-4,28445128
159	53	0	A_23_P79331	NM_022152	-0,466240917	6,605158192	-2,696199996	0,037916027	0,843980537	-4,28447886
112	59	0	A_23_P88466	NM_018958	-0,458481228	6,729831258	-2,695637591	0,037943474	0,843980537	-4,284563262
25	72	0	A_24_P941526	NM_001009185	-0,670958058	6,075928954	-2,695085593	0,037970435	0,843980537	-4,284646121
427	38	0	A_24_P332595	A_24_P332595	0,902262396	5,627710248	2,694954494	0,037976841	0,843980537	-4,284665802
301	14	0	A_23_P129835	NM_032192	0,64772063	6,195818894	2,693753273	0,038035592	0,843980537	-4,28484619
278	16	0	A_24_P192434	NM_017489	0,498599275	7,212784639	2,693662355	0,038040042	0,843980537	-4,284859847
494	4	0	A_32_P116088	A_32_P116088	-0,630005233	6,185627226	-2,693618114	0,038042208	0,843980537	-4,284866493
127	73	0	A_23_P116376	NM_024811	-0,501303594	9,40453513	-2,693395135	0,038053127	0,843980537	-4,284899989
205	67	0	A_24_P902195	A_24_P902195	-1,025155785	6,972303561	-2,693351932	0,038055243	0,843980537	-4,28490648
131	73	0	A_24_P919044	A_24_P919044	-0,760760482	5,459819859	-2,693093314	0,038067912	0,843980537	-4,284945335
384	78	0	A_23_P44581	NM_014287	-0,414299715	10,43534169	-2,692476355	0,038098153	0,843980537	-4,285038045
2	73	0	A_24_P910381	A_24_P910381	-0,928292519	6,024367298	-2,691952373	0,038123857	0,843980537	-4,285116802
171	52	0	A_23_P83028	NM_021111	0,418073253	8,394686847	2,690952381	0,038172963	0,843980537	-4,285267153
112	69	0	A_24_P945293	NM_016079	-0,577275844	6,658528948	-2,690861082	0,03817745	0,843980537	-4,285280883
223	76	0	A_23_P255166	NM_018460	-0,37646149	6,827776698	-2,690822829	0,03817933	0,843980537	-4,285286636

92	81	0	A_24_P934345	AY358728	-0,676249679	6,77421235	-2,690470592	0,038196647	0,843980537	-4,285339614
9	66	0	A_24_P318073	NM_032795	-0,544349612	8,509569615	-2,690266883	0,038206665	0,843980537	-4,285370256
231	55	0	A_23_P256033	NM_001958	-0,416306086	12,07785609	-2,689599243	0,03823952	0,843980537	-4,285470701
166	70	0	A_24_P928408	ENST00000483346	-0,393367538	6,523264351	-2,689381598	0,038250237	0,843980537	-4,285503451
62	51	0	A_32_P209148	NR_027035	-0,503779496	6,212165411	-2,689059268	0,038266115	0,843980537	-4,28555196
37	56	0	A_23_P433690	NM_018555	-0,359558714	8,794350217	-2,688340224	0,038301561	0,843980537	-4,285660194
322	18	0	A_23_P20303	NM_001385	0,444947258	5,502180613	2,688205589	0,038308201	0,843980537	-4,285680464
271	34	0	A_32_P197561	NM_024007	0,497793369	7,769049754	2,688155672	0,038310664	0,843980537	-4,285687979
408	83	0	A_24_P17052	AF116677	-0,452839743	5,98397839	-2,687906478	0,038322959	0,843980537	-4,2857255
14	55	0	A_23_P434548	XM_003846346	-0,617347627	5,332930366	-2,687234871	0,038356118	0,843980537	-4,285826642
105	80	0	A_24_P250227	NM_021724	-0,765292409	7,024324075	-2,687121004	0,038361743	0,843980537	-4,285843792
203	73	0	A_32_P421898	AF147302	-0,635807647	5,797423487	-2,686634243	0,038385799	0,843980537	-4,285917118
449	22	0	A_23_P9711	NM_006040	0,463630144	7,438880792	2,685091283	0,038462159	0,843980537	-4,286149648
147	11	0	A_24_P101561	A_24_P101561	0,468896232	7,910252423	2,684725546	0,038480284	0,843980537	-4,286204788
195	63	0	A_32_P75544	ENST00000522989	0,407839108	6,723269678	2,684694958	0,0384818	0,843980537	-4,2862094
240	23	0	A_24_P260134	NM_178177	0,745797546	5,296206029	2,684454329	0,03849373	0,843980537	-4,286245683
527	31	0	A_23_P370625	NM_020451	-0,464090635	10,19706548	-2,684418945	0,038495484	0,843980537	-4,286251019
203	19	0	A_23_P7882	NM_015482	0,673985683	6,327408809	2,68396444	0,03851803	0,843980537	-4,286319563
38	7	0	A_24_P306704	A_24_P306704	-0,550046007	6,503519025	-2,683746855	0,038528828	0,843980537	-4,286352381
302	39	0	A_24_P249626	NM_017525	0,598209691	6,387502281	2,683109278	0,038560489	0,843980537	-4,286448564
146	60	0	A_24_P542673	ENST00000417476	-0,436091255	5,80848089	-2,683064001	0,038562738	0,843980537	-4,286455395
467	70	0	A_24_P366122	NM_024722	-0,737829609	6,977070761	-2,681764293	0,038627369	0,843980537	-4,286651548
308	1	0	A_23_P214222	NM_002356	0,375298488	13,00830599	2,681529922	0,038639036	0,843980537	-4,286686931
38	72	0	A_24_P929695	AK098414	-0,750491175	5,383574171	-2,68134321	0,038648334	0,843980537	-4,286715121
414	37	0	A_23_P399604	A_23_P399604	0,481675467	6,441958286	2,681256789	0,038652638	0,843980537	-4,28672817
222	17	0	A_32_P25514	NM_198904	0,505963476	6,119630278	2,681183237	0,038656301	0,843980537	-4,286739276
121	75	0	A_24_P299489	ENST00000488635	-0,618444818	5,928887208	-2,680612061	0,038684764	0,843980537	-4,286825533
211	20	0	A_23_P218047	NM_000424	0,604817541	5,467522954	2,679824956	0,038724025	0,843980537	-4,286944432
197	34	0	A_24_P490877	ENST00000523507	0,448686863	4,97953545	2,679088134	0,038760816	0,843980537	-4,287055771
464	54	0	A_23_P58497	NM_032175	0,628363224	6,227833645	2,679062785	0,038762082	0,843980537	-4,287059602
135	8	0	A_32_P755377	BC034617	0,401912015	6,1903296	2,678922215	0,038769106	0,843980537	-4,287080847
4	75	0	A_23_P201538	NM_002228	0,42116499	12,56794138	2,678741054	0,038778159	0,843980537	-4,287108229
356	48	0	A_24_P926006	A_24_P926006	0,644556606	6,085280746	2,678581078	0,038786156	0,843980537	-4,28713241
288	59	0	A_24_P179407	NM_018351	0,473368349	7,534259948	2,67837848	0,038796286	0,843980537	-4,287163037
9	17	0	A_32_P38989	NR_002712	0,473435244	5,520370274	2,677160107	0,038857265	0,843980537	-4,28734727
498	12	0	A_23_P81233	NM_001029875	-0,543343289	6,288694264	-2,676191317	0,038905826	0,843980537	-4,287493831
523	37	0	A_32_P215100	NR_027257	0,365290823	5,9229557	2,676143436	0,038908227	0,843980537	-4,287501076
212	14	0	A_23_P332399	NM_016315	0,473085936	10,32765194	2,675908488	0,038920015	0,843980537	-4,287536629
387	68	0	A_24_P568645	A_24_P568645	-0,619479601	7,203670221	-2,675700846	0,038930436	0,843980537	-4,287568053
126	74	0	A_23_P132730	NM_004803	-0,531295105	6,379444198	-2,674482809	0,038991625	0,843980537	-4,287752441
270	62	0	A_24_P85574	NM_000506	-0,698533704	6,596258052	-2,674133272	0,039009203	0,843980537	-4,287805371
126	39	0	A_23_P123336	NM_018444	0,489146849	9,118394234	2,674033567	0,039014219	0,843980537	-4,287820471
160	29	0	A_24_P926484	A_24_P926484	0,579133811	7,796264175	2,67282943	0,03907485	0,843980537	-4,288002881
68	68	0	A_23_P405216	NM_001145083	-0,507278476	7,352214771	-2,672700278	0,039081359	0,843980537	-4,288022451
45	68	0	A_23_P214080	NM_001964	0,381316047	11,83642357	2,672476291	0,03909265	0,843980537	-4,288056394
459	22	0	A_23_P210763	NM_000214	0,397326319	6,221977491	2,672132542	0,039109986	0,843980537	-4,288108492
35	55	0	A_32_P542318	NM_001278199	-0,920165779	5,800696543	-2,671421001	0,039145896	0,843980537	-4,288216354
447	32	0	A_24_P213487	AF271775	0,60247258	7,74292859	2,671145868	0,039159791	0,843980537	-4,28825807
299	18	0	A_24_P306527	A_24_P306527	0,459688039	7,372414872	2,670971482	0,039168601	0,843980537	-4,288284514
192	71	0	A_24_P940441	BC034487	-0,422814944	5,927893063	-2,670246153	0,039205266	0,843980537	-4,28839452
164	18	0	A_24_P282251	NM_021871	0,881620261	6,215056713	2,670236422	0,039205758	0,843980537	-4,288395996
89	49	0	A_23_P112026	NM_002164	-0,462492711	5,895802718	-2,669867566	0,039224418	0,843980537	-4,288451951
184	53	0	A_24_P525917	NM_012340	-0,478112206	6,511185862	-2,66983241	0,039226197	0,843980537	-4,288457284
345	31	0	A_23_P125147	NM_004249	-0,439579545	7,445535455	-2,668821998	0,039277364	0,843980537	-4,288610609
132	75	0	A_23_P204791	NM_000620	-0,489393805	6,257839871	-2,668001	0,039318993	0,843980537	-4,288735238
348	85	0	A_23_P252052	NM_182909	-0,394944521	7,513779084	-2,667822913	0,039328029	0,843980537	-4,288762278
250	19	0	A_24_P934063	A_24_P934063	0,757968299	6,137361188	2,667656296	0,039336485	0,843980537	-4,288787578
145	10	0	A_24_P929533	A_24_P929533	0,417857804	6,007127787	2,667509357	0,039343944	0,843980537	-4,288809891
127	67	0	A_24_P148836	NM_173546	-0,375474631	8,618752237	-2,667368677	0,039351087	0,843980537	-4,288831255
505	41	0	A_23_P25396	NM_005123	0,647410247	6,337822252	2,666798852	0,039380033	0,843980537	-4,288917803
227	40	0	A_24_P374427	NM_178566	-0,425035524	6,116833538	-2,666572553	0,039391535	0,843980537	-4,288952181
86	68	0	A_23_P153155	NM_001480	-1,379642637	6,036416886	-2,6665695	0,03939169	0,843980537	-4,288952645
259	53	0	A_24_P787947	NM_001005404	0,59585714	7,565933282	2,666500077	0,039395219	0,843980537	-4,288963191
114	67	0	A_32_P11947	A_32_P11947	-0,643310871	6,35099694	-2,665879777	0,039426769	0,843980537	-4,289057441
220	3	0	A_32_P157295	NM_001286117	0,470834703	7,722723157	2,665469722	0,03944764	0,843980537	-4,289119759
514	51	0	A_24_P85099	NM_003483	0,350022861	6,058941742	2,66529353	0,039456611	0,843980537	-4,289146539
481	85	0	A_24_P42514	NM_030939	0,350659439	11,58589222	2,665284286	0,039457082	0,843980537	-4,289147944
422	14	0	A_23_P136013	A_23_P136013	0,560586089	7,748290631	2,665225125	0,039460095	0,843980537	-4,289156937
107	27	0	A_23_P72077	NM_001559	0,634033015	6,858692168	2,664599555	0,039491969	0,843980537	-4,289252037
529	30	0	A_23_P122896	NM_003227	-0,422872285	7,584366228	-2,663416962	0,0395523	0,843980537	-4,289431886
140	3	0	A_23_P208085	NM_001025101	0,611564767	6,48050596	2,663048034	0,039571141	0,843980537	-4,28948801
174	81	0	A_24_P92106	ENST00000557899	-0,757569554	6,208025756	-2,662853487	0,039581081	0,843980537	-4,28951761
488	84	0	A_32_P71768	CU688181	-0,501234814	7,228201017	-2,662291224	0,039609823	0,843980537	-4,28960317
433	70	0	A_23_P28730	NM_020713	-0,370440828	11,08955751	-2,662110469	0,039619067	0,843980537	-4,289630679
175	79	0	A_24_P74508	NM_031481	-1,090434177	6,034389814	-2,662077839	0,039620736	0,843980537	-4,289635646
455	58	0	A_24_P134942	NM_000551	0,369512934	8,124965266	2,661926395	0,039628484	0,843980537	-4,289658696
204	70	0	A_32_P110699	THC2652013	-0,664315059	6,603512141	-2,661238357	0,039663703	0,843980537	-4,289763438
99	77	0	A_23_P432110	NM_014989	-0,860283901	5,899572808	-2,65966424	0,039744407	0,843980537	-4,290003182
249	8	0	A_24_P572303	A_24_P572303	0,504407368	6,390938397	2,658613163	0,039798393	0,843980537	-4,290163353
291	56	0	A_23_P56288	NM_052925	-0,410657667	8,344648694	-2,658504116	0,039803998	0,843980537	-4,290179974
461	10	0	A_23_P254025	NM_003408	-0,581749224	6,712555951	-2,658056934	0,039826994	0,843980537	-4,290248143
157	7	0	A_24_P22800	NM_002785	0,361145442	6,141438477	2,658046352	0,039827538	0,843980537	-4,290249756
495	56	0	A_32_P117313	NM_001171796	0,464384995	8,978484476	2,657621299	0,03984941	0,843980537	-4,290314563
305	11	0	A_24_P790361	A_24_P790361	0,606339988	6,315786817	2,657613979	0,039849786	0,843980537	-4,29031568
498	75	0	A_24_P93111	A_24_P93111	-0,549681712	6,56711756	-2,65753621	0,03985379	0,843980537	-4,290327538
304	17	0	A_23_P402670	NM_001763	0,655763052	5,397571455	2,656804721	0,039891464	0,843980537	-4,290439098
8	71	0	A_23_P333228	NM_020814	-0,67235016	5,364003745	-2,655880314	0,039939129	0,843980537	-4,290580129
472	81	0	A_23_P342641	NM_152697	-0,974888488	7,066369807	-2,655212191	0,039973617	0,843980537	-4,290682093
124	71	0	A_24_P576586	AL137705	-0,419450342	5,942833074	-2,654476858	0,040011612	0,843980537	-4,290794347
43	66	0	A_23_P111395	NM_003058	-0,815235366	5,642502279	-2,654281526	0,040021711	0,843980537	-4,290824172
17	76	0	A_24_P938352	NM_001874	-0,499807493	5,754272241	-2,654128237	0,040029639	0,843980537	-4,290847579
142	55	0	A_23_P408930	NR_027688	-0,564554549	5,451153446	-2,653589525	0,040057512	0,843980537	-4,290929851
4	40	0	A_23_P348138	NM_020831	-0,381134787	7,444286344	-2,65333784	0,040070542	0,843980537	-4,290968294
208	79	0	A_23_P113656	NM_006087	-0,548237652	6,82865476	-2,650422358	0,040221808	0,843980537	-4,29141391

140	10	0	A_24_P160482	ENST00000402026	1,086774914	5,38929134	2,650085464	0,040239327	0,843980537	-4,291465437
189	17	0	A_23_P373119	NR_002165	0,463609784	8,846583271	2,649895928	0,040249187	0,843980537	-4,29149443
252	84	0	A_24_P914638	NM_001173523	-1,070060118	6,238353243	-2,649883361	0,040249841	0,843980537	-4,291496352
274	16	0	A_24_P611182	AK055821	-0,350918335	8,224304926	-2,649692037	0,040259796	0,843980537	-4,29152562
253	70	0	A_24_P807935	THC2503637	-0,504929661	6,369796769	-2,648860003	0,040303122	0,843980537	-4,291652929
225	27	0	A_23_P4494	NM_024422	0,719962004	5,658361712	2,648827554	0,040304813	0,843980537	-4,291657895
333	21	0	A_23_P17330	NM_017859	-0,374960706	8,422830874	-2,646679071	0,040416926	0,843980537	-4,291986839
174	13	0	A_23_P23698	AK023372	0,762424802	5,575817572	2,646474983	0,040427593	0,843980537	-4,292018101
67	23	0	A_24_P18621	NM_153207	0,466053677	9,102583645	2,646443568	0,040429235	0,843980537	-4,292022913
361	9	0	A_24_P284213	NM_177974	0,366089628	9,241360064	2,64641134	0,04043092	0,843980537	-4,29202785
427	36	0	A_24_P250176	NM_170607	0,600830505	8,859671203	2,646403108	0,040431351	0,843980537	-4,292029111
193	21	0	A_23_P344578	NM_153707	0,454949477	6,858939326	2,646251255	0,04043929	0,843980537	-4,292052374
301	52	0	A_23_P12168	NM_002810	-0,354355662	12,26985644	-2,6459609	0,040454477	0,843980537	-4,292096859
210	69	0	A_32_P171921	BX339010	-0,442187551	9,468784728	-2,645895494	0,040457898	0,843980537	-4,292106881
360	39	0	A_24_P210829	NM_005009	-0,401461951	12,72997503	-2,6458888	0,040458249	0,843980537	-4,292107906
321	58	0	A_24_P375911	NR_037183	0,786901661	6,338756518	2,644874815	0,040511336	0,843980537	-4,292263305
364	15	0	A_23_P58137	ENST00000360096	0,629421548	6,39006267	2,64454698	0,040528516	0,843980537	-4,292313561
118	38	0	A_24_P235338	NM_007332	0,571953874	6,106031321	2,644540139	0,040528874	0,843980537	-4,29231461
251	13	0	A_23_P33723	NM_004244	0,443522698	5,53041385	2,644415174	0,040535425	0,843980537	-4,292333768
21	13	0	A_24_P383834	A_24_P383834	0,363712107	8,485257123	2,644364016	0,040538107	0,843980537	-4,292341612
277	12	0	A_32_P77102	NR_038461	0,45337976	6,073824872	2,644274741	0,040542788	0,843980537	-4,2923553
63	18	0	A_23_P25653	NM_012141	0,373129195	8,906221977	2,6439241	0,040561178	0,843980537	-4,292409066
251	3	0	A_24_P67585	NM_016206	0,359194051	7,414579139	2,643666405	0,0405747	0,843980537	-4,292448584
146	77	0	A_32_P122128	ENST00000514771	-0,684043491	5,897837561	-2,643282291	0,040594864	0,843980537	-4,292507498
170	84	0	A_32_P34826	NR_026543	-0,661945181	6,268819148	-2,642558294	0,040632898	0,843980537	-4,292618567
159	15	0	A_23_P5974	NM_080603	0,660288389	8,378583044	2,641619688	0,040682265	0,843980537	-4,292762608
14	44	0	A_24_P930842	A_24_P930842	-0,356367987	5,017530653	-2,641566317	0,040685074	0,843980537	-4,2927708
209	69	0	A_24_P78540	NM_003297	0,405224771	8,438571639	2,64017481	0,040758385	0,843980537	-4,292984452
471	49	0	A_24_P929424	AL521247	0,411712888	6,735047799	2,639636568	0,04078678	0,843980537	-4,293067126
18	23	0	A_32_P175098	A_32_P175098	-0,447514534	7,520943742	-2,639372273	0,040800731	0,843980537	-4,293107729
245	71	0	A_24_P156490	NM_002247	0,398741352	7,085601692	2,639162996	0,040811781	0,843980537	-4,293139883
114	62	0	A_23_P216742	NM_002581	-0,614441864	6,653553344	-2,638871843	0,04082716	0,843980537	-4,29318462
23	51	0	A_32_P77328	THC2661428	-0,548473779	5,302146649	-2,63840144	0,04085202	0,843980537	-4,293256913
408	52	0	A_32_P203404	NM_001044369	0,619830773	5,542588262	2,637880004	0,040879597	0,843980537	-4,293337064
437	82	0	A_23_P90743	NM_002909	-0,969291451	5,961401236	-2,637467896	0,040901405	0,843980537	-4,293400422
391	48	0	A_24_P334456	NM_197941	0,599693445	6,140948346	2,636558158	0,040949593	0,843980537	-4,293540325
124	55	0	A_32_P150152	TCONS_00024657	-0,610407969	5,91811008	-2,636436337	0,04095605	0,843980537	-4,293559063
244	18	0	A_24_P349807	NM_001145402	0,611200459	6,061932684	2,636204453	0,040968344	0,843980537	-4,293594733
1	73	0	A_32_P149011	CU680317	-0,482729073	7,057026674	-2,635922019	0,040983324	0,843980537	-4,293638184
441	33	0	A_24_P845223	M27126	0,487984223	5,915373435	2,635455219	0,041008095	0,843980537	-4,293710009
126	19	0	A_32_P46510	NM_198581	0,393566552	10,44707223	2,635068671	0,04102862	0,843980537	-4,293769497
130	79	0	A_24_P943370	NM_001040663	-0,873877874	6,145208315	-2,634660156	0,041050323	0,843980537	-4,293832375
31	33	0	A_24_P450092	AK024653	-0,359998259	7,286475259	-2,634257763	0,041071713	0,843980537	-4,293894322
209	13	0	A_23_P65174	NM_001040443	0,558861352	5,78275474	2,634176776	0,04107602	0,843980537	-4,293906791
474	33	0	A_23_P74701	NM_152890	0,365925343	6,341980405	2,634010746	0,04108485	0,843980537	-4,293932354
266	61	0	A_24_P390793	NM_001416	-0,44482877	13,20508791	-2,633968853	0,041087078	0,843980537	-4,293938804
104	57	0	A_24_P221407	NM_024854	-0,464063095	6,435477001	-2,633773717	0,04109746	0,843980537	-4,293968852
162	23	0	A_24_P183963	AK091034	0,524166786	5,255645628	2,631916011	0,041196432	0,843980537	-4,294255026
137	51	0	A_32_P58668	THC2505214	-0,414339719	5,794828522	-2,63172184	0,041206791	0,843980537	-4,294284951
140	84	0	A_24_P671490	AL080208	-0,99671671	6,312523369	-2,631353233	0,041226465	0,843980537	-4,294341764
94	84	0	A_23_P128808	NM_013345	-1,402945018	6,14841144	-2,631050354	0,041242639	0,843980537	-4,294388453
486	33	0	A_24_P364296	NM_001980	0,453994836	8,321746308	2,630340681	0,041280561	0,843980537	-4,294497873
336	69	0	A_24_P548866	NM_001099668	-0,454443017	5,976960286	-2,629903176	0,041303958	0,843980537	-4,294565345
158	7	0	A_24_P260325	NM_004744	0,507218549	6,079259578	2,629550315	0,041322839	0,843980537	-4,294619771
479	47	0	A_24_P920247	A_24_P920247	0,668457166	5,732874409	2,629453694	0,041328011	0,843980537	-4,294634676
179	31	0	A_23_P166269	NM_058186	0,482693415	5,839409119	2,629404984	0,041330618	0,843980537	-4,29464219
267	62	0	A_23_P149200	NM_001255	-0,376411229	10,45084092	-2,628621671	0,041372574	0,843980537	-4,294763047
186	60	0	A_23_P301328	ENST00000476313	-0,54342528	6,196200639	-2,628267662	0,04139155	0,843980537	-4,29481768
220	39	0	A_24_P528775	AI950319	0,495671763	5,838387638	2,627836842	0,041414656	0,843980537	-4,294884177
294	77	0	A_23_P88489	NM_172095	0,368570923	8,35028498	2,627200465	0,041448813	0,843980537	-4,294982424
157	10	0	A_24_P230466	A_24_P230466	0,693886251	7,118271101	2,626991374	0,041460042	0,843980537	-4,29501471
148	29	0	A_24_P230819	AB062477	0,71368919	5,08410914	2,62637982	0,041492904	0,843980537	-4,295109156
409	85	0	A_23_P205057	NM_001040429	0,591361545	9,039588059	2,625974934	0,041514676	0,843980537	-4,295171699
195	20	0	A_23_P111919	NM_013357	0,436877217	8,56108481	2,625496523	0,041540418	0,843980537	-4,295245612
475	29	0	A_32_P795513	NM_198271	0,504271969	5,980772029	2,625109136	0,041561274	0,843980537	-4,295305472
314	30	0	A_32_P109933	BX092869	0,712126583	5,394940507	2,625010392	0,041566592	0,843980537	-4,295320732
319	34	0	A_24_P172304	NM_139027	-0,38038365	7,09818404	-2,624703643	0,041583118	0,843980537	-4,295368141
148	78	0	A_24_P933378	A_24_P933378	-1,238697204	6,148111065	-2,624602577	0,041588564	0,843980537	-4,295383762
254	73	0	A_23_P164341	NM_014232	-0,346942059	9,521568784	-2,62355839	0,041644877	0,843980537	-4,295545195
169	70	0	A_24_P411749	NM_020455	-1,268696855	6,774386407	-2,623409233	0,041652928	0,843980537	-4,295568261
301	16	0	A_23_P108970	ENST00000316534	0,658657952	5,660242521	2,622888853	0,041681029	0,843980537	-4,295648743
167	19	0	A_23_P418083	NM_181714	0,455846369	8,043507565	2,622787217	0,04168652	0,843980537	-4,295664464
287	7	0	A_23_P42811	NM_176813	0,406543247	6,600342657	2,622569107	0,041698306	0,843980537	-4,295698204
250	31	0	A_24_P898915	NR_103825	-0,352223149	12,78878257	-2,622109546	0,04172315	0,843980537	-4,295769304
35	39	0	A_23_P83368	NM_016373	0,367231529	9,759670411	2,621793649	0,041740238	0,843980537	-4,295818184
505	40	0	A_23_P94434	NM_001039792	0,650245441	6,090202251	2,620826703	0,041792587	0,843980537	-4,295967845
28	33	0	A_23_P348063	NM_004711	-0,362959444	8,669123768	-2,620732675	0,041797682	0,843980537	-4,295982402
195	67	0	A_24_P797366	THC2667911	-0,610365917	6,238741171	-2,620327726	0,041819629	0,843980537	-4,296045099
229	46	0	A_24_P932339	NR_040065	0,651833626	5,931388217	2,620126648	0,041830532	0,843980537	-4,296076235
88	65	0	A_23_P31177	NM_018295	-0,575983945	6,787302619	-2,619942536	0,041840517	0,843980537	-4,296104746
227	19	0	A_32_P21459	THC2664391	0,547438628	7,959866791	2,619866114	0,041844663	0,843980537	-4,296116581
17	75	0	A_32_P64263	A_32_P64263	-0,950000027	6,96879668	-2,619670897	0,041855254	0,843980537	-4,296146815
273	42	0	A_23_P35830	NM_033036	0,597051573	6,909239619	2,619253559	0,041877907	0,843980537	-4,296211458
122	72	0	A_23_P28186	NM_000348	-0,574952659	6,346640237	-2,618733103	0,041906175	0,843980537	-4,296292089
512	48	0	A_23_P368870	NR_038868	0,564734235	5,920084044	2,618333248	0,041927907	0,843980537	-4,296354048
76	72	0	A_32_P9191	THC2707266	-0,986412829	5,517924597	-2,618311499	0,041929089	0,843980537	-4,296357418
125	78	0	A_24_P915784	NM_002410	-0,415144354	6,304688877	-2,618279016	0,041930855	0,843980537	-4,296362452
78	3	0	A_32_P234604	NM_005022	-0,452320727	12,63638386	-2,61817791	0,041936352	0,843980537	-4,296378121
271	64	0	A_23_P209032	NM_018443	-0,422435858	9,391952149	-2,617200544	0,041989534	0,843980537	-4,296529619
143	65	0	A_32_P212446	AL050068	-0,684349839	6,920380898	-2,616768888	0,042013045	0,843980537	-4,296596547
190	17	0	A_23_P68691	ENST00000334165	0,543605647	5,65066896	2,616511937	0,042027046	0,843980537	-4,296636393
97	81	0	A_23_P79272	NM_001003683	-0,786628423	5,852688552	-2,616363133	0,042035157	0,843980537	-4,296659471
471	63	0	A_32_P475642	ENST00000421806	0,351000693	9,651790587	2,616226855	0,042042587	0,843980537	-4,296680607
253	11	0	A_23_P161458	NM_001039702	0,411069917	6,479156071	2,616033061	0,042053155	0,843980537	-4,296710665

355	53	0	A_24_P586712	NM_198485	0,414519449	6,149366129	2,615582518	0,042077735	0,843980537	-4,296780556
13	54	0	A_23_P24870	NM_000610	0,498992014	8,072689083	2,615410294	0,042087135	0,843980537	-4,296807276
429	34	0	A_23_P17456	NM_006065	0,735460246	5,844762403	2,614825077	0,042119092	0,843980537	-4,296898083
483	7	0	A_32_P32250	NM_022063	0,391130983	10,01743424	2,614731194	0,042124221	0,843980537	-4,296912653
252	22	0	A_24_P163877	AF090890	0,49197386	5,097502018	2,61449566	0,042137093	0,843980537	-4,296949208
196	83	0	A_24_P85258	NM_001080484	-0,936065489	6,690067627	-2,613980042	0,042165284	0,843980537	-4,297029245
5	68	0	A_23_P53397	NM_138473	-0,797160964	7,596030776	-2,613738295	0,042178509	0,843980537	-4,297066776
270	11	0	A_23_P357284	NM_005282	0,370407012	6,890152486	2,613331463	0,042200774	0,843980537	-4,297129944
229	30	0	A_32_P36582	AK123649	0,610529413	8,325133138	2,61325456	0,042204984	0,843980537	-4,297141886
11	23	0	A_32_P133767	NM_198521	0,515537446	6,191218324	2,613059757	0,042215651	0,843980537	-4,297172138
225	80	0	A_23_P357229	NM_138612	-0,755328502	6,626645718	-2,612549822	0,042243588	0,843980537	-4,297251339
300	43	0	A_32_P37943	A_32_P37943	0,458263129	6,752113736	2,612187727	0,042263437	0,843980537	-4,297307588
41	17	0	A_23_P340922	NM_032370	-0,508424686	7,298727722	-2,612159229	0,042265	0,843980537	-4,297312015
226	11	0	A_23_P4133	NM_001158	0,514247008	6,625658463	2,611346771	0,042309575	0,843980537	-4,297438257
237	73	0	A_23_P429959	NM_020956	0,363823639	7,53018182	2,61130459	0,042311891	0,843980537	-4,297444812
57	62	0	A_24_P72139	NM_004536	-0,451105869	6,652630856	-2,611257389	0,042314482	0,843980537	-4,297452147
527	39	0	A_23_P170857	NM_002182	0,440873479	5,862631613	2,611163336	0,042319646	0,843980537	-4,297466765
255	5	0	A_24_P367191	TCONS_l2_00030167	0,490398268	9,859773726	2,611006452	0,042328262	0,843980537	-4,297491149
471	27	0	A_23_P897	NM_023938	0,567917222	5,641160571	2,610358285	0,042363876	0,843980537	-4,297591906
77	83	0	A_32_P168442	NR_047518	-1,262166555	6,408506871	-2,610326263	0,042365636	0,843980537	-4,297596885
351	83	0	A_24_P560536	AK025182	-0,78906652	6,387576578	-2,61000056	0,042383546	0,843980537	-4,297647526
153	15	0	A_23_P128384	NM_016226	0,533980857	7,56214407	2,609809709	0,042394044	0,843980537	-4,297677203
150	84	0	A_23_P208302	NM_000483	-0,639214706	5,542944114	-2,609589324	0,04240617	0,843980537	-4,297711476
47	53	0	A_32_P123804	NR_028348	-0,410494734	5,73919843	-2,608964479	0,04244057	0,843980537	-4,297808663
181	67	0	A_32_P9485	NM_000608	-0,936611261	6,460796069	-2,608414267	0,042470887	0,843980537	-4,297894264
473	73	0	A_24_P337143	ENST00000474523	-0,74852577	6,455490678	-2,608055177	0,042490685	0,843980537	-4,29795014
123	52	0	A_23_P413224	NM_004828	-0,400844201	6,203247979	-2,607807405	0,042504351	0,843980537	-4,297988699
464	9	0	A_23_P7172	NM_018290	0,418905246	9,484596369	2,607517911	0,042520325	0,843980537	-4,298033756
438	81	0	A_23_P322013	NR_040044	-0,768557681	6,016109521	-2,607505394	0,042521016	0,843980537	-4,298035705
181	24	0	A_24_P263284	NM_052896	0,473549187	5,413182161	2,607287767	0,042533029	0,843980537	-4,29806958
161	71	0	A_24_P6889	NM_006099	-0,506294568	6,656674261	-2,606003221	0,042604009	0,843980537	-4,298269591
12	8	0	A_32_P804837	ENST00000540779	0,82615419	5,107291649	2,6059223	0,042608484	0,843980537	-4,298282195
138	77	0	A_24_P930975	NM_001115152	-0,497610261	5,746954723	-2,60556716	0,042628133	0,843980537	-4,298337512
301	83	0	A_23_P393531	NM_004027	-0,395020293	6,991414922	-2,605487047	0,042632566	0,843980537	-4,298349992
406	11	0	A_32_P133090	BG216262	0,645283925	6,75602191	2,60548042	0,042632933	0,843980537	-4,298351024
430	82	0	A_23_P56567	NM_024775	0,343438764	11,7591694	2,603835965	0,042724052	0,843980537	-4,298607283
7	76	0	A_23_P129225	NM_002420	-0,430539236	5,942620297	-2,603405104	0,042747961	0,843980537	-4,298674453
40	82	0	A_23_P131887	U35612	-0,464973543	8,352654641	-2,602992839	0,042770851	0,843980537	-4,298738735
17	66	0	A_23_P135669	NM_030958	-0,555747316	6,409049253	-2,602645722	0,042790134	0,843980537	-4,298792867
325	83	0	A_24_P298143	A_24_P298143	-0,442217026	6,113261262	-2,602586984	0,042793398	0,843980537	-4,298802028
186	5	0	A_32_P123701	AY927488	0,724768115	6,001308292	2,602453555	0,042800813	0,843980537	-4,298822839
443	11	0	A_23_P359540	NM_003540	0,387516068	9,448826399	2,601770747	0,042838782	0,843980537	-4,298929352
157	62	0	A_24_P911327	A_24_P911327	-0,362185605	6,982303726	-2,601183422	0,04287147	0,843980537	-4,299020995
338	29	0	A_23_P434809	NM_002964	0,469272613	6,317159043	2,600440929	0,042912833	0,843980537	-4,29913688
263	77	0	A_23_P57588	NM_016426	-0,347847888	10,02662703	-2,600398677	0,042915188	0,843980537	-4,299143475
335	9	0	A_32_P129540	NR_037629	0,370326388	7,059253858	2,5989968	0,042993405	0,843980537	-4,299362373
97	21	0	A_24_P12397	NM_018965	0,56739796	6,184819205	2,598042776	0,043046721	0,843980537	-4,299511412
232	49	0	A_24_P409361	A_24_P409361	0,377634153	6,766343025	2,597937014	0,043052636	0,843980537	-4,299527937
89	79	0	A_23_P215454	NM_001278939	-0,781721804	7,533449818	-2,597445052	0,043080162	0,843980537	-4,299604818
304	15	0	A_32_P197109	A_32_P197109	0,693625639	6,580258887	2,59680922	0,043115765	0,843980537	-4,299704204
39	65	0	A_24_P687582	NM_152293	-0,518487008	6,595587215	-2,596789622	0,043116863	0,843980537	-4,299707268
166	60	0	A_24_P276983	NM_006374	-0,390924029	9,952284084	-2,596306439	0,043143941	0,843980537	-4,299782812
369	9	0	A_23_P218977	NM_014423	0,348732079	8,573082801	2,596067424	0,043157342	0,843980537	-4,299820186
453	11	0	A_32_P136871	THC2712372	-0,484064233	6,931897956	-2,595917496	0,04316575	0,843980537	-4,299843632
485	45	0	A_24_P464540	NR_027055	0,631635619	6,45445195	2,595751814	0,043175044	0,843980537	-4,299869543
135	19	0	A_23_P337168	NM_153013	0,390027893	7,971914894	2,595500301	0,043189157	0,843980537	-4,299908881
39	29	0	A_23_P203841	NM_013449	-0,485535924	7,996342803	-2,594678647	0,043235296	0,843980537	-4,300037419
488	46	0	A_23_P145134	NM_007045	0,459076295	8,338809451	2,594615539	0,043238842	0,843980537	-4,300047294
181	80	0	A_23_P207160	NM_022558	-0,832956464	6,474098782	-2,593861698	0,043281223	0,843980537	-4,300165264
214	81	0	A_23_P114649	NM_021933	-0,55412831	8,954806486	-2,593827396	0,043283153	0,843980537	-4,300170633
354	28	0	A_23_P368366	NM_207332	0,532911111	7,78066704	2,592935126	0,043333377	0,843980537	-4,300310315
71	59	0	A_24_P316619	U25433	-0,518037451	5,688175469	-2,59277729	0,043342268	0,843980537	-4,300335029
291	19	0	A_23_P131183	NM_001485	0,483744647	7,164774749	2,592664157	0,043348641	0,843980537	-4,300352744
469	37	0	A_24_P24183	ENST00000441052	0,456914014	6,624814958	2,592651489	0,043349355	0,843980537	-4,300354728
137	77	0	A_24_P58740	NR_030627	-0,412330006	7,269054149	-2,592434736	0,04336157	0,843980537	-4,300388672
104	75	0	A_23_P82795	NR_040035	-0,577050642	6,208936448	-2,592230051	0,043373108	0,843980537	-4,300420728
95	54	0	A_32_P128280	THC2662703	-0,581307375	5,417939516	-2,592140662	0,043378148	0,843980537	-4,300434728
207	62	0	A_32_P222355	THC2522381	-0,569531529	6,573429401	-2,591433666	0,043418032	0,843980537	-4,300545478
217	83	0	A_24_P285768	NM_014674	-0,467250386	7,443955122	-2,591293577	0,043425939	0,843980537	-4,300567426
183	79	0	A_32_P149676	THC2568627	-0,41530063	7,497692135	-2,591075156	0,043438271	0,843980537	-4,30060165
229	18	0	A_24_P272748	NM_001144956	0,620768418	8,062190375	2,588414324	0,043588805	0,843980537	-4,301018808
384	19	0	A_32_P109214	NM_001004306	0,489785711	7,299278479	2,588031141	0,043610529	0,843980537	-4,30107892
430	55	0	A_23_P424305	XM_005255415	0,354468081	11,38369702	2,587844576	0,043621111	0,843980537	-4,30110819
163	77	0	A_24_P153869	ENST00000518265	-0,533302628	6,745291539	-2,587804881	0,043623362	0,843980537	-4,301114418
147	59	0	A_24_P501634	NM_078476	-0,69009961	5,755170245	-2,587543252	0,043638207	0,843980537	-4,30115547
473	18	0	A_23_P2942	NM_022123	-0,675702374	6,233614749	-2,587462074	0,043642814	0,843980537	-4,301168209
12	6	0	A_32_P200773	THC2622100	0,47296838	7,414787389	2,587284925	0,043652869	0,843980537	-4,301196008
297	49	0	A_32_P411592	NM_052897	-0,46136454	10,90504813	-2,587058148	0,043665745	0,843980537	-4,301231599
133	85	0	A_24_P924739	A_24_P924739	-0,564082158	5,823070641	-2,586905703	0,043674402	0,843980537	-4,301255525
132	9	0	A_24_P340128	NM_178129	0,640531841	5,580687153	2,585480727	0,043755419	0,843980537	-4,301479251
272	62	0	A_23_P38978	CA312034	-0,768404098	6,586325001	-2,585468384	0,043756121	0,843980537	-4,301481189
291	40	0	A_32_P13417	ENST00000460022	-0,502185745	6,093217225	-2,585034551	0,043780819	0,843980537	-4,301549328
168	2	0	A_23_P25813	NM_006683	0,415399663	5,181268606	2,583950004	0,043842627	0,843980537	-4,301719722
314	37	0	A_24_P179467	NM_005071	0,705925883	6,206387531	2,583611107	0,04386196	0,843980537	-4,301772982
93	75	0	A_23_P389496	NM_173536	-0,844446736	5,171365922	-2,583555696	0,043865122	0,843980537	-4,30178169
291	11	0	A_32_P23145	A_32_P23145	0,717752921	6,749185494	2,583514736	0,043867459	0,843980537	-4,301788128
83	73	0	A_23_P253389	NM_003040	-0,498060116	8,599914853	-2,583019417	0,043895736	0,843980537	-4,301865986
93	31	0	A_24_P233786	NM_052966	0,364047141	8,636692549	2,582886575	0,043903323	0,843980537	-4,30188687
89	72	0	A_23_P28857	NM_018556	-0,678648479	6,211972797	-2,582731834	0,043912162	0,843980537	-4,301911198
406	35	0	A_23_P250173	NM_020872	0,501773743	7,351034021	2,58269932	0,04391402	0,843980537	-4,30191631
4	49	0	A_23_P99044	NM_033448	-0,408565989	5,904717726	-2,582370539	0,043932808	0,843980537	-4,301968006
492	76	0	A_24_P15325	AJ301560	-0,5757445	5,966856035	-2,582296195	0,043937058	0,843980537	-4,301979697
215	16	0	A_24_P920016	NM_001145191	-0,41626877	6,510574349	-2,582092177	0,043948722	0,843980537	-4,30201178
168	13	0	A_32_P198523	NM_173651	0,923070517	6,000694065	2,581977735	0,043955266	0,843980537	-4,302029778

523	58	0	A_23_P205293	NM_018199	-0,401528793	6,320650121	-2,581964594	0,043956018	0,843980537	-4,302031844
138	62	0	A_24_P247576	A_24_P247576	-0,443826344	6,103217974	-2,581797615	0,043965569	0,843980537	-4,302058106
154	58	0	A_23_P377191	A_23_P377191	-0,461260484	6,198733065	-2,580983768	0,044012151	0,843980537	-4,302186131
226	5	0	A_23_P151653	NM_080649	-0,357723161	9,437670859	-2,580711383	0,044027753	0,843980537	-4,302228988
19	78	0	A_24_P58177	NM_001286473	-0,607802928	7,33254605	-2,580618617	0,044033068	0,843980537	-4,302243585
55	76	0	A_23_P39925	NM_003494	-0,3560842	7,77105922	-2,580430173	0,044043867	0,843980537	-4,302273239
362	63	0	A_32_P234954	BX099788	0,60113039	6,293849706	2,580084285	0,044063696	0,843980537	-4,302327675
111	79	0	A_23_P312415	NM_052866	-0,880253189	6,854819629	-2,579957348	0,044070975	0,843980537	-4,302347654
217	3	0	A_23_P98147	NM_001308	0,480508586	6,20665578	2,57958073	0,04409258	0,843980537	-4,302406938
194	18	0	A_23_P13753	NM_006163	0,477739422	6,673996967	2,579529679	0,04409551	0,843980537	-4,302414974
462	58	0	A_23_P35148	NM_005645	0,393271342	8,220775879	2,579176148	0,044115803	0,843980537	-4,302470633
169	54	0	A_24_P307498	NM_198493	-0,459453582	6,554071066	-2,578596681	0,044149085	0,843980537	-4,30256188
243	1	0	A_24_P643601	ENST00000525833	0,52250779	6,305215611	2,578434199	0,044158423	0,843980537	-4,302587469
111	63	0	A_23_P165541	ENST00000486293	-0,381308146	7,672834752	-2,578187812	0,044172586	0,843980537	-4,302626276
137	2	0	A_23_P47340	NM_020693	0,60748827	6,520513769	2,578046528	0,04418071	0,843980537	-4,30264853
483	37	0	A_24_P281264	A_24_P281264	0,463292763	5,993613231	2,57761355	0,044205615	0,843980537	-4,302716739
205	33	0	A_23_P312285	NM_153289	0,337743534	5,838413238	2,576826517	0,044250925	0,843980537	-4,302840753
248	11	0	A_23_P39265	NM_014400	0,523143568	7,1392994	2,576737234	0,044256069	0,843980537	-4,302854824
50	34	0	A_23_P28011	NM_033196	-0,48639262	5,938393973	-2,576532988	0,044267837	0,843980537	-4,302887016
457	18	0	A_23_P346982	NM_173666	-0,525354945	6,214931853	-2,576253458	0,044283948	0,843980537	-4,302931076
41	48	0	A_24_P943301	NM_001080471	-0,474079856	6,338557098	-2,575945688	0,044301694	0,843980537	-4,302979594
89	74	0	A_32_P8156	NM_173554	-0,625007968	6,990742965	-2,575945098	0,044301728	0,843980537	-4,302979687
229	84	0	A_24_P57837	BG195969	0,407220649	12,71363751	2,575875281	0,044305755	0,843980537	-4,302990694
488	13	0	A_24_P243086	NM_144635	-0,37667691	7,043496779	-2,575232863	0,044342826	0,843980537	-4,303091989
3	51	0	A_32_P213091	NM_001145205	-0,459329477	6,317375843	-2,57506214	0,044352683	0,843980537	-4,303118913
226	83	0	A_24_P256219	AF055376	-0,756041168	6,079293238	-2,574764593	0,044369868	0,843980537	-4,303165842
24	9	0	A_23_P417100	NM_030976	0,804736965	6,071196909	2,574715107	0,044372727	0,843980537	-4,303173647
171	15	0	A_24_P106297	NM_014324	0,626525323	7,766126776	2,57420053	0,044402466	0,843980537	-4,303254821
101	1	0	A_23_P353035	NM_001553	0,436847513	6,282452821	2,57419699	0,04440267	0,843980537	-4,303255379
453	50	0	A_23_P426809	NM_198236	-0,367594541	8,187212457	-2,573840286	0,044423298	0,843980537	-4,303311658
493	85	0	A_23_P205159	NM_005358	-0,774175332	6,989176494	-2,573674195	0,044432906	0,843980537	-4,303337866
119	57	0	A_23_P88559	NM_000236	-0,510314328	6,377146282	-2,573312288	0,04445385	0,843980537	-4,303394978
216	30	0	A_32_P112293	ENST00000530735	0,565536409	5,449989703	2,572939817	0,044475417	0,843980537	-4,303453766
255	18	0	A_24_P348594	NM_006953	0,520782888	6,415415027	2,571955319	0,044532473	0,843980537	-4,303609194
514	30	0	A_24_P923789	A_24_P923789	0,348629563	6,285488625	2,571771232	0,044543151	0,843980537	-4,303638264
30	55	0	A_23_P15832	NM_014566	-0,459347212	5,751009694	-2,57160799	0,044552622	0,843980537	-4,303664043
15	48	0	A_24_P204848	AF222858	-0,645154708	4,905225268	-2,571366072	0,044566661	0,843980537	-4,303702251
20	24	0	A_23_P339818	NM_183376	0,752513911	8,112709858	2,57112039	0,044580923	0,843980537	-4,303741057
398	55	0	A_32_P117977	A_32_P117977	-0,471764842	6,844406158	-2,571018288	0,044586852	0,843980537	-4,303757185
197	59	0	A_24_P274814	NM_030984	-0,349744991	6,583881135	-2,570982698	0,044588919	0,843980537	-4,303762807
157	34	0	A_23_P16384	NM_139176	0,378557997	5,947183518	2,570670306	0,044607065	0,843980537	-4,303812158
42	40	0	A_24_P187874	AK098031	-0,517453705	6,128011432	-2,570302371	0,044628447	0,843980537	-4,303870292
493	70	0	A_24_P62860	NM_005843	-0,524652568	6,446055246	-2,570196822	0,044634583	0,843980537	-4,30388697
155	48	0	A_23_P8906	NM_013437	0,455169075	8,469581376	2,570107153	0,044639796	0,843980537	-4,30390114
466	24	0	A_32_P214274	NM_145027	-0,401279416	6,283973304	-2,569378011	0,044682214	0,843980537	-4,304016379
35	77	0	A_24_P399606	NM_000435	-0,590471392	6,971715195	-2,569149704	0,044695504	0,843980537	-4,304052469
146	38	0	A_32_P205944	NM_001040616	0,692921388	8,139223654	2,567828754	0,044772484	0,843980537	-4,304261346
413	51	0	A_24_P564761	ENST00000517345	1,015445339	6,099002988	2,567023243	0,044819496	0,843980537	-4,304388773
242	13	0	A_23_P207003	NM_004574	0,534850027	7,049008697	2,566475827	0,044851475	0,843980537	-4,304475395
83	61	0	A_32_P29965	NM_005365	-0,448004191	5,712950786	-2,566313452	0,044860966	0,843980537	-4,304501092
157	16	0	A_24_P371782	NM_000735	0,541204537	6,347716595	2,566181698	0,044868668	0,843980537	-4,304521945
228	22	0	A_23_P47077	NM_004281	0,666347124	7,352023037	2,565889015	0,044885783	0,843980537	-4,304568271
216	10	0	A_24_P659836	NM_001123225	0,406426264	8,510041626	2,565661192	0,04489911	0,843980537	-4,304604335
213	21	0	A_24_P85123	AK024440	0,708712975	7,42624559	2,565468563	0,044910381	0,843980537	-4,304634831
486	22	0	A_32_P166760	A_32_P166760	-0,352534018	6,308608027	-2,564908403	0,044943176	0,843980537	-4,304723525
461	47	0	A_23_P160318	NM_001856	0,473372868	8,722102566	2,564696662	0,044955579	0,843980537	-4,304757056
374	56	0	A_23_P42784	NM_032944	0,585620663	5,954832245	2,564391357	0,044973469	0,843980537	-4,304805409
265	52	0	A_24_P308506	NM_016347	0,620125463	6,60250625	2,56435007	0,044975889	0,843980537	-4,304811949
480	66	0	A_23_P211227	NM_133635	-0,386727104	7,483813704	-2,564135936	0,044988442	0,843980537	-4,304845867
404	69	0	A_32_P228570	ENST00000585184	0,549197314	7,382714783	2,563487576	0,045026474	0,843980537	-4,304948582
487	38	0	A_32_P201958	NM_014053	0,351187664	9,170926032	2,562804377	0,045066586	0,843980537	-4,305056846
437	37	0	A_23_P213014	NM_001001290	0,449451902	5,918563632	2,56259124	0,045079108	0,843980537	-4,305090627
161	84	0	A_32_P470868	NM_182542	-1,093092462	5,979549762	-2,562454819	0,045087124	0,843980537	-4,305112251
529	27	0	A_32_P886589	NM_175895	0,500780969	6,316635654	2,562422393	0,04508903	0,843980537	-4,30511739
203	9	0	A_23_P313652	NM_178813	0,807080313	6,08294398	2,56235032	0,045093266	0,843980537	-4,305128815
295	22	0	A_24_P177585	NM_182625	0,479750034	7,526872521	2,562036446	0,045111719	0,843980537	-4,305178572
465	39	0	A_24_P185516	ENST00000594337	1,177659265	6,394321215	2,561683181	0,045132497	0,843980537	-4,305234582
117	84	0	A_24_P317907	NM_001034954	-0,481415252	6,929588804	-2,561299772	0,04515506	0,843980537	-4,305295379
435	37	0	A_32_P34868	THC2764893	0,732743106	6,3765544	2,561109095	0,045166286	0,843980537	-4,305325619
169	80	0	A_23_P17420	NM_003657	-0,891050599	6,638041436	-2,5610783	0,045168099	0,843980537	-4,305330503
378	75	0	A_23_P86493	NM_006562	1,476746467	9,746084424	2,560810599	0,045183865	0,843980537	-4,305372961
497	18	0	A_23_P50031	NM_002550	0,441159545	5,268920998	2,560520165	0,045200976	0,843980537	-4,305419031
22	77	0	A_23_P142738	NM_152390	-0,426859944	6,589780473	-2,560176452	0,045221235	0,843980537	-4,305473559
32	74	0	A_23_P97889	NM_013274	-0,380889295	8,727135488	-2,559900212	0,045237524	0,843980537	-4,305517388
154	20	0	A_23_P376488	NM_000594	0,519536849	7,34160452	2,559438331	0,045264774	0,843980537	-4,305590683
351	13	0	A_23_P115478	NM_013358	-0,421608168	6,09761895	-2,558593636	0,045314655	0,843980537	-4,30572476
161	81	0	A_23_P347623	NM_019891	-1,114412888	6,995951738	-2,557382863	0,045386256	0,843980537	-4,305917023
129	69	0	A_32_P84714	A_32_P84714	-0,718868252	6,501881605	-2,557191778	0,045397567	0,843980537	-4,305947374
102	9	0	A_24_P918436	NM_152221	-0,407919533	8,818686259	-2,557139154	0,045400683	0,843980537	-4,305955733
318	10	0	A_24_P417439	NR_033837	0,618125125	5,427119823	2,556579252	0,045433846	0,843980537	-4,306044682
29	81	0	A_24_P247303	A_24_P247303	-0,533361775	6,155084362	-2,556434115	0,045442446	0,843980537	-4,306067743
245	82	0	A_24_P294343	NM_024616	-0,587970488	6,403021394	-2,556269871	0,045452181	0,843980537	-4,306093841
381	47	0	A_32_P189790	NR_027134	0,548531289	5,903487081	2,555998221	0,045468287	0,843980537	-4,306137009
234	28	0	A_23_P146233	NM_000237	0,338810411	7,266248892	2,555814167	0,045479203	0,843980537	-4,30616626
323	83	0	A_24_P221745	ENST00000424010	-0,762674013	6,168579563	-2,555772744	0,04548166	0,843980537	-4,306172843
22	79	0	A_24_P365526	NM_002110	-0,636410535	6,335944493	-2,555047217	0,04552472	0,843980537	-4,306288171
129	4	0	A_32_P305500	NM_004770	0,419121139	6,054844244	2,554997846	0,045527652	0,843980537	-4,30629602
422	12	0	A_23_P118722	NM_001671	0,46521573	8,466301972	2,554994646	0,045527842	0,843980537	-4,306296529
462	27	0	A_24_P55496	NM_053001	0,492316914	6,452623049	2,554872306	0,045535108	0,843980537	-4,30631598
40	64	0	A_24_P328872	NM_012073	-0,559971766	5,999451937	-2,554025779	0,045585416	0,843980537	-4,306450593
255	22	0	A_24_P931443	NM_003485	0,684597033	7,725067937	2,553851071	0,045595806	0,843980537	-4,30647838
190	20	0	A_24_P917402	NR_024492	-0,339165378	6,849618188	-2,553839042	0,045596522	0,843980537	-4,306480294
402	34	0	A_32_P117636	AK125176	0,748443112	5,760747	2,553765465	0,045600899	0,843980537	-4,306491997
217	34	1	DCP_1_11	DCP_1_11	0,416237934	5,576443793	2,553347884	0,045625746	0,843980537	-4,306558424

246	66	0	A_23_P124559	NM_031300	-0,33741134	9,122930224	-2,552583999	0,045671237	0,843980537	-4,306679967
516	23	0	A_23_P326760	NM_015460	0,612510053	8,881694079	2,551996852	0,045706236	0,843980537	-4,306773415
71	36	0	A_23_P23575	NM_014437	-0,357070454	9,88577901	-2,551530652	0,045734046	0,843980537	-4,306847629
46	41	0	A_32_P111769	THC2624682	-0,57378989	5,539552498	-2,55042912	0,045799828	0,843980537	-4,307023036
156	21	0	A_23_P351203	NM_172370	0,544575603	5,533073172	2,549954599	0,045828196	0,843980537	-4,307098622
531	66	0	A_24_P302685	NM_015320	-0,423993027	6,476399985	-2,549689478	0,045844054	0,843980537	-4,307140859
115	27	0	A_24_P245379	NM_002575	0,359679383	6,305928036	2,549599339	0,045849447	0,843980537	-4,30715522
328	82	0	A_24_P126902	A_24_P126902	0,364317537	8,432036075	2,548773159	0,045898909	0,843980537	-4,307286874
84	68	0	A_24_P140621	NM_024565	-0,412302489	6,844572349	-2,547938426	0,04594894	0,843980537	-4,307419935
471	43	0	A_24_P126741	A_24_P126741	0,42762388	6,720898008	2,547701865	0,045963129	0,843980537	-4,307457652
259	21	0	A_32_P57844	A_32_P57844	0,507215967	6,116984725	2,547074615	0,046000776	0,843980537	-4,307557678
341	77	0	A_32_P50508	A_32_P50508	-0,391966412	6,947318519	-2,546925085	0,046009755	0,843980537	-4,307581527
213	20	0	A_23_P102917	NM_033171	0,765142947	5,989317248	2,54613542	0,046057206	0,843980537	-4,307707495
517	58	0	A_23_P318938	NM_153022	0,412462833	5,567751072	2,546025181	0,046063835	0,843980537	-4,307725084
48	84	0	A_23_P81811	NM_080604	-0,416987033	9,840469392	-2,545325751	0,046105914	0,843980537	-4,307836696
136	77	0	A_23_P72663	NM_001012393	-0,567353327	6,592479257	-2,54508423	0,046120453	0,843980537	-4,307875244
35	53	0	A_24_P923553	A_24_P923553	-0,52129775	5,406764263	-2,543912666	0,046191053	0,843980537	-4,308062285
180	70	0	A_24_P824005	THC2652793	-0,64795168	5,867031093	-2,54384226	0,046195299	0,843980537	-4,308073528
119	52	0	A_23_P136125	NM_005141	-0,345820812	4,670073282	-2,543553515	0,046212719	0,843980537	-4,308119642
188	84	0	A_24_P387321	NM_016264	-0,449606246	7,052311039	-2,542494526	0,046276665	0,843980537	-4,308288809
14	39	0	A_24_P320033	NM_005191	-0,455708475	5,568709638	-2,54233388	0,046286374	0,843980537	-4,308314478
158	6	0	A_23_P409648	ENST00000458341	0,842382534	5,549783321	2,542178202	0,046295785	0,843980537	-4,308339354
509	20	0	A_23_P92562	NM_000673	0,469900714	5,278585131	2,541951348	0,046309502	0,843980537	-4,308375606
229	61	0	A_23_P20894	NM_024757	-0,501351483	7,134935909	-2,54163953	0,046328363	0,843980537	-4,308425442
133	17	0	A_24_P902073	AL049331	0,51436561	5,841856912	2,541077943	0,046362354	0,843980537	-4,308515212
517	20	0	A_24_P791169	AK000115	-0,481496239	5,649757277	-2,540984276	0,046368026	0,843980537	-4,308530186
156	34	0	A_24_P264970	AK091511	0,456095287	5,517643206	2,540325139	0,046407959	0,843980537	-4,308635579
102	4	0	A_23_P156890	NM_003206	0,523434497	6,280924417	2,540297293	0,046409647	0,843980537	-4,308640032
6	58	0	A_24_P289106	ENST00000606872	-0,370194246	8,185236899	-2,540160892	0,046417916	0,843980537	-4,308661846
184	85	0	A_23_P152262	NM_004413	-0,506510318	6,934917339	-2,540056505	0,046424245	0,843980537	-4,30867854
453	15	0	A_23_P373598	NM_002360	-0,476440406	7,598785808	-2,539904794	0,046433445	0,843980537	-4,308702804
137	65	0	A_32_P125771	NM_015668	-0,732570544	5,164607592	-2,539686558	0,046446683	0,843980537	-4,308737711
532	48	0	A_23_P103511	NM_001085375	-0,526770742	7,155601847	-2,539544407	0,046455308	0,843980537	-4,30876045
48	60	0	A_24_P464238	A_24_P464238	-1,037505953	5,821112666	-2,53888051	0,046495611	0,843980537	-4,308866665
133	18	0	A_24_P115621	NM_004096	0,347253497	10,13339629	2,538368372	0,046526727	0,843980537	-4,308948619
128	13	0	A_24_P352116	NR_003672	0,397825924	7,575824254	2,538152481	0,04653985	0,843980537	-4,308983172
269	35	0	A_24_P92952	NM_006015	-0,426152945	10,16357157	-2,53810484	0,046542747	0,843980537	-4,308990797
153	14	0	A_23_P378574	NM_173523	0,475961345	5,484589885	2,537161741	0,046600126	0,843980537	-4,309141775
203	13	0	A_23_P11103	NM_005448	0,442837529	5,982999366	2,536968445	0,046611896	0,843980537	-4,309172726
216	64	0	A_32_P66297	THC2638666	-0,500516287	6,589821648	-2,536801301	0,046622076	0,843980537	-4,309199491
216	20	0	A_24_P919256	A_24_P919256	0,460732526	6,648495298	2,536423141	0,046645116	0,843980537	-4,309260054
265	60	0	A_23_P63254	NM_006142	0,336095518	7,879196743	2,535870253	0,046678824	0,843980537	-4,309348617
475	23	0	A_23_P201248	NM_052934	0,43580542	5,92503449	2,535863629	0,046679228	0,843980537	-4,309349678
264	9	0	A_24_P309521	NM_000890	0,55440473	6,009011889	2,534166883	0,046782839	0,843980537	-4,309621587
94	57	0	A_24_P6370	NM_178550	-0,364667309	5,798372948	-2,533069453	0,046849983	0,843980537	-4,309797551
301	79	0	A_24_P305570	NM_018993	-0,418600533	6,488502867	-2,532903578	0,046860141	0,843980537	-4,309824155
170	69	0	A_24_P251599	NM_001234	-0,738894514	6,623058189	-2,532209813	0,04690265	0,843980537	-4,309935441
131	3	0	A_32_P116399	NM_006852	0,596368668	6,957063108	2,532029621	0,046913698	0,843980537	-4,309964351
180	79	0	A_24_P324054	ENST00000447423	-0,394213921	5,997427289	-2,531689825	0,046934539	0,843980537	-4,310018872
207	31	0	A_32_P20686	ENST00000521148	0,335973191	6,552277572	2,530478794	0,047008897	0,843980537	-4,310213247
89	65	0	A_23_P106933	NM_052956	-0,479813278	5,751727159	-2,530241711	0,047023468	0,843980537	-4,31025131
145	72	0	A_24_P677823	ENST00000585484	-0,559508487	5,255500148	-2,530086304	0,047033023	0,843980537	-4,310276263
236	83	0	A_24_P90900	NM_001907	-0,733026529	6,845180194	-2,529405219	0,04707492	0,843980537	-4,310385637
242	38	0	A_23_P3767	NM_173215	0,493320747	7,805804323	2,52908305	0,047094752	0,843980537	-4,310437384
506	3	0	A_32_P23989	NR_038381	-0,335197164	5,975686504	-2,528605796	0,047124147	0,843980537	-4,310514053
272	73	0	A_23_P13033	NM_002896	-0,404056845	10,55687854	-2,528564426	0,047126696	0,843980537	-4,310520699
38	27	0	A_24_P926306	AF116714	0,362830034	4,734261705	2,528394017	0,047137197	0,843980537	-4,310548079
334	13	0	A_23_P147869	NM_015312	0,400419581	8,798574551	2,528165929	0,047151257	0,843980537	-4,310584728
16	31	0	A_32_P896811	AF279780	0,513563102	5,334515089	2,528029925	0,047159643	0,843980537	-4,310606583
186	16	0	A_24_P919668	A_24_P919668	0,502176355	6,055386768	2,527685713	0,047180873	0,843980537	-4,310661901
137	1	0	A_23_P20075	NM_013389	0,411988214	7,118721001	2,52749472	0,047192657	0,843980537	-4,310692598
390	65	0	A_23_P8702	NM_002652	0,802482358	5,612208965	2,526978452	0,047224527	0,843980537	-4,310775587
320	67	0	A_24_P122746	NM_022834	0,427091607	6,641924204	2,526934759	0,047227225	0,843980537	-4,310782611
52	77	0	A_24_P936010	AY358253	-1,069607471	6,152941644	-2,526288836	0,047267133	0,843980537	-4,310886468
191	72	0	A_32_P5995	THC2543133	-0,487874771	6,514853945	-2,525253967	0,047331148	0,843980537	-4,311052917
246	23	0	A_23_P43415	NM_000197	0,404483851	5,662583649	2,525195677	0,047334756	0,843980537	-4,311062294
273	3	0	A_32_P208964	A_32_P208964	0,468735603	6,547577861	2,525122062	0,047339314	0,843980537	-4,311074137
337	47	0	A_32_P127956	THC2746190	-0,545501736	6,61377442	-2,525102897	0,0473405	0,843980537	-4,311077221
420	17	0	A_23_P54079	NM_017807	0,510822716	8,447720138	2,525071282	0,047342458	0,843980537	-4,311082307
169	15	0	A_23_P1322	NR_073125	0,436949896	7,281259061	2,524760399	0,047361711	0,843980537	-4,311132327
460	29	0	A_24_P572229	A_24_P572229	0,423299448	7,064413197	2,524728077	0,047363714	0,843980537	-4,311137527
311	65	0	A_23_P259251	NM_012281	0,410447536	6,280635708	2,52464658	0,047368762	0,843980537	-4,311150641
485	32	0	A_24_P6850	A_24_P6850	0,667310193	6,737636024	2,524415635	0,047383073	0,843980537	-4,311187805
478	43	0	A_24_P389916	NM_005512	0,374591548	7,76183022	2,524256349	0,047392946	0,843980537	-4,311213439
78	70	0	A_23_P311050	NM_152590	-0,66532207	6,152996736	-2,523771649	0,047423002	0,843980537	-4,311291453
35	41	0	A_23_P159865	NM_145052	-0,702550037	6,49550523	-2,523637847	0,047431302	0,843980537	-4,311312991
231	20	0	A_23_P391906	XM_005266817	0,49809734	6,834378884	2,523538418	0,047437472	0,843980537	-4,311328997
473	46	0	A_24_P249784	NM_178040	0,532426325	7,843444579	2,523187895	0,047459227	0,843980537	-4,311385429
89	52	0	A_23_P140074	NM_003891	-0,488562834	6,315369162	-2,522860896	0,047479532	0,843980537	-4,311438081
98	34	0	A_23_P147046	NM_005233	0,615300432	6,15634709	2,522656945	0,047492201	0,843980537	-4,311470924
219	23	0	A_24_P929499	AY037163	0,71814944	5,386167072	2,522466028	0,047504064	0,843980537	-4,31150167
200	10	0	A_24_P699733	AK021745	-0,333932202	6,500028634	-2,522286363	0,04751523	0,843980537	-4,311530606
53	45	0	A_24_P839296	XM_005246047	-0,562328372	6,268441998	-2,522001645	0,047532932	0,843980537	-4,311576466
202	22	0	A_23_P80883	NM_181780	0,556185976	6,118932474	2,521580323	0,047559139	0,843980537	-4,311644339
143	83	0	A_32_P366349	NM_001113382	-0,979122832	6,284971556	-2,521155258	0,047585595	0,843980537	-4,311712825
521	73	0	A_32_P67623	NM_017848	0,37584602	9,270644043	2,520916298	0,047600474	0,843980537	-4,311751332
449	69	0	A_23_P73511	NM_001654	-0,329652867	8,588588848	-2,520208108	0,047644601	0,843980537	-4,311865472
379	69	0	A_23_P215435	NM_001257190	-0,566498613	6,767921829	-2,520030251	0,04765569	0,843980537	-4,311894143
180	18	0	A_24_P630676	A_24_P630676	0,575145927	5,722058867	2,519693032	0,047676723	0,843980537	-4,311948508
388	61	0	A_24_P24060	NM_032498	0,635826586	6,161621788	2,519326932	0,047699568	0,843980537	-4,312007538
470	47	0	A_23_P386973	NM_172238	0,440950828	6,134633852	2,518862371	0,047728574	0,843980537	-4,312082455
341	69	0	A_23_P370035	NM_181340	0,388452039	9,596688004	2,518699321	0,047738758	0,843980537	-4,312108753
49	70	0	A_23_P6714	NM_020654	-0,862563168	6,34041763	-2,518398816	0,047757535	0,843980537	-4,312157224
247	49	0	A_23_P363313	NM_153357	0,742085987	6,988800016	2,517102358	0,047838635	0,843980537	-4,312366408

366	4	0	A_23_P111593	NM_004227	0,383245487	8,288088964	2,517041885	0,047842421	0,843980537	-4,312376168
121	83	0	A_23_P27460	A_23_P27460	-0,871851298	7,222883553	-2,516224953	0,047893604	0,843980537	-4,312508039
340	10	0	A_24_P612200	ENST00000412378	0,474673076	7,562137952	2,516095244	0,047901736	0,843980537	-4,312528981
96	83	0	A_23_P254434	NM_006605	-0,670348039	6,674178757	-2,515523302	0,047937611	0,843980537	-4,312621334
388	16	0	A_23_P124534	NM_001253732	0,662312187	5,337716727	2,515481699	0,047940221	0,843980537	-4,312628053
518	76	0	A_24_P767725	TCONS_l2_00008906	-0,455788778	6,339011949	-2,515003021	0,04797027	0,843980537	-4,312705364
269	68	0	A_23_P88278	NM_020366	-0,478965583	6,576416612	-2,514794056	0,047983394	0,843980537	-4,312739119
295	61	0	A_23_P100092	NM_152455	0,350814519	8,617844996	2,514712601	0,04798851	0,843980537	-4,312752277
381	39	0	A_24_P252934	NM_000482	0,850388801	5,83964598	2,514334881	0,048012245	0,843980537	-4,3128133
16	65	0	A_24_P916979	A_24_P916979	-0,45866758	6,484733764	-2,514319373	0,04801322	0,843980537	-4,312815806
22	49	0	A_24_P332064	ENST00000430548	-0,603053383	7,941174849	-2,513597145	0,048058639	0,843980537	-4,312932512
452	56	0	A_24_P270235	NM_001759	0,486750403	6,221337864	2,513259179	0,048079908	0,843980537	-4,312987135
198	81	0	A_24_P397903	NM_016010	-1,063961016	5,876026478	-2,512813968	0,048107942	0,843980537	-4,313059104
441	39	0	A_24_P201360	NM_203380	0,385687994	6,238227813	2,512187484	0,04814742	0,843980537	-4,313160397
26	35	0	A_23_P27096	NM_005022	-0,402233693	12,29144124	-2,512180332	0,048147871	0,843980537	-4,313161553
53	21	0	A_24_P250333	NM_004596	-0,348158248	11,70933561	-2,511907008	0,048165105	0,843980537	-4,313205753
139	67	0	A_24_P246963	A_24_P246963	-0,380435935	8,327331112	-2,511472496	0,048192517	0,843980537	-4,313276029
402	48	0	A_23_P320159	ENST00000490617	0,468446886	7,99190494	2,511430275	0,048195182	0,843980537	-4,313282859
121	8	0	A_23_P80918	NM_001179	0,522122261	6,264834304	2,511251448	0,048206469	0,843980537	-4,313311785
9	72	0	A_24_P238131	NM_015551	-0,500983471	6,588820064	-2,510538484	0,048251497	0,843980537	-4,313427133
233	60	0	A_23_P37514	NM_015492	0,350587082	9,128575218	2,510476461	0,048255416	0,843980537	-4,313437169
311	12	0	A_32_P220938	NM_006916	0,591356659	7,448507277	2,510122881	0,048277766	0,843980537	-4,313494387
157	33	0	A_23_P422245	NM_173665	0,51389096	5,291031768	2,509392707	0,048323955	0,843980537	-4,313612572
158	25	0	A_24_P255100	NM_001010940	0,660541426	5,791671395	2,50918584	0,048337049	0,843980537	-4,313646061
69	12	0	A_24_P936767	NM_005082	-0,400110822	7,901515641	-2,508909712	0,048354533	0,843980537	-4,313690768
115	60	0	A_32_P17504	ENST00000432148	-0,64755693	6,362023172	-2,508829573	0,048359609	0,843980537	-4,313703743
285	41	0	A_24_P354429	NM_002159	0,442463583	6,016176323	2,5082324	0,048397449	0,843980537	-4,313800447
348	15	0	A_32_P233688	NM_178456	0,427705841	5,249941197	2,507594532	0,048437903	0,843980537	-4,313903767

160	5	0	A_24_P85026	NM_032226	0,615164356	8,191591449	2,506895906	0,048482251	0,843980537	-4,314016957
159	47	0	A_23_P394043	NM_001080429	-0,366285975	7,501697926	-2,506854036	0,048484911	0,843980537	-4,314023741
175	76	0	A_24_P269062	NM_030964	-0,668394107	7,506734631	-2,506365074	0,048515977	0,843980537	-4,314102982
90	66	0	A_24_P415327	NM_000195	-0,432126326	8,005075694	-2,506202667	0,048526301	0,843980537	-4,314129305
450	20	0	A_23_P51215	NM_152372	0,617025223	5,945664033	2,506105091	0,048532504	0,843980537	-4,314145121
326	84	0	A_23_P69497	NM_003278	-0,415496077	6,700381026	-2,505959736	0,048541747	0,843980537	-4,314168682
170	80	0	A_23_P2967	NM_003917	-0,343288581	9,920812189	-2,505501619	0,04857089	0,843980537	-4,31424295
65	54	0	A_32_P214395	BX389966	-0,507754153	5,789567836	-2,505141155	0,048593833	0,843980537	-4,314301395
142	1	0	A_24_P465306	A_24_P465306	0,657993006	5,931188051	2,504901153	0,048609116	0,843980537	-4,314340314
295	69	0	A_32_P204959	THC2656116	0,355520169	6,107569526	2,504887313	0,048609997	0,843980537	-4,314342558
108	53	0	A_24_P936671	BC005220	-0,460786921	5,80982904	-2,504269185	0,048649382	0,843980537	-4,314442811
11	62	0	A_32_P200490	BM455175	-0,604591893	6,155741648	-2,504033745	0,048664393	0,843980537	-4,314481003
116	60	0	A_24_P153831	BC022004	-0,619303708	5,699227201	-2,503995166	0,048666853	0,843980537	-4,314487262
158	83	0	A_24_P312671	NM_005590	-0,614519599	6,783676927	-2,503970524	0,048668424	0,843980537	-4,314491259
412	69	0	A_24_P166397	NM_014809	-0,593532875	6,212602089	-2,503832784	0,048677209	0,843980537	-4,314513605
207	32	0	A_24_P402297	A_24_P402297	0,478430011	5,297455763	2,503602885	0,048691875	0,843980537	-4,314550904
150	26	0	A_24_P476247	NR_073552	0,403303242	5,945582426	2,5030834	0,048725032	0,843980537	-4,314635199
159	80	0	A_24_P453855	AK126267	-0,442340471	7,773871307	-2,502784264	0,048744136	0,843980537	-4,314683746
463	36	0	A_23_P27229	NM_016239	0,870644895	7,15243144	2,502262905	0,04877745	0,843980537	-4,314768373
455	46	0	A_23_P426021	NM_015187	0,432623132	8,430769217	2,501606288	0,048819443	0,843980537	-4,314874978
243	29	0	A_23_P138289	NM_003637	0,443328837	5,918805548	2,501374292	0,048834289	0,843980537	-4,314912651
6	46	0	A_23_P109269	NM_005560	-0,468515662	8,964912842	-2,501083279	0,048852918	0,843980537	-4,314959911
192	58	0	A_24_P922948	NM_001104595	-0,449184314	6,917923243	-2,500145566	0,048912998	0,843980537	-4,315112233
387	1	0	A_23_P369316	NM_138959	0,42990564	6,677319238	2,500115213	0,048914944	0,843980537	-4,315117164
262	4	0	A_24_P75708	A_24_P75708	0,491615806	7,470288254	2,499606299	0,048947585	0,843980537	-4,315199857
212	27	0	A_32_P475462	NM_006580	0,369933948	5,484947438	2,499481777	0,048955575	0,843980537	-4,315220092
515	73	0	A_23_P346384	NM_176792	-0,397072695	6,629653828	-2,498742281	0,049003054	0,843980537	-4,315340286
191	83	0	A_32_P117273	A_32_P117273	-0,497996944	6,838001999	-2,498306578	0,049031052	0,843980537	-4,31541112
37	53	0	A_23_P141100	NM_012091	-0,376295707	8,962430225	-2,498082437	0,049045461	0,843980537	-4,315447564
330	13	0	A_23_P134384	NM_014660	0,476198702	8,113339793	2,497820114	0,049062331	0,843980537	-4,31549022
141	67	0	A_24_P206305	NM_182924	-0,519404209	7,957653025	-2,497641525	0,049073819	0,843980537	-4,315519262
89	75	0	A_24_P841795	AK026403	-0,980775589	6,669029531	-2,49748801	0,049083697	0,843980537	-4,315544229
354	7	0	A_23_P77401	NM_018340	0,472522886	7,821498411	2,497144736	0,049105793	0,843980537	-4,315600062
271	36	0	A_24_P417935	NR_027032	-0,403702995	7,416997281	-2,496979861	0,049116409	0,843980537	-4,315626882
324	6	0	A_24_P290286	NM_182904	0,472386907	6,328631096	2,49660554	0,04914052	0,843980537	-4,315687777
161	72	0	A_23_P165295	NM_002501	-0,639441695	6,086526365	-2,496220581	0,04916533	0,843980537	-4,315750413
65	84	0	A_32_P172755	THC2668270	-0,801576326	6,212938683	-2,495945208	0,049183085	0,843980537	-4,315795223
33	54	0	A_23_P58031	NM_004721	-0,39647166	8,744434086	-2,495453643	0,049214797	0,843980537	-4,315875226
136	23	0	A_23_P127467	NM_032325	0,390224573	7,947327095	2,494915164	0,049249561	0,843980537	-4,315962883
341	12	0	A_23_P126499	NM_030907	0,4038565	6,731951305	2,494637148	0,049267519	0,843980537	-4,316008146
70	48	0	A_23_P84974	XM_005262105	-0,401946561	5,464483864	-2,494624021	0,049268368	0,843980537	-4,316010284
99	82	0	A_23_P68922	NM_033386	-0,679975409	7,537312183	-2,49412146	0,049300849	0,843980537	-4,316092119
6	62	0	A_32_P37988	THC2728454	-0,47978652	5,515552042	-2,494006035	0,049308312	0,843980537	-4,316110917
15	65	0	A_24_P929246	A_24_P929246	-0,85312566	5,557121309	-2,493594622	0,049334924	0,843980537	-4,316177924
17	70	0	A_32_P849727	AK057884	-0,679114936	5,265355332	-2,493481053	0,049342273	0,843980537	-4,316196424
69	47	0	A_24_P474188	TCONS_l2_00028242	-0,571305619	6,026078124	-2,493460879	0,049343578	0,843980537	-4,31619971
336	20	0	A_32_P78121	A_32_P78121	0,399781447	6,670224854	2,493225578	0,049358808	0,843980537	-4,316238041
377	11	0	A_32_P47361	THC2713677	0,532410663	5,941470702	2,493152805	0,049363519	0,843980537	-4,316249896
507	39	0	A_23_P153662	NM_203471	0,648122557	6,012382777	2,492341586	0,049416069	0,843980537	-4,316382076
197	69	0	A_24_P812543	THC2567642	-0,796157335	6,456814795	-2,492205007	0,049424923	0,843980537	-4,316404334
331	27	0	A_24_P73290	NM_001681	0,975997625	7,617240082	2,49200488	0,049437898	0,843980537	-4,316436951
186	80	0	A_24_P111019	BC028243	-1,3890162	6,70834561	-2,491814355	0,049450255	0,843980537	-4,316468005
198	83	0	A_24_P4816	NM_031412	-0,518146651	7,103147918	-2,491100157	0,049496603	0,843980537	-4,316584435
70	72	0	A_32_P224666	NM_006136	-0,972377682	5,498498467	-2,491034042	0,049500896	0,843980537	-4,316595214
165	33	0	A_23_P105118	NM_001005237	0,445082075	5,413216747	2,490898404	0,049509705	0,843980537	-4,316617331
120	36	0	A_24_P621023	ENST00000373816	0,38480612	9,338598209	2,490407304	0,049541611	0,843980537	-4,316697416
87	81	0	A_24_P925830	ENST00000370761	-0,819570837	6,228216481	-2,490341521	0,049545886	0,843980537	-4,316708144
290	47	0	A_23_P149852	NM_032429	-0,477713732	11,33166449	-2,488984435	0,049634177	0,843980537	-4,316929533
397	35	0	A_32_P176550	NM_152405	0,547020347	7,566024577	2,488340052	0,049676158	0,843980537	-4,317034695
105	25	0	A_23_P306890	NM_007195	0,516652627	7,902456173	2,488251547	0,049681927	0,843980537	-4,317049141
352	72	0	A_23_P4161	ENST00000448504	-0,591857344	6,911545043	-2,488211864	0,049684514	0,843980537	-4,317055619
451	49	0	A_24_P110983	NM_005465	0,405202508	10,87010483	2,48766426	0,049720227	0,843980537	-4,317145012
402	46	0	A_23_P425990	NM_001033602	0,723114098	5,625943803	2,487643776	0,049721563	0,843980537	-4,317148357
196	5	0	A_24_P717262	A_24_P717262	0,684178446	5,837275511	2,487371411	0,049739337	0,843980537	-4,317192826
507	74	0	A_32_P32537	A_32_P32537	-1,062522178	6,104300282	-2,487196868	0,04975073	0,843980537	-4,317221327
493	80	0	A_23_P50967	NM_020760	-0,583002422	6,117187949	-2,485888515	0,049836222	0,843980537	-4,317435025
532	67	0	A_24_P216501	NM_002735	-0,37175449	7,909089323	-2,485869773	0,049837448	0,843980537	-4,317438087
312	17	0	A_24_P517252	NM_001077620	0,463689581	6,607566439	2,48548977	0,049862308	0,843980537	-4,317500175
143	15	0	A_32_P25357	NM_004933	0,608644523	6,713362382	2,485435531	0,049865858	0,843980537	-4,317509038
323	2	0	A_23_P4561	NM_198833	0,772091941	6,893397237	2,485413866	0,049867276	0,843980537	-4,317512578
509	41	0	A_23_P88184	NM_018167	0,468616692	6,730900902	2,485190268	0,049881912	0,843980537	-4,317549117
514	65	0	A_23_P406521	NM_178460	0,339605756	6,784264732	2,485045942	0,049891361	0,843980537	-4,317572703
69	55	0	A_24_P259083	NM_001040022	-0,582914979	5,724351662	-2,484822502	0,049905995	0,843980537	-4,317609221
366	43	0	A_24_P184692	NM_003317	0,527304501	6,30194692	2,484227805	0,049944964	0,843980537	-4,317706431
261	82	0	A_32_P209770	XM_005256738	-0,594161513	6,946071782	-2,483472563	0,0499945	0,843980537	-4,317829917
144	67	0	A_23_P59418	NM_005011	-0,342814127	6,889199139	-2,483446693	0,049996198	0,843980537	-4,317834147
